# Functionalization of the imidazo[1,2-*a*]pyridine ring in α-phosphonoacrylates and α-phosphonopropionates via microwave-assisted Mizoroki–Heck reaction

**DOI:** 10.3762/bjoc.16.3

**Published:** 2020-01-03

**Authors:** Damian Kusy, Agata Wojciechowska, Joanna Małolepsza, Katarzyna M Błażewska

**Affiliations:** 1 Institute of Organic Chemistry, Faculty of Chemistry, Lodz University of Technology, 116 Zeromskiego St, 90–924 Lodz, Poland

**Keywords:** α-phosphonoacrylates, α-phosphonopropionates, imidazo[1,2-*a*]pyridine, microwave-assisted reaction, Mizoroki–Heck reaction

## Abstract

A series of new phosphonocarboxylates containing an imidazo[1,2-*a*]pyridine ring has been synthesized via the microwave-assisted Mizoroki–Heck reaction. The efficient modification of the imidazo[1,2-*a*]pyridine ring has been achieved as late-stage functionalization, enabling and accelerating the generation of a library of compounds from a common precursor.

## Introduction

In the last few decades, the Mizoroki–Heck reaction has become one of the main tools in organic synthesis. Its use for the functionalization of a wide range of compounds cannot be overrated [1]. However, as is true for many reactions, the late-stage functionalization of target compounds or their advanced intermediates is rarely an extension of reaction conditions developed for structurally simple analogs. The late-stage approach is especially desirable in the synthesis of a library of compounds from a common precursor and enables efficient generation of new chemical entities.

Imidazo[1,2-*a*]pyridine constitutes an appealing scaffold in medicinal chemistry. It is present in a number of compounds, which exhibit many interesting biological properties. Its privileged character is confirmed by the presence in a number of pharmaceuticals [2–3]. The imidazo[1,2-*a*]pyridine-ring modification via Pd-catalyzed reactions is broadly reported in the literature [3], however, such functionalizations for more complex molecules are not very common. On the other hand, the Mizoroki–Heck reaction is rarely applied for the functionalization of molecules bearing phosphonate or phosphine oxide groups, and the known reactions involve high temperatures (≈100 °C) and long reaction times (in most cases >24 h) [4–7].

Herein we disclose the first method for functionalization of molecules composed of both, an imidazo[1,2-*a*]pyridine ring and the phosphoryl group. The combination of these functional groups can be found in bisphosphonates and phosphonocarboxylates [8–10] – inhibitors of the therapeutically important enzymes, farnesyl pyrophosphate synthase (FPPS) and Rab geranylgeranyl transferase (RGGT), respectively.

## Results and Discussion

**Figure 1:**
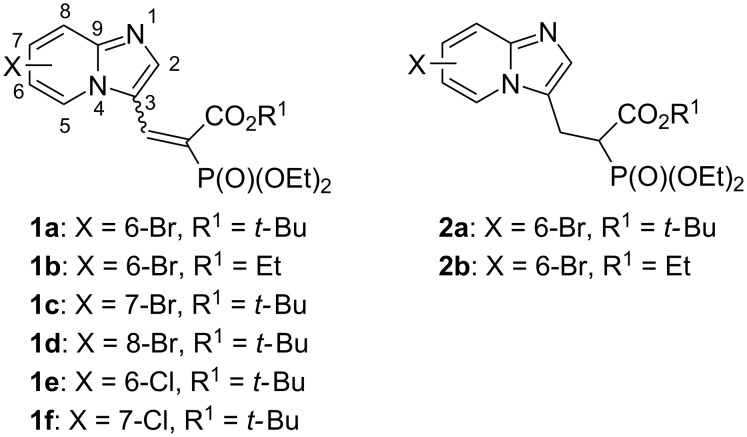
Substrates used for the Mizoroki–Heck reaction in this study.

We have previously synthesized a number of phosphonocarboxylates (PC) to study their structure–activity relationship with RGGT, using compounds **1** and **2** as advanced intermediates in the synthesis of RGGT inhibitors (Figure 1) [10–11]. We have found that the presence of a substituent at C6 position of the imidazo[1,2-*a*]pyridine is favorable for retaining the activity of PC toward RGGT. Such substituents were introduced into the heterocyclic ring via the use of appropriately substituted commercially available 2-aminopyridines. Here, we propose a new and efficient strategy, by which structurally diverse substituents can be attached to the imidazo[1,2-*a*]pyridine ring, being a part of common precursors, compounds **1** and **2**.

The Mizoroki–Heck reaction-assisted functionalization of 6-bromoimidazo[1,2-*a*]pyridine-3-carbaldehyde [12], a precursor of compounds **1** and **2**, was successfully achieved. We used tri(*o*-tolyl)phosphine (P(*o*-tol)_3_, palladium(II) acetate (Pd(OAc)_2_) and obtained the desired product (*E*)-benzyl 3-(6-aminopyridin-3-yl)acrylate in 86% yield (see Supporting Informaiton File 1). However, when the same conditions were applied to alkyl 3-(6-bromoimidazo[1,2-*a*]pyridin-3-yl)-2-(diethoxyphosphoryl)acrylates **1**, no desired product **3** was formed under standard heating conditions (Table 1, entry 1).

**Table 1:** Summary of optimization studies.

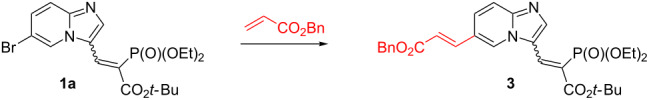							

Entry^a^	Base	Ligand	Pd catalyst	Solvent	*T* [°C]	*t* [min]	Yield^b^

**1**^c^	DIPEA	P(*o*-tol)_3_	Pd(OAc)_2_	PCN	97	24 h	0
**2**^c^	DIPEA	P(*o*-tol)_3_	Pd(OAc)_2_	PCN	100	40	70^d,e^
**3**^f^	DIPEA	P(*o*-tol)_3_	Pd(OAc)_2_	PCN	110	50	58
**4**^g^	DIPEA	P(*o*-tol)_3_	Pd(OAc)_2_	PCN	110	50	74
**5**	DIPEA	P(*o*-tol)_3_	Pd(OAc)_2_	PCN	110	50	69
**6**	DIPEA	P(*o*-tol)_3_	Pd(OAc)_2_	PCN	150	50	–^h^
**7**	DIPEA	P(*o*-tol)_3_	Pd(OAc)_2_	PCN	130	10	51
**8**	DIPEA	P(*t-*Bu)_3_	Pd(OAc)_2_	PCN	110	50	58
**9**	DIPEA	DABCO	Pd(OAc)_2_	PCN	110	50	49
**10**	DIPEA	P(Cy)_3_	Pd(OAc)_2_	PCN	110	50	0^e^
**11**	DIPEA	–	–	PCN	110	30	0
**12**	DIPEA	–	Pd(OAc)_2_	PCN	110	30	47^i^
**13**	DIPEA	–	Pd(PPh_3_)_4_	PCN	110	30	17^j^
**14**	DIPEA	–	Pd(dba)_2_	PCN	110	30	42^j^
**15**	TEA	P(*o*-tol)_3_	Pd(OAc)_2_	PCN	110	50	62
**16**	Cy_2_NH	P(*o*-tol)_3_	Pd(OAc)_2_	PCN	110	50	67
**17**	Cy_2_NMe	P(*o*-tol)_3_	Pd(OAc)_2_	PCN	110	50	62
**18**	(*n*-Bu)_3_N	P(*o*-tol)_3_	Pd(OAc)_2_	PCN	110	50	69
**19**	NMM	P(*o*-tol)_3_	Pd(OAc)_2_	PCN	110	30	32^j^
**20**	K_2_CO_3_	P(*o*-tol)_3_	Pd(OAc)_2_	PCN	110	30	13^j^
**21**	Cs_2_CO_3_	P(*o*-tol)_3_	Pd(OAc)_2_	PCN	110	30	4^j^
**22**	**DIPEA**	**P(*o*-tol)_3_**	**Pd(OAc)_2_**	**PCN**	**110**	**30**	**91**
**23**	DIPEA	P(*o*-tol)_3_	Pd(OAc)_2_	PCN/H_2_O 100:1	110	50	70
**24**	DIPEA	P(*o*-tol)_3_	Pd(OAc)_2_	PCN/H_2_O 2:1	110	30	14^h^
**25**	DIPEA	P(*o*-tol)_3_	Pd(OAc)_2_	DMF/H_2_O 1:1	110	30	0
**26**	DIPEA	P(*o*-tol)_3_	Pd(OAc)_2_	EtOH/H_2_O 2:1	110	30	0
**27**	DIPEA	P(*o*-tol)_3_	Pd(OAc)_2_	dioxane/EtOH 1:1	110	30	80
**28**	DIPEA	P(*o*-tol)_3_	Pd(OAc)_2_	dioxane/EtOH 1:1	110	40	69
**29**	Na_2_CO_3_	–	Pd(PPh_3_)_4_	toluene/EtOH/H_2_O 2:1:2	80	25	0^d,e^

^a^The ratio for most experiments (except for those indicated below): substrate/olefin/ligand/Pd(OAc)_2_ = 1:1.1:0.05:0.045. ^b^Yield of the isolated compound, if not stated otherwise. ^c^Substrate/olefin/ligand/Pd(OAc)_2_ = 1:1.5:0.1:0.18. ^d^The given value represents the degree of conversion. ^e^Substrate recovered. ^f^Substrate/olefin/ligand/Pd(OAc)_2_ = 1:1.5:0.2:0.18. ^g^Substrate/olefin/ligand/Pd(OAc)_2_ = 1:1.5:0.05:0.045. ^h^Unidentified products of decomposition. ^i^Fifty-eight percent conversion into product were observed after 30 min. A comparable result was obtained after another 30 min under microwave heating. ^j^This substrate is the main component of the reaction mixture; the yield was estimated based on ^31^P NMR.

Recently, multiple variants for the application of microwave heating for conducting Mizoroki–Heck reactions have been reported, including the use of heterogeneous and homogeneous catalysts, as well as continuous-flow conditions [13–15]. Therefore, we tested microwave heating, a technique which is known for significant acceleration of the C–C coupling reaction [16]. We carried out the optimization studies using model substrates – compound **1a** and benzyl acrylate (Table 1). Compound **1a** was prepared according to our previously published procedure (see Supporting Information File 1) [10] as a mixture of diastereomers (*E*/*Z* ratio was determined by ^31^P NMR; the configuration of the double bond C*H*=C*P* was determined by ^1^H NMR vicinal coupling constants ^3^*J*_HP_ [10,17]). However, we did not observe any change in this ratio under the applied conditions of the Mizoroki–Heck reaction.

On applying microwave irradiation (100 °C, 40 min), we achieved 70% conversion into product **3** (Table 1, entry 2). In order to improve the conversion into the product, we increased the reaction temperature to 110 °C and prolonged the time to 50 min (Table 1, entry 3) and obtained product **3** with 58% yield. Then, we reduced the loading of the ligand, P(*o*-tol)_3_ and catalyst Pd(OAc)_2_, to one fourth of the loading used in the previous experiment which improved the yield of the isolated product **3** to up to 74% (Table 1, entry 4). Furthermore, reducing the amount of acrylate from 1.5 to 1.1 equiv led to a slight decrease in the yield (from 74% to 69%), but facilitated the purification process (Table 1, entry 5). Therefore, we established 110 °C as the optimal temperature for this reaction, because higher temperatures led to decomposition of the reagents (Table 1, entries 6 and 7), and full conversion into the product was not achieved at lower temperatures (Table 1, entry 2).

The use of other ligands resulted in a decreased yield (for tri-*tert*-butylphosphine, (P(*t*-Bu)_3_) or DABCO), or even complete lack of conversion as in the case of tricyclohexylphosphine (P(Cy)_3_ (Table 1, entries 8–10). Also no reaction was observed in the absence of Pd(OAc)_2_ and P(*o*-tol)_3_, while partial conversion into product **3** occurred in the presence of Pd(OAc)_2_ (Table 1, entries 11 and 12). In addition, replacing palladium acetate by tetrakis(triphenylphosphine)palladium(0), Pd(PPh_3_)_4_, or bis(dibenzylideneacetone)palladium(0), Pd(dba)_2_, resulted in a reduction in the yield and a partial recovery of substrate **1a** (Table 1, entries 13 and 14).

In the next series of experiments, different amines were tested. The use of triethylamine, dicyclohexylamine (Cy_2_NH), dicyclohexylmethylamine (Cy_2_NMe), and tributylamine (*n*-Bu_3_N) led to 62–69% yields, while for *N-*methylmorpholine (NMM), the yield dropped to 32% (Table 1, entries 15–19). The use of inorganic bases also led to very low yields (4–13%) and recovery of substrate (Table 1, entries 20 and 21). *N,N*-Diisopropylethylamine (DIPEA) turned out to be the amine of choice (Table 1, entry 5, yield 69%), because, in most cases, it could be readily separated after the reaction.

A significant improvement was achieved after decreasing the reaction time from 50 to 30 min, which led to 91% yield (Table 1, entry 22). The shorter exposure to microwave heating probably limited the decomposition of the substrate and the product.

**Scheme 1:**
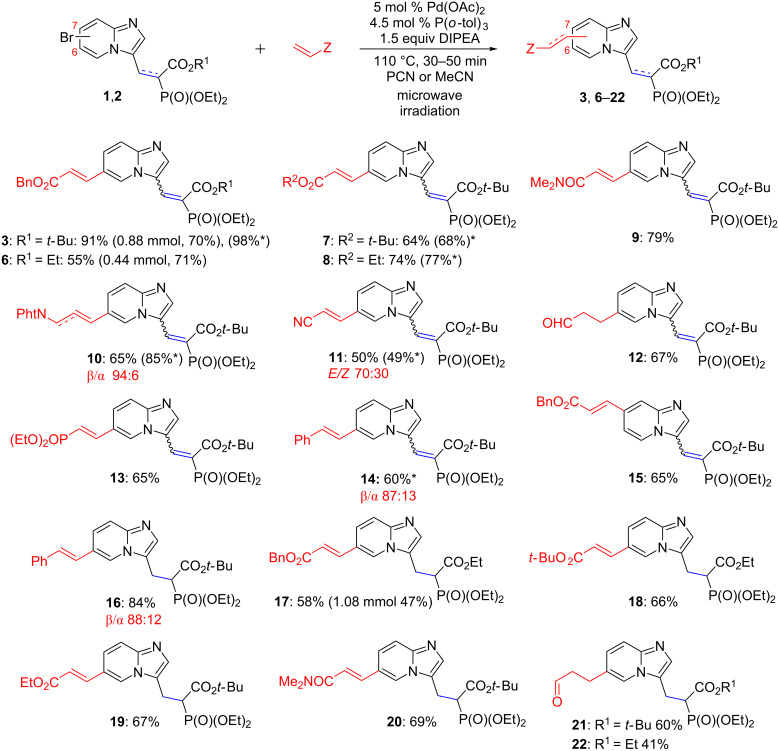
Scope of the method for analogs derived from **1** and **2**. The ratio of isomers is given (*E*/*Z* or β/α) in red, wherever applicable. Yields are given for reactions run at a 100 mg (0.22 mmol) scale, except for compounds **3** (0.88 mmol), **6** (0.44 mmol), **18** (0.11 mmol, 40 min, 100 °C), and **17** (1.08 mmol) as indicated below the appropriate structures. Asterisks indicate yields for the reaction carried out in acetonitrile.

Among the various choices of solvents, propionitrile (PCN) delivered the highest amount of product **3**, while other solvents led to lower yields (Table 1, entries 23–29). As we have confirmed on selected examples of substrates, the reaction can be also run in acetonitrile (49–98%), with a yield similar to that in the reaction in PCN (50–91%, Scheme 1). The presence of a little amount of water did not impair the reaction (Table 1, entry 23). However, when water was used as co-solvent, the yield significantly dropped, or no product was detected at all (Table 1, entries 24–26).

Overall, the optimal conditions for the Mizoroki–Heck reaction of compound **1a** and benzyl acrylate were established to involve 5 mol % of Pd(OAc)_2_, 4.5 mol % of P(*o*-tol)_3_, and DIPEA in PCN (or acetonitrile), under microwave heating conditions for 30 min at 110 °C (Table 1, entry 22).

**Figure 2:**
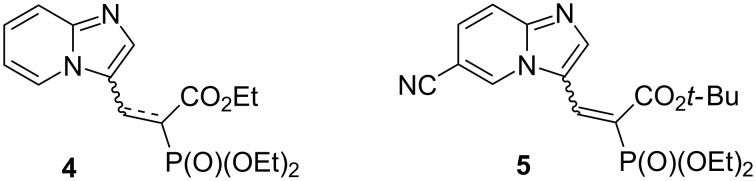
Structures of the identified side products **4** and **5**.

During our optimization studies, we also identified two side products (Figure 2), that were detected in up to 6% each. They were isolated and their identities were confirmed by NMR, MS, and IR analyses. Compound **4** is the product of dehalogenation reaction, which is known to be catalyzed by palladium [1,18]. The second side product, compound **5**, is probably the result of the cleavage of the C–CN bond in the solvent (propionitrile or acetonitrile), followed by a palladium-catalyzed cyanation of substrate **1**. Recently, acetonitrile was reported as a nontoxic source of cyanide [19–20].

Having the optimized conditions in hand, the scope of this transformation was examined using selected olefins and the substrate containing imidazo[1,2-*a*]pyridine ring embedded either within 2-(diethoxyphosphoryl)acrylate (analogs **1**) or 2-(diethoxyphosphoryl)propanoates (analogs **2**).

The reaction worked well for a number of acrylates (Scheme 1), providing predominantly products with (*E*)-geometry of the newly formed double bond (*E/Z* ratio is above 98:2). Only for analog **11**, the formation of the (*Z*)-product was observed in up to 30%. The configuration of the newly formed double bond was determined based on the vicinal coupling constant ^3^*J*_HH_ between C*H*=C*H* [(*E*)-isomer: ^3^*J*_HH_ = 16.5 Hz, (*Z*)-isomer: ^3^*J*_HH_ = 12.1 Hz].

We also showed that the method is applicable to styrene and phthalylallylamine, although, for these substrates, a mixture of regioisomers, resulting from α- and β-arylation, was formed (see Supporting Information File 1, Figures S16 and S17, S32 and S33) [21]. In case of the reaction with allyl alcohol, the generated products isomerized in situ to give the saturated aldehydes, compounds **12**, **21**, and **22**, a result being consistent with analogous reactions reported in the literature [22–23].

In the reaction with acrylic acid and acrylamide, substrate **1a** was recovered, probably due to the propensity of these acrylic substrates to polymerization. In case of ethyl crotonate and methyl metacrylate, only traces of the desired products were formed. The structures of products **3** and **6–22** were confirmed by spectroscopic methods, ^1^H, ^13^C, ^31^P NMR, and MS analysis. The stereochemistry of the newly formed double bond was evaluated by ^1^H NMR spectroscopy.

Next, we determined the influence of the position of a halogen substituent in the imidazo[1,2-*a*]pyridine ring on its reactivity in the Mizoroki–Heck reaction. We confined our studies to the analogs bearing substituents at positions 6, 7, and 8 of the six-membered ring of the imidazo[1,2*-a*]pyridine (**1a–f**, in Figure 1) [24]. We found that the reaction occurred for the 6- and 7-substituted analogs **1a–c**, but no product was observed in the reaction with the 8-halogeno-imidazo[1,2-*a*]pyridine analog **1d** [25]. As the Mizoroki–Heck reaction is sensitive to the electronic properties of the aryl halides, with electron-withdrawing substituents imparting a smaller barrier in the oxidative addition step [26–27], we envisioned that the C8 carbon could be more electron rich than C6 and C7, possibly due to the proximity of the nitrogen atom N1. All the above reactions were run on bromine-substituted analogs. Analogs bearing a strong carbon–chlorine bond did not react, as was observed for substrates **1e** and **1f**.

**Scheme 2:**
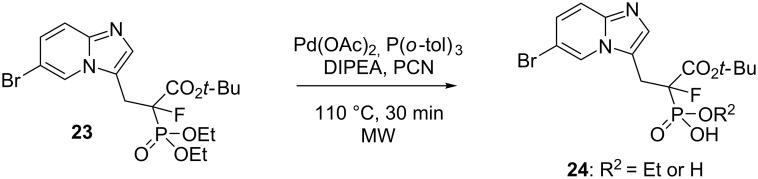
Dealkylation of fluorinated analog **23** under the Mizoroki–Heck reaction conditions.

Finally, we checked whether the method could be extended to fluorinated analog **23** (Scheme 2). Unfortunately, in this case, the desired product constituted no more than 20% of the reaction mixture, while the main components were the product of dealkylation of the phosphorous ester group, compound **24**, and the mixture of substrate **23** and the desired product of the Mizoroki–Heck reaction (Scheme 2 and Figures S69–S71 in Supporting Information File 1).

## Conclusion

In conclusion, we have developed a convenient protocol for the functionalization of alkyl 3-(bromoimidazo[1,2-*a*]pyridin-3-yl)-2-(diethoxyphosphoryl)acrylates **1** and propionates **2** using microwave-assisted Mizoroki–Heck reaction. The method worked for a number of olefins, enabling the functionalization of the heterocyclic ring with diverse groups, which can be used for further modifications. Importantly, synthesis of these compounds by standard thermal Heck protocols was ineffective, thus highlighting the importance of employing microwave irradiation for this transformation. Eighteen new compounds were obtained using the developed method. The advantages of the proposed method include operational simplicity, high diastereoselectivity, the wide substrate scope and its applicability to smaller (0.11 mmol) and larger scales (1.1 mmol).

## Supporting Information

File 1 Full experimental details, including copies of spectra (^1^H NMR, ^13^C NMR, ^31^P NMR) of all new compounds. The experimental details and NMR description of the starting compounds synthesized according to our previously published procedure.
