# Supplementary material for: Functionalization of the imidazo[1,2-a]pyridine ring in α-phosphonoacrylates and α-phosphonopropionates via microwave-assisted Mizoroki–Heck reaction
Source: Beilstein J Org Chem. 2020 Jan 3;16:15–21. doi: 10.3762/bjoc.16.3 (PMC6964661; doi:10.3762/bjoc.16.3)
Supplement: File 1 — Full experimental details, including copies of spectra (1H NMR, 13C NMR, 31P NMR) of all new compounds. The experimental details and NMR description of the starting compounds synthesized according to our previously published procedure. [file Beilstein_J_Org_Chem-16-15-s001.pdf]

BEILSTEIN JOURNAL OF ORGANIC CHEMISTRY

Supporting Information

for

Functionalization of the imidazo[1,2-*a*]pyridine ring in α-phosphonoacrylates and α-phosphonopropionates via microwave-assisted Mizoroki–Heck reaction

Damian Kusy, Agata Wojciechowska, Joanna Małolepsza and Katarzyna M. Błażewska

Beilstein J. Org. Chem. **2020,** *16,* 15–21. doi:10.3762/bjoc.16.3

Full experimental details, including copies of spectra (^{1}H NMR, ^{13}C NMR, ^{31}P NMR) of all new compounds. The experimental details and NMR description of the starting compounds synthesized according to our previously published procedure

License and Terms: This is a supporting information file under the terms of the Creative Commons Attribution License (https://creativecommons.org/licenses/by/4.0). Please note that the reuse, redistribution and reproduction in particular requires that the authors and source are credited.
The license is subject to the *Beilstein Journal of Organic Chemistry* terms and conditions: (https://www.beilstein-journals.org/bjoc)

Table of Contents

1. General information S3

General procedure for the synthesis of compounds **3**, **6–22** S4

2. Characterization data, copies of ^{1}H NMR, ^{13}C NMR and ^{31}P NMR spectra S5

Figure S1. ^{1}H NMR of compound **3** (700 MHz, $CDCl_3$) S5

Figure S2. ^{31}P NMR of compound **3** (ratio *E*/*Z* 100:3) (283 MHz, $CDCl_3$). S6

Figure S3. ^{13}C NMR of compound **3** (176 MHz, $CDCl_3$) S6

Figure S4. ^{1}H NMR of compound **6** (700 MHz, $CDCl_3$) S7

Figure S5. ^{31}P NMR of compound **6** (ratio *E*/*Z* 100:3) (283 MHz, $CDCl_3$) S8

Figure S6. ^{13}C NMR of compound **6** (176 MHz, $CDCl_3$) S8

Figure S7. ^{1}H NMR of compound **7** (700 MHz, $CDCl_3$) S9

Figure S8. ^{31}P NMR of compound **7** (ratio *E*/*Z* 100:6) (283 MHz, $CDCl_3$) S10

Figure S9. ^{13}C NMR of compound **7** (176 MHz, $CDCl_3$) S10

Figure S10. ^{1}H NMR of compound **8** (700 MHz, $CDCl_3$) S11

Figure S11. ^{31}P NMR of compound **8** (ratio *E*/*Z* 100:11) (283 MHz, $CDCl_3$) S12

Figure S12. ^{13}C NMR of compound **8** (176 MHz, $CDCl_3$) S12

Figure S13. ^{1}H NMR of compound **9** (700 MHz, $CDCl_3$) S13

Figure S14. ^{31}P NMR of compound **9** (ratio *E*/*Z* 100:15) (283 MHz, $CDCl_3$) S14

Figure S15. ^{13}C NMR of compound **9** (176 MHz, $CDCl_3$) S14

Figure S16. ^{1}H NMR of compound **10** (700 MHz, $CDCl_3$) S16

Figure S17. ^{31}P NMR of compound **10** (ratio *E*/*Z* 100:4) (283 MHz, $CDCl_3$) S16

Figure S18. ^{13}C NMR of compound **10** (176 MHz, $CDCl_3$) S17

Figure S19. Composition of the reaction mixture (compound **11**) S18

Figure S20. ^{1}H NMR of compound **11** (700 MHz, $CDCl_3$) S19

Figure S21. ^{31}P NMR of compound **11** (283 MHz, $CDCl_3$) S19

Figure S22. ^{13}C NMR of compound **11** (176 MHz, $CDCl_3$) S20

Figure S23. ^{1}H NMR of compound **12** (residual DIPEA·HBr at 1.5, 3.1, 3.6 ppm) (700 MHz, $CDCl_3$) S21

Figure S24. ^{31}P NMR of compound **12** (ratio *E*/*Z* 100:16) (283 MHz, $CDCl_3$) S21

Figure S25. ^{13}C NMR of compound **12** (176 MHz, $CDCl_3$) S22

Figure S26. ^{1}H NMR of compound **13** (700 MHz, $CDCl_3$) S23

Figure S27. ^{31}P NMR of compound **13** (283 MHz, $CDCl_3$) S23

Figure S28. ^{13}C NMR of compound **13** (176 MHz, $CDCl_3$) S24

Figure S29. ^{1}H NMR of compound **15** (700 MHz, $CDCl_3$) S25

Figure S30. ^{31}P NMR of compound **15** (283 MHz, $CDCl_3$) S25

Figure S31. ^{13}C NMR of compound **15** (176 MHz, $CDCl_3$) S26

Figure S32. ^{1}H NMR of compound **14** (700 MHz, $CDCl_3$) S27

Figure S33. ^{31}P NMR of compound **14** (mixture of Heck products) (283 MHz, $CDCl_3$) 27

Figure S34. ^{13}C NMR of compound **14** (176 MHz, $CDCl_3$) S28

Figure S35. ^{1}H NMR of compound **16** (700 MHz, $CDCl_3$) S29

Figure S36. ^{31}P NMR of compound **16** (mixture of Heck products) (283 MHz, $CDCl_3$) S29

Figure S37. ^{13}C NMR of compound **16** (176 MHz, $CDCl_3$) S30

Figure S38. ^{1}H NMR of compound **17** (700 MHz, $CDCl_3$) S31

Figure S39. ^{31}P NMR of compound **17** (283 MHz, $CDCl_3$) S31

Figure S40. ^{13}C NMR of compound **17** (176 MHz, $CDCl_3$) S32

Figure S41. ^{1}H NMR of compound **18** (700 MHz, $CDCl_3$) S33

Figure S42. ^{31}P NMR of compound **18** (286 MHz, $CDCl_3$) S33

Figure S43. ^{13}C NMR of compound **18** (176 MHz, $CDCl_3$) S34

Figure S44. ^{1}H NMR of compound **19** (700 MHz, $CDCl_3$) S35

Figure S45. ^{31}P NMR of compound **19** (284 MHz, $CDCl_3$) S35

Figure S46. ^{13}C NMR of compound **19** (176 MHz, $CDCl_3$) S36

Figure S47. ^{1}H NMR of compound **20** (700 MHz, $CDCl_3$) S37

Figure S48. ^{31}P NMR of compound **20** (283 MHz, $CDCl_3$) S37

Figure S49. ^{13}C NMR of compound **20** (176 MHz, $CDCl_3$) S38

Figure S50. ^{1}H NMR of compound **21** (residual DIPEA·HBr at 1.5, 3.1, 3.6 ppm) (700 MHz, $CDCl_3$) S39

Figure S51. ^{31}P NMR of compound **21** (283 MHz, $CDCl_3$) S39

Figure S52. ^{1}H NMR of compound **22** (700 MHz, $CDCl_3$) S41

Figure S53. ^{31}P NMR of compound **22** (283 MHz, $CDCl_3$) S41

Figure S54. ^{13}C NMR of compound **22** (176 MHz, $CDCl_3$) S42

Figure S55. ^{1}H NMR of compound **4** (700 MHz, $CDCl_3$) S43

Figure S56. ^{31}P NMR of compound **4** (283 MHz, $CDCl_3$) S43

Figure S57. ^{1}H NMR of compound **5** (700 MHz, $CDCl_3$) ... S44

Figure S58. ^{31}P NMR of compound **5** (283 MHz, $CDCl_3$) ... S45

Figure S59. ^{13}C NMR of compound **5** (176 MHz, $CDCl_3$)\ ... S45

Figure S60. Infrared spectroscopy of **5** ... S46

Side product **5** obtained via different synthetic route [1] ... S46

Figure S61. ^{1}H NMR of compound **5’** (700 MHz, $CDCl_3$) ... S47

Figure S62. ^{31}P NMR of compound **5’** (283 MHz, $CDCl_3$) ... S48

Figure S63. ^{13}C NMR of compound **5’** (176 MHz, $CDCl_3$) ... S48

Figure S64. Comparing the ^{13}C NMR spectra of compounds **5** (blue) and **5’** (red) ... S49

Figure S65. Comparing the ^{1}H NMR spectra of compounds **5** (blue) and **5’** (red) ... S49

Figure S66. ^{1}H NMR spectra of mixture of side product **5** and synthesized compound **5’** ... S50

Figure S67. ^{1}H NMR of compound **23** (700 MHz, $CDCl_3$) ... S53

Figure S68. ^{31}P NMR of compound **23** (283 MHz, $CDCl_3$ ... S53

Figure S69. Comparing ^{31}P NMR spectra of reaction mixture after Heck reaction performed on compound **23**. ... S54

Figure S70. Comparing ^{1}H NMR spectra of reaction mixture after Heck reaction performed on compound **23** ... S55

Figure S71. Comparing ^{1}H NMR spectra of reaction mixture after Heck reaction performed on compound **23** (range 0–4.7) ... S55

General procedure for the synthesis of starting materials (**1**,**2**, and **23**) [1] ... S56

*General procedure for the synthesis of compounds (**1**).* ... S56

*General procedure for the synthesis of compounds (**2**).* ... S57

*General procedure for the synthesis of compounds (**23**).* ... S58

Figure S72. ^{1}H NMR of compound **23** (250 MHz, $CDCl_3$) ... S59

Figure S73. ^{31}P NMR of compound **23** (101 MHz, $CDCl_3$) ... S60

References ... S60

1. General information

NMR spectra were measured at 250.13 or 700 MHz for ^{1}H NMR, 62.90 or 170 MHz for ^{13}C NMR, 283 or 101.30 MHz for ^{31}P NMR on Bruker Avance DPX 250 and Bruker Avance II Plus 700 spectrometers, respectively. Chemical shifts (δ) are reported in parts per million (ppm) relative to internal residual $CHCl_3$ in $CDCl_3$ (δ 7.26 ^{1}H NMR), $CDCl_3$ signal in ^{13}C NMR (δ 77.16) or external 85% H_3PO_4 (δ 0 ppm ^{31}P NMR). ^{31}P NMR and ^{13}C NMR spectra were proton-decoupled. Coupling constants (*J*) are quoted in Hz. The assignment of the signals in ^{1}H NMR and ^{13}C NMR spectra was supported by two-dimensional experiments

(COSY, HMQC, HMBC, DEPT-135). In the case of compounds obtained as diastereomeric mixtures, the ratio of diastereomers was determined based on ^{31}P NMR and (if possible) sufficiently separated signals in ^{1}H NMR. IR spectra were measured on an FT-IR Alpha Bruker (ATR) instrument and were reported in cm^{-1}.
A monomode microwave reactor (CEM Discover SP) equipped with an IntelliVent pressure control system was used. The standard method was applied and maximum pressure was set to 250 psi. Temperatures of the reaction mixtures were measured with an external infrared sensor.
Samples were analyzed in positive ion mode with an Agilent 6220 mass spectrometer coupled with an Agilent 1200 series HPLC. Three µL of the sample were injected at a flow rate of 200 µL/min, methanol + 0.1% formic acid. Mass spectrometry parameters were as follows: *m*/*z* range 100–3000, fragmentor 350 V, skimmer 65 V, gas temp 325 °C, drying gas 8 L/min, nebulizer 45 psig, capillary 4000 V.
The spectra of the main fractions after flash chromatography purification were given. The impurities are indicated and defined in the spectra.
The reactions were carried out using substrate **1**, which was in most cases used as the mixture of (*E*) and (*Z*)-stereoisomers (typically up to 85:15 ratio). Therefore, in the ^{31}P NMR spectra of products derived from **1**, a mixture of (*E*) and (*Z*) products is present, as indicated for the example of compound **3**. The ratio is also included in the description of each compound. However, the presence of *E/Z*-stereoisomers is due to the stereochemistry of the double bond already present in the substrate **1** and has nothing to do with the stereochemistry of the newly formed double bond. In most examples, the double bond formed in the reaction is *E*-configured ((*E*) >98%). The isomers were identified and their ratio determined based on the ^{31}P NMR spectra and the values of vicinal coupling constants in the ^{1}H NMR spectra: $^{3}J_{HP}$ (for substrates **1**) and $^{3}J_{HP}$/$^{3}J_{HH}$ (for products). It has been presented on the example of compound **11** (see Figure S19 and pages S19,S20). The chemical shifts in ^{13}C and ^{1}H NMR are reported only for the major isomers. The chemical shifts in ^{31}P NMR are given for both isomers.

General procedure for the synthesis of compounds 3, 6–22

$Pd(OAc)_2$ (2.45 mg, 0.0108 mmol, 0.05 equiv) and tri(*o*-tolyl)phosphine (2.98 mg, 0.0098 mmol, 0.045 equiv) were added to a solution of **1a** (0.2177 mmol, 1.0 equiv), DIPEA (0.057 mL, 0.327 mmol, 1.5 equiv), benzyl acrylate (0.036 mL, 0.24 mmol, 1.1 equiv) in PCN (2 mL), and the mixture placed in pressure vial equipped with a magnetic stirring bar. The mixture was stirred for 1 min and purged with argon via a syringe. Then, MW irradiation (with initial 150 W power) was applied. The following conditions were used: 30 min, 110 °C. Afterwards, the reaction mixture was diluted with DCM (10 mL), and adsorbed on silica gel (≈ 3 g). The solvent was evaporated, and the residue was subjected to column chromatography using gradient DCM/acetone as eluent to give the product as orange oil. Depending on the products different gradients were used: for compounds **3**, **6**–**8**, **10**–**15** (100:0 => 70:30, for compounds **16**–**19**, **21**–**22** gradient 100:0 => 40:60, for compounds **9** and **20** gradient 100:0 => 10:90).

2. Characterization data, copies of ^{1}H NMR, ^{13}C NMR and ^{31}P NMR spectra

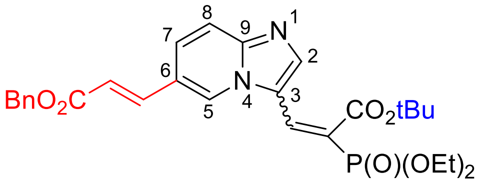

3

***tert*-Butyl 3-(6-((*E*)-3-(benzyloxy)-3-oxoprop-1-en-1-yl)imidazo[1,2-*a*]pyridin-3-yl)-2-(diethoxyphosphoryl)acrylate (3).** Yield 91%

HRMS m/z: calculated 541.2098 ($C_{28}H_{33}N_2O_7P$ + H)$^+$, found 541.2104 ($C_{28}H_{33}N_2O_7P$ + H)$^+$, **^{1}H NMR** (700 MHz, $CDCl_3$) δ: 1.36 (t, $^{3}J_{HH}$ = 7.1 Hz, CH_3CH_2OP, 6H), 1.56 (s, $C(CH_3)_3$, 9H), 4.06 – 4.24 (m, CH_3CH_2OP, 4H), 5.26 (s, $PhCH_2O$, 2H), 6.52 (d, $^{3}J_{HH}$ = 15.9 Hz, BnOC(O)CH=CH, 1H), 7.36 – 7.44 (m, $PhCH_2O$, 5H), 7.55 (dd, $^{3}J_{HH}$ = 9.4 Hz, $^{4}J_{HH}$ = 1.4 Hz, $CH_{(7)}$, 1H), 7.68 (d, $^{3}J_{HH}$ = 9.4 Hz, $CH_{(8)}$, 1H), 7.70 (d, $^{3}J_{HH}$ = 15.9 Hz, BnOC(O)CH=CH, 1H), 7.81 (d, $^{3}J_{HH}$ = 23.9 Hz, *(E)* CH=CP, 1H), 8.42 (s, $CH_{(5)}$, 1H), 8.44 (s, $CH_{(2)}$, 1H), **^{31}P NMR** (283 MHz, $CDCl_3$) δ: 16.09 – *(E)* CH=CP, 12.85 – *(Z)* CH=CP (E:Z)=(1:0.03), **^{13}C NMR** (176 MHz, $CDCl_3$) δ: 16.42 (d, $^{4}J_{PC}$ = 6.9 Hz, CH_3CH_2OP, 2C), 28.15 (s, $C(CH_3)_3$, 3C), 62.63 (m, CH_3CH_2OP, 2C), 66.78 (s, $PhCH_2O$, 1C), 82.96 (s, $C(CH_3)_3$, 1C), 118.81 (s, $CH_{(8)}$, 1C), 119,87 (d, $^{1}J_{PC}$ = 181.8 Hz, CP, 1C), 119.94 (s, BnOC(O)CH=CH, 1C), 121.14 (d, $^{3}J_{PC}$ = 24.8 Hz, $C_{(3)}$, 1C), 122.46 (s, $C_{(6)}$, 1C), 124.31 (s, $CH_{(7)}$, 1C), 125.18 (s, $CH_{(5)}$, 1C), 128.76 – 128.46, (Ph, 5C), 131.64 (d, $^{2}J_{PC}$ = 10.1 Hz, CH=CP, 1C), 135.86 (s, Ph – C_{IV}, 1C), 140.17 (s, BnOC(O)CH=CH, 1C), 141.09 (s, $CH_{(2)}$, 1C), 147.35 (s, C_9, 1C), 164.95 (d, $^{2}J_{PC}$ = 10.8 Hz, CO_2tBu, 1C), 165.96 (s, BnOC(O)CH=CH, 1C).

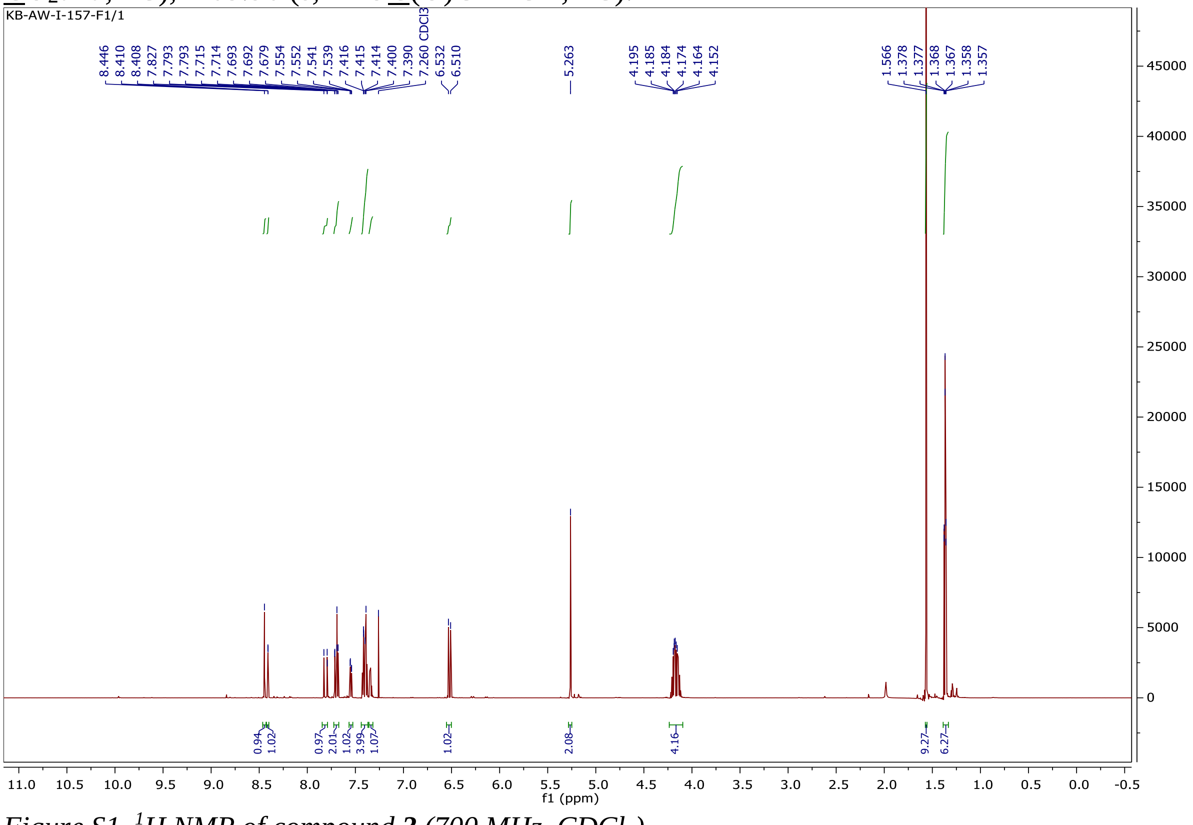

Figure S1. *^{1}H NMR of compound* ***3*** *(700 MHz, $CDCl_3$).*

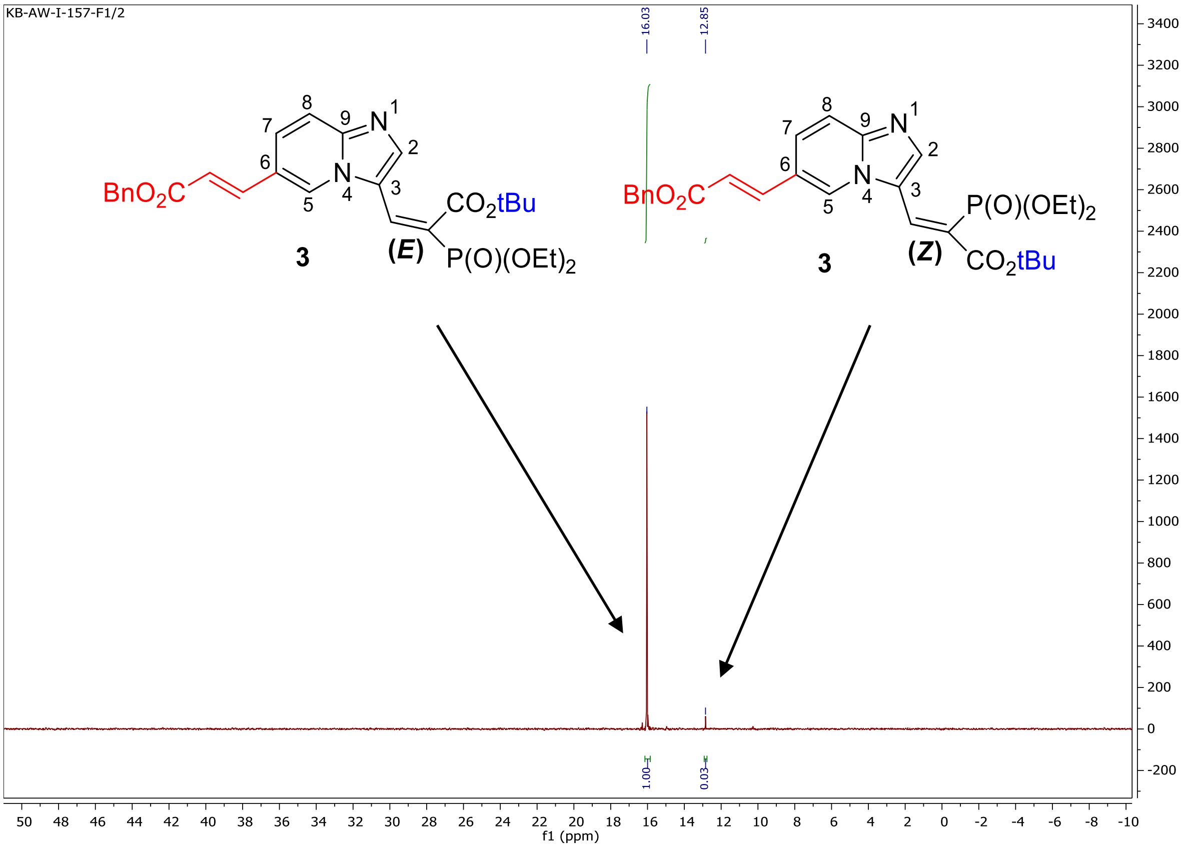

*Figure S2. ^{31}P NMR of compound **3** (ratio E/Z 100/3) (283 MHz, $CDCl_3$).*

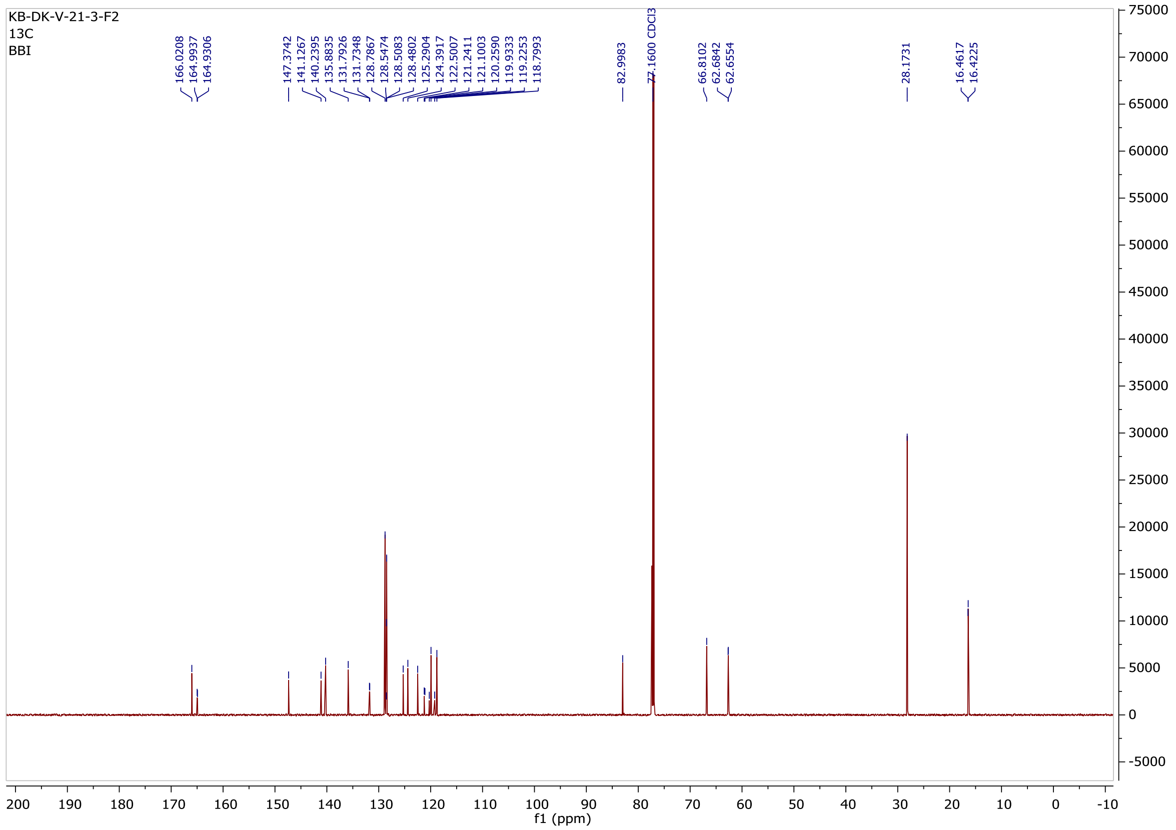

*Figure S3. ^{13}C NMR of compound **3** (176 MHz, $CDCl_3$)*

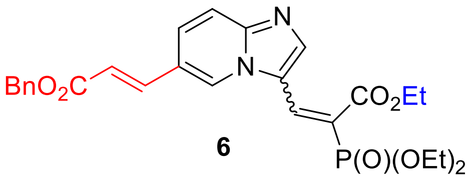

6

Ethyl 3-(6-((*E*)-3-(benzyloxy)-3-oxoprop-1-en-1-yl)imidazo[1,2-*a*]pyridin-3-yl)-2-(diethoxyphosphoryl)acrylate (6). Yield 55%

HRMS m/z: calculated 513.1785 ($C_{26}H_{29}N_2O_7P$ + H)$^+$, found 513.1797 ($C_{26}H_{29}N_2O_7P$ + H)$^+$, **^{1}H NMR** (700 MHz, $CDCl_3$) δ: 1.31-1.43 (m, (C<u>H</u>$_3$CH$_2$O)$_2$P, C<u>H</u>$_3$CH$_2$OC, 9H), 4.07 – 4.30 (m, (CH$_3$C<u>H</u>$_2$O)$_2$P, 4H), 4.37 (q, $^3J_{HH}$ =7.1 Hz, CH$_3$C<u>H$_2$</u>OC, 2H), 5.27 (s, PhC<u>H</u>$_2$O, 2H), 6.54 (d, $^3J_{HH}$ = 15.9 Hz, BnOC(O)C<u>H</u>=CH, 1H), 7.30 – 7.47 (m, <u>Ph</u>CH$_2$O, 5H), 7.58 (dd, $^3J_{HH}$ = 9.4 Hz, $^4J_{HH}$ = 1.6 Hz, C<u>H</u>$_{(7)}$, 1H), 7.65-7.78 (m, C<u>H</u>$_{(8)}$, BnOC(O)CH=C<u>H</u>, 2H), 7.97 (d, $^3J_{HH}$ = 23.8 Hz, *(E)* C<u>H</u>=CP, 1H), 8.47 (s, C<u>H</u>$_{(5)}$, 1H), 8.50 (s, C<u>H</u>$_{(2)}$, 1H), **^{31}P NMR** (283 MHz, $CDCl_3$) δ: 16.19 – *(E)* CH=CP, 12.83 – *(E)* CH=CP, (E:Z)=(1:0.03), **^{13}C NMR** (176 MHz, $CDCl_3$) δ: 14.15 (s, <u>C</u>H$_3$CH$_2$OC, 1C), 16.36 (d, $^4J_{PC}$ = 6.5 Hz, <u>C</u>H$_3$CH$_2$OP, 2C), 61.71 (s, CH$_3$<u>C</u>H$_2$OC, 1C), 62.81 (d, $^3J_{PC}$ = 5.2 Hz, (CH$_3$<u>C</u>H$_2$O)$_2$P, 2C), 66.71 (s, Ph<u>C</u>H$_2$O, 1C), 117.28 (d, $^1J_{PC}$ = 183.7 Hz, <u>C</u>P, 1C), 118.72 (s, <u>C</u>H$_{(8)}$, 1C), 119.97 (s, BnOC(O)<u>C</u>H=CH, 1C), 121.14 (d, $^3J_{PC}$ = 24.3 Hz, <u>C</u>$_{(3)}$, 1C), 122.58 (s, <u>C</u>$_{(6)}$, 1C), 124.68 (s, <u>C</u>H$_{(7)}$, 1C), 125.23 (s, <u>C</u>H$_{(5)}$, 1C), 128.37, 128.40, 128.68, (Ph, 5C), 133.30 (d, $^2J_{PC}$ = 10.0 Hz, <u>C</u>H=CP, 1C), 135.78 (s, Ph – C$_{IV}$, 1C), 140.06 (s, BnOC(O)CH=<u>C</u>H, 1C), 141.80 (s, <u>C</u>H$_{(2)}$, 1C), 147.51 (s, C$_9$, 1C), 165.56 (d, $^2J_{PC}$ = 11.5 Hz, <u>C</u>O$_2$tBu, 1C), 165.88 (s, BnO<u>C</u>(O)CH=CH, 1C).

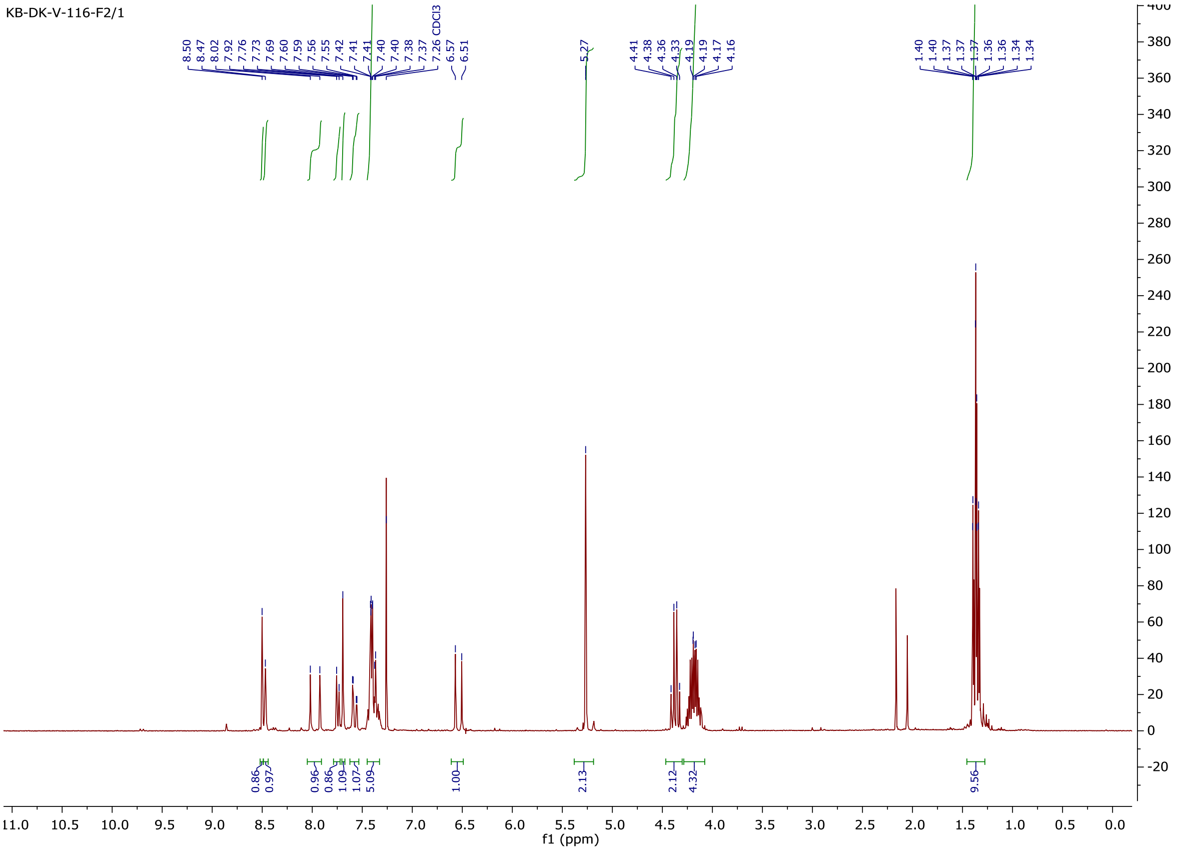

*Figure S4. ^{1}H NMR of compound **6** (700 MHz, $CDCl_3$)*

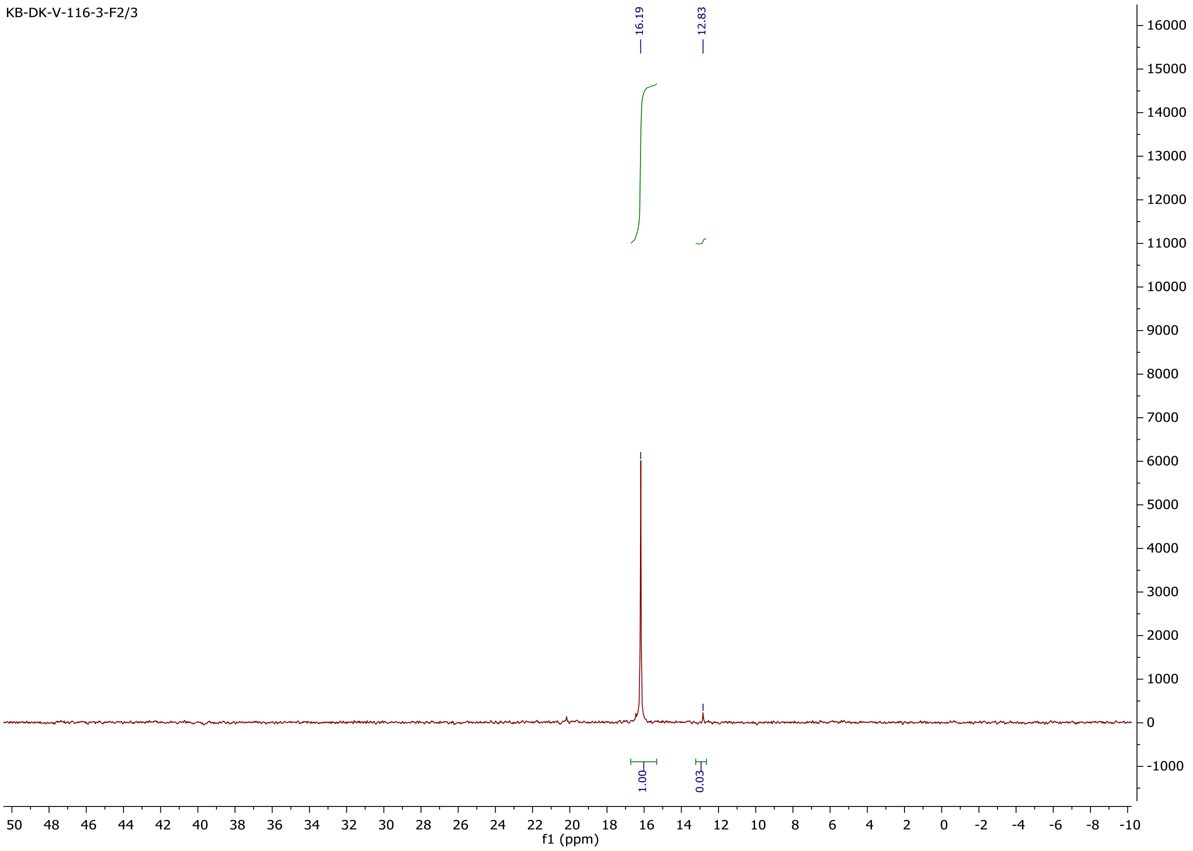

*Figure S5. ^{31}P NMR of compound **6** (ratio E/Z 100/3) (283 MHz, $CDCl_3$)*

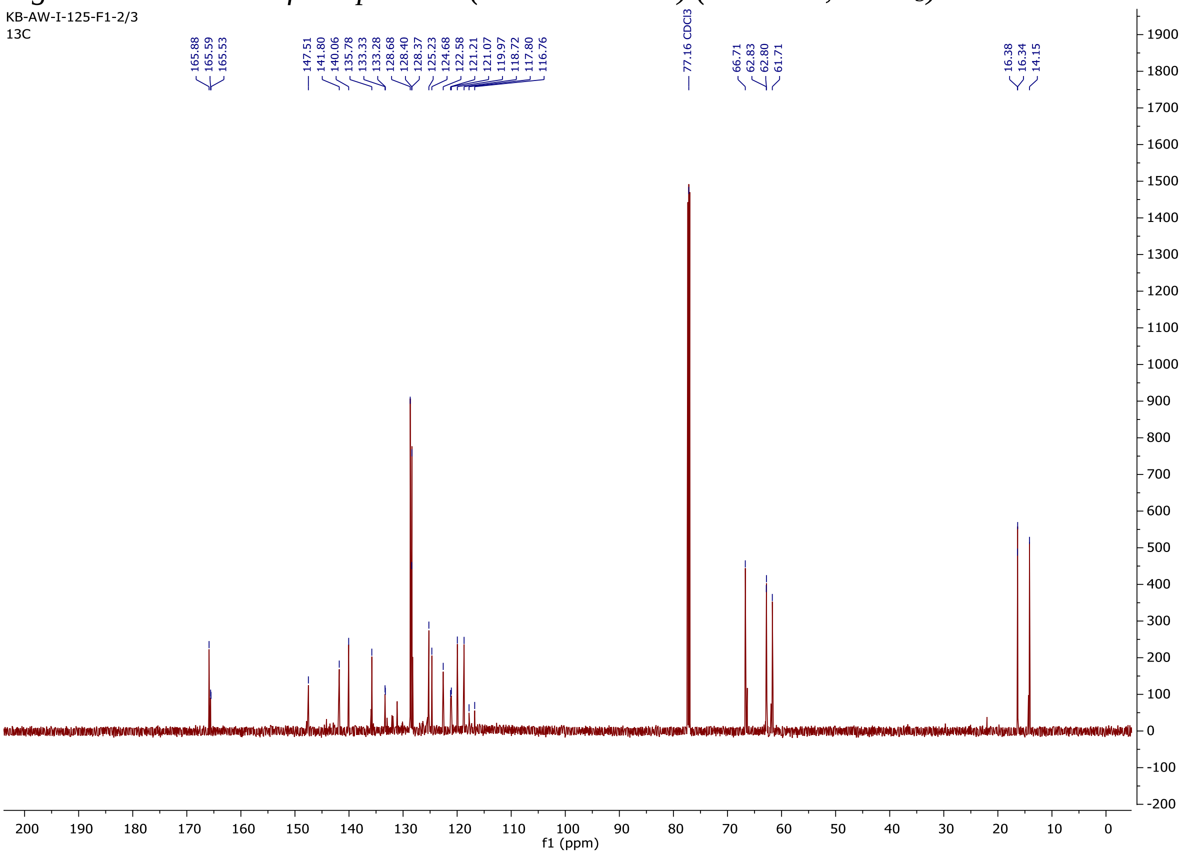

*Figure S6. ^{13}C NMR of compound **6** (176 MHz, $CDCl_3$)*

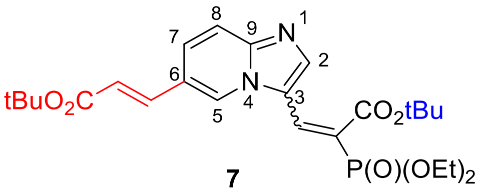

7

***tert*-Butyl 3-(6-((*E*)-3-(*tert*-butoxy)-3-oxoprop-1-en-1-yl)imidazo[1,2-*a*]pyridin-3-yl)-2-(diethoxyphosphoryl)acrylate (7).** Yield 68%.

HRMS m/z: calculated 507.2255 $(C_{25}H_{35}N_2O_7P + H)^+$, found 507.2267 $(C_{25}H_{35}N_2O_7P + H)^+$, **^{1}H NMR** (700 MHz, $CDCl_3$) δ: 1.35 (t, $^3J_{HH}$ = 7.1 Hz, C<u>H</u>$_3$CH$_2$OP, 6H), 1.52 (s, C(C<u>H</u>$_3$)$_3$, 9H), 1.55 (s, C(C<u>H</u>$_3$)$_3$, 9H), 3.91 – 4.37 (m, CH$_3$C<u>H</u>$_2$OP, 4H), 6.39 (d, $^3J_{HH}$ = 15.9 Hz, *t*-BuOC(O)C<u>H</u>=CH, 1H), 7.53 (dd, $^3J_{HH}$ = 9.3 Hz, $^4J_{HH}$ = 1.6 Hz, C<u>H</u>$_{(7)}$, 1H), 7.54 (d, $^3J_{HH}$ = 15.9 Hz *t*-BuOC(O)CH=C<u>H,</u> 1H), 7.66 (d, $^3J_{HH}$ = 9.3 Hz, $^5J_{HH}$ = 0,7 Hz, C<u>H</u>$_{(8)}$, 1H), 7.82 (d, $^3J_{HH}$ = 24.0 Hz, C<u>H</u>=CP, 1H), 8.42 (bs, C<u>H</u>$_{(5)}$, 1H), 8.43 (s, C<u>H</u>$_{(2)}$, 1H), **^{31}P NMR** (283 MHz, $CDCl_3$) δ: 16.16 – *(E)* CH=CP, 12.95 – *(Z)* CH=CP (E:Z)=(1:0.06), **^{13}C NMR** (176 MHz, $CDCl_3$) δ: 16.42 (d, $^3J_{PC}$ = 6.9 Hz, <u>C</u>H$_3$CH$_2$OP, 2C), 28.15 (s, C(<u>C</u>H$_3$)$_3$, 3C), 28.28 (s, C(<u>C</u>H$_3$)$_3$, 3C), 62.61 (d, $^2J_{PC}$ = 5.1 Hz, CH$_3$<u>C</u>H$_2$OP, 2C), 81.15 (s, <u>C</u>(CH$_3$)$_3$, 1C), 82.91 (s, <u>C</u>(CH$_3$)$_3$, 1C), 118.65 (s, <u>C</u>H$_{(8)}$, 1C), 119.43 (d, $^1J_{PC}$ = 182.1 Hz, <u>C</u>P, 1C), 121.05 (d, $^3J_{PC}$ = 24.8 Hz, <u>C</u>$_{(3)}$, 1C), 122.35 (s, *t*-BuOC(O)<u>C</u>H=CH, 1C), 122.79 (s, <u>C</u>$_{(6)}$, 1C), 124.61 (s, <u>C</u>H$_{(7)}$, 1C), 124.68 (s, <u>C</u>H$_{(5)}$, 1C), 131.80 (d, $^2J_{PC}$ = 10.1 Hz, <u>C</u>H=CP, 1C), 138.51 (s, *t*-BuOC(O)CH=<u>C</u>H, 1C), 141.04 (s, <u>C</u>H$_{(2)}$, 1C), 147.33 (s, C$_{(9)}$, 1C), 164.96 (d, $^2J_{PC}$ = 11,2 Hz, <u>C</u>O$_2$*t*-Bu, 1C), 165.40 (s, *t*-BuO<u>C</u>(O)CH=CH, 1C).

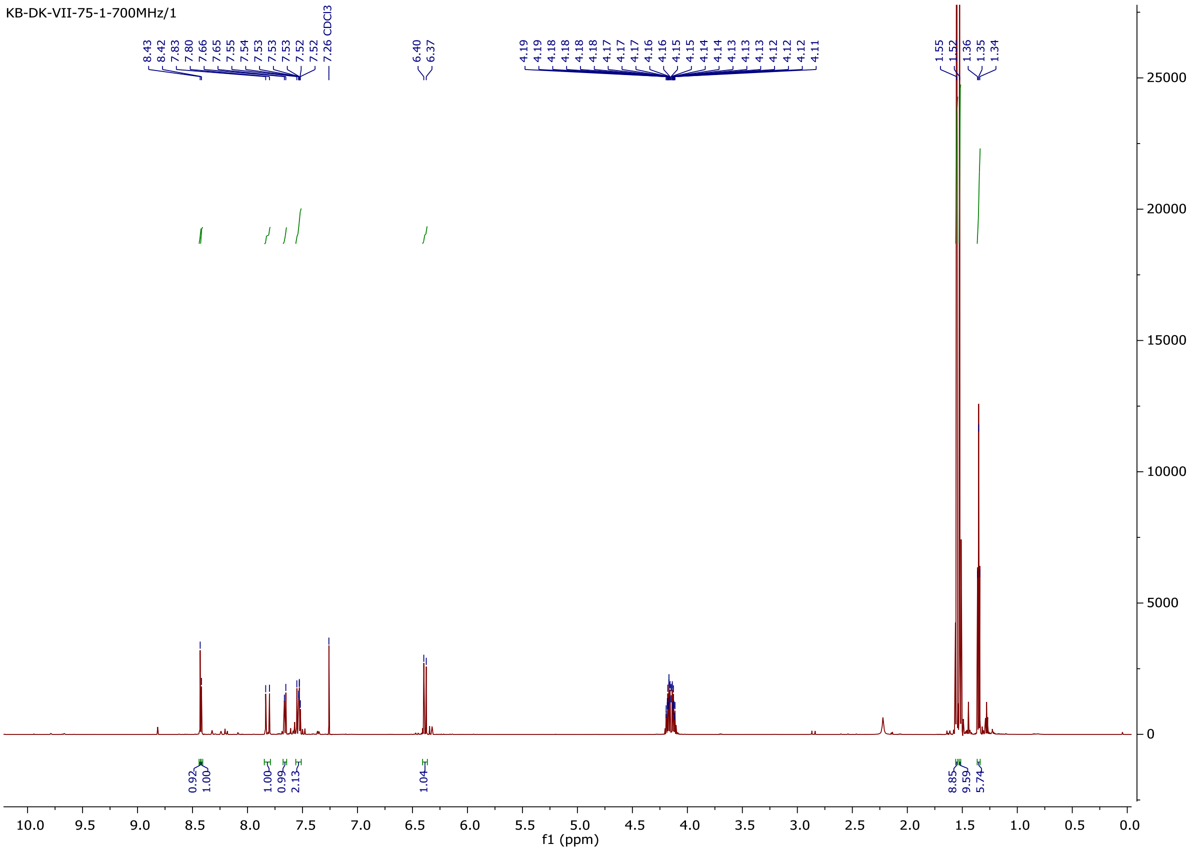

*Figure S7. ^{1}H NMR of compound **7** (700 MHz, $CDCl_3$).*

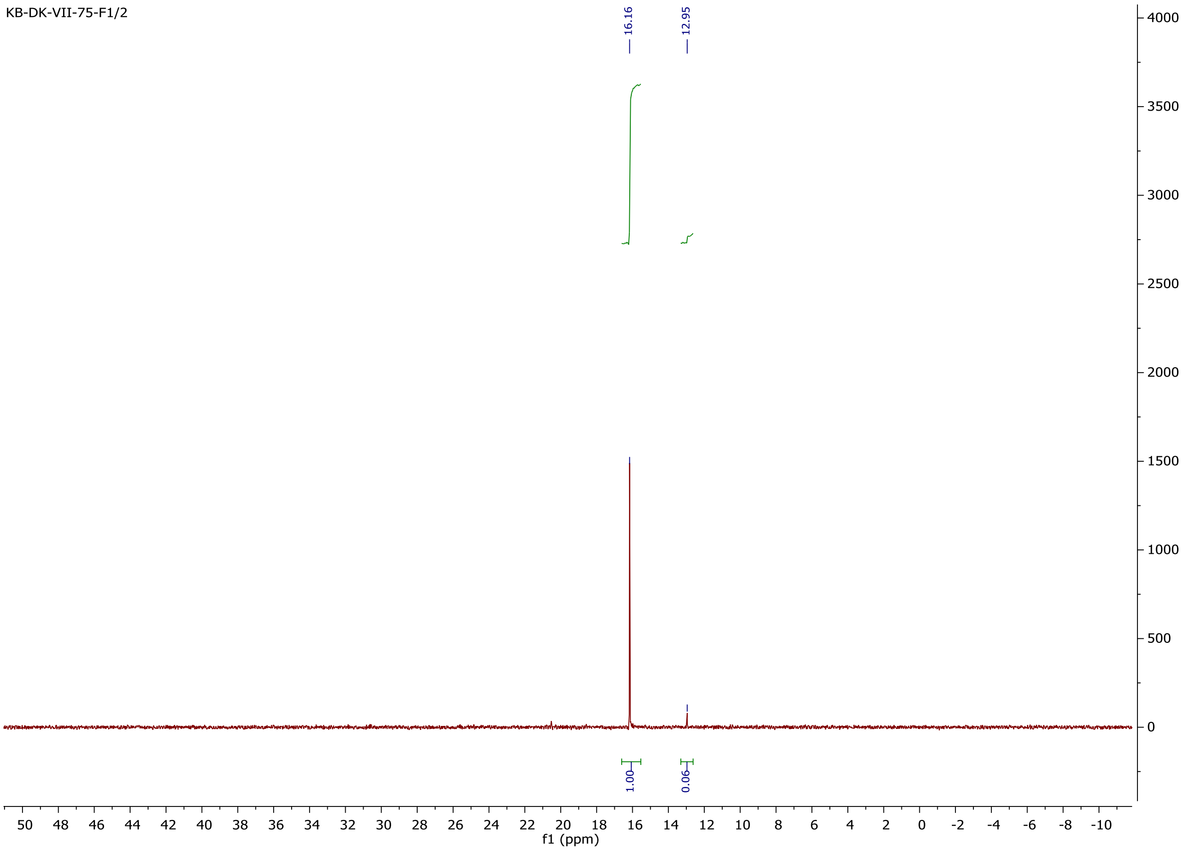

Figure S8. ^{31}*P NMR of compound* ***7*** *(ratio E/Z 100/6) (283 MHz,* $CDCl_3$*)*

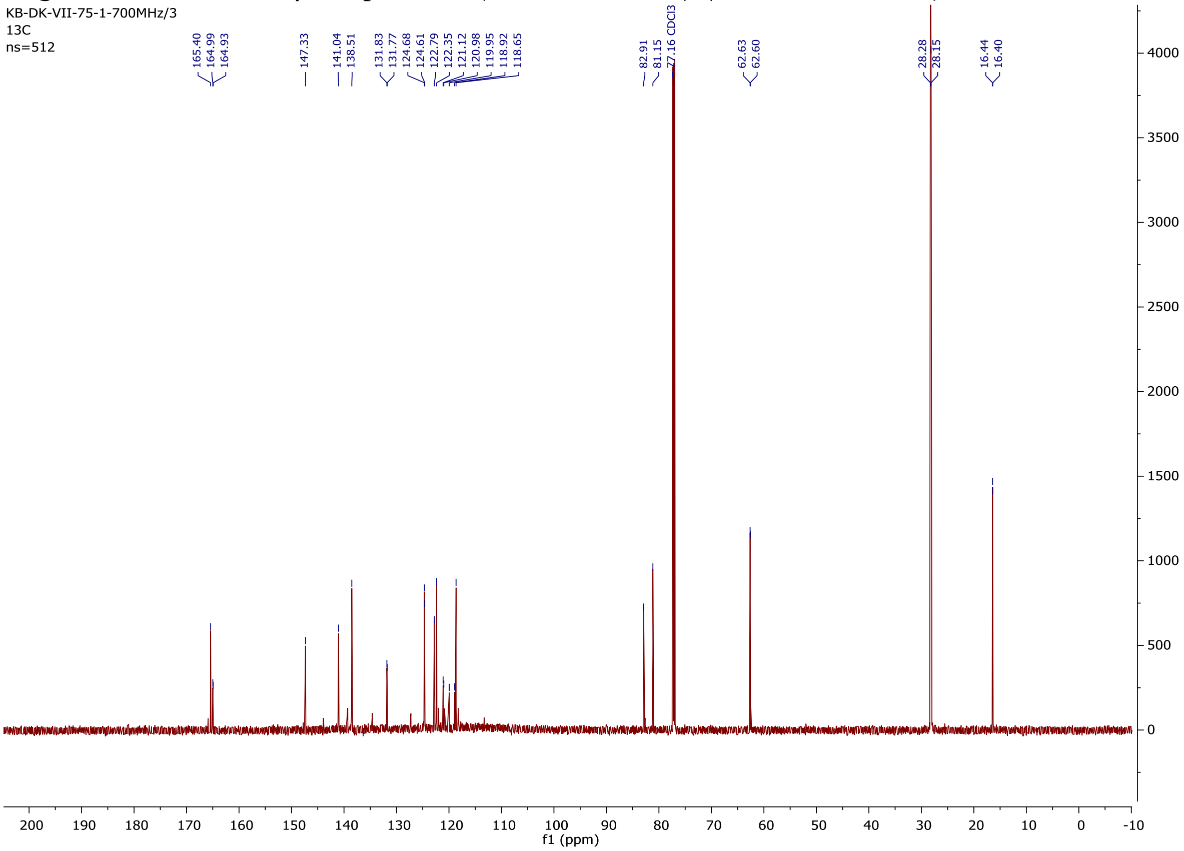

Figure S9. ^{13}*C NMR of compound* ***7*** *(176 MHz,* $CDCl_3$*)*

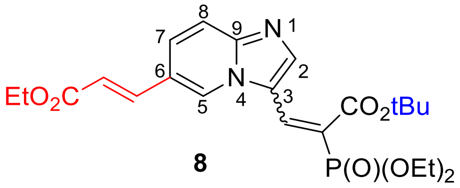

8

***tert*-Butyl 2-(diethoxyphosphoryl)-3-(6-((*E*)-3-ethoxy-3-oxoprop-1-en-1-yl)imidazo[1,2-*a*]pyridin-3-yl)acrylate (8).** Yield 74%.

HRMS m/z: calculated 479.1942 ($C_{23}H_{31}N_2O_7P$ + H)$^+$, found 479.1953 ($C_{23}H_{31}N_2O_7P$ + H)$^+$, **^{1}H NMR** (700 MHz, $CDCl_3$) δ: 1.31 (t, $^3J_{HH}$ = 7.1 Hz, C<u>H</u>$_3$CH$_2$OC=O, 3H), 1.34 (t, $^3J_{HH}$ = 7.1 Hz, C<u>H</u>$_3$CH$_2$OP, 6H), 1.54 (s, C(C<u>H</u>$_3$)$_3$, 9H), 4.03 – 4.21 (m, CH$_3$C<u>H</u>$_2$OP, 4H), 4.23 – 4.26 (q, $^3J_{HH}$ = 7.1 Hz, CH$_3$C<u>H</u>$_2$OC=O, 2H), 6.45 (d, $^3J_{HH}$ = 16.1 Hz, EtOC(O)C<u>H</u>=CH, 1H), 7.52 (dd, $^3J_{HH}$ = 9.4 Hz, $^4J_{HH}$ = 1.6 Hz, C<u>H</u>$_{(7)}$, 1H), 7.64 (d, $^3J_{HH}$ = 15.9 Hz, EtOC(O)CH=C<u>H</u>, 1H), 7.66 (d, $^3J_{HH}$ = 9.4 Hz, C<u>H</u>$_{(8)}$, 1H), 7.82 (d, $^3J_{HH}$ = 24.0 Hz, *(E)* C<u>H</u>=CP, 1H), 8.42 (s, C<u>H</u>$_{(2)}$, 1H), 8.48 (bs, C<u>H</u>$_{(5)}$, 1H), **^{31}P NMR** (283 MHz, $CDCl_3$) δ: 16.26 – *(E)* CH=CP, 12.86 – *(Z)* CH=CP (E:Z)=(1:0.11), **^{13}C NMR** (176 MHz, $CDCl_3$) δ: 14.28 (s, <u>C</u>H$_3$CH$_2$OC=O, 1C), 16.33 (d, $^4J_{PC}$ = 6.9 Hz, <u>C</u>H$_3$CH$_2$OP, 2C), 28.06 (s, C(<u>C</u>H$_3$)$_3$, 3C), 60.81 (s, CH$_3$<u>C</u>H$_2$OC=O, 1C), 62.53 (d, $^3J_{CP}$ = 5.2 Hz, CH$_3$<u>C</u>H$_2$OP, 2C), 82.83 (s, <u>C</u>(CH$_3$)$_3$, 1C), 118.64 (s, <u>C</u>H$_{(8)}$, 1C), 119,54 (d, $^1J_{PC}$ = 181,5 Hz, <u>C</u>P, 1C), 120.18 (s, EtOC(O)<u>C</u>H=CH, 1C), 121.03 (d, $^3J_{PC}$ = 24.8 Hz, <u>C</u>$_{(3)}$, 1C), 122.48 (s, <u>C</u>$_{(6)}$, 1C), 124.36 (s, <u>C</u>H$_{(7)}$, 1C), 125.04 (s, <u>C</u>H$_{(5)}$, 1C), 131.72 (d, $^2J_{PC}$ = 10.1 Hz, <u>C</u>H=CP, 1C), 139.59 (s, EtOC(O)CH=<u>C</u>H, 1C), 141.00 (s, <u>C</u>H$_{(2)}$, 1C), 147.26 (s, C$_{(9)}$, 1C), 164.85 (d, $^2J_{PC}$ = 10.9 Hz, <u>C</u>O$_2$*t*-Bu, 1C), 166.10 (s, EtO<u>C</u>(O)CH=CH, 1C).

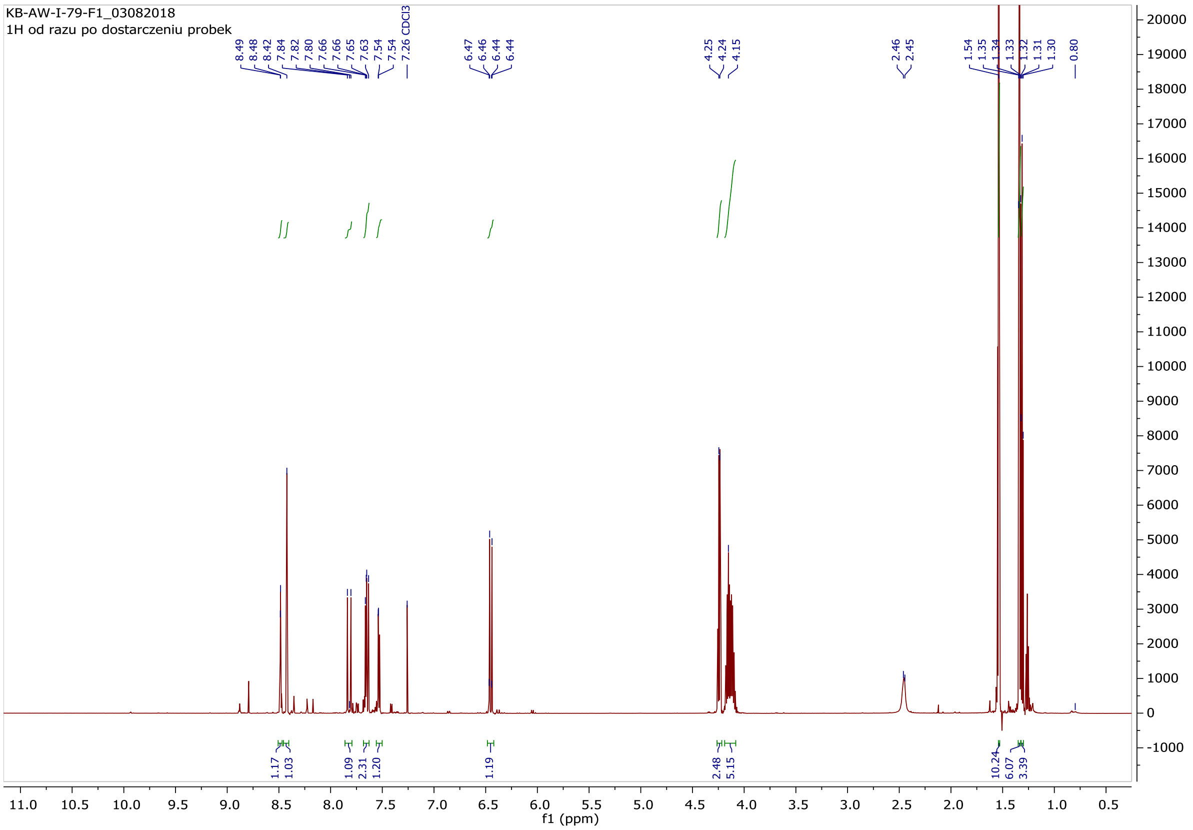

*Figure S10. ^{1}H NMR of compound **8** (700 MHz, $CDCl_3$)*

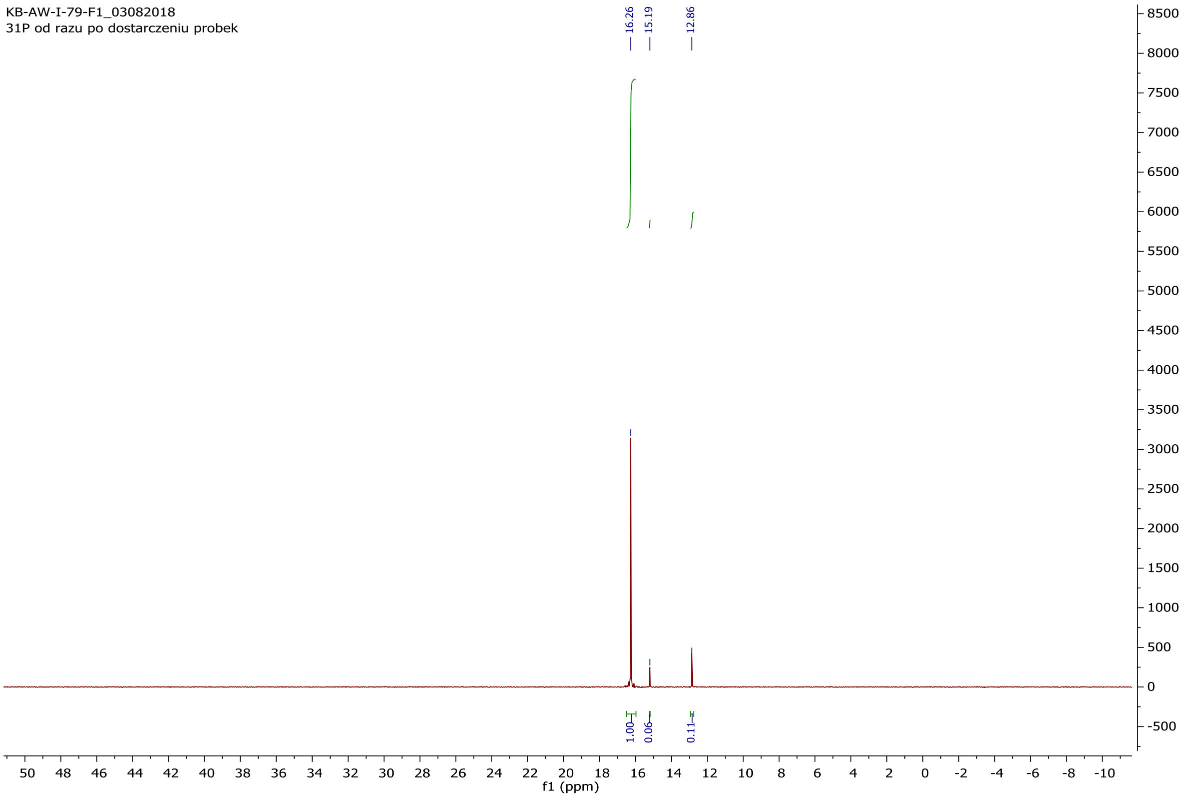

*Figure S11. ^{31}P NMR of compound **8** (ratio E/Z 100/11) (283 MHz, $CDCl_3$)*

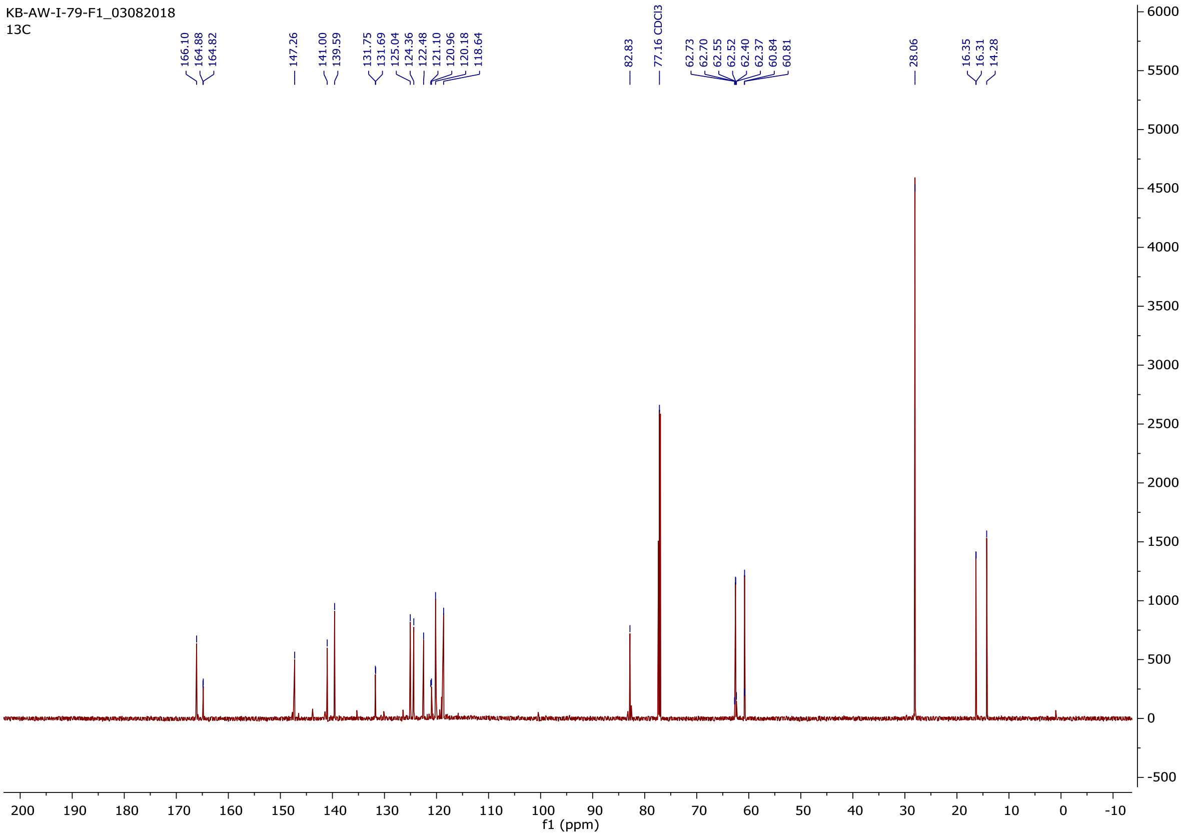

*Figure S12. ^{13}C NMR of compound **8** (176 MHz, $CDCl_3$)*

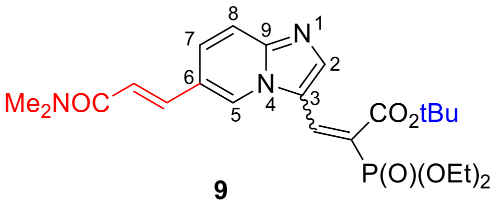

9

***tert*-Butyl 2-(diethoxyphosphoryl)-3-(6-((*E*)-3-(dimethylamino)-3-oxoprop-1-en-1-yl)imidazo[1,2-*a*]pyridin-3-yl)acrylate (9).** Yield 79%.

HRMS m/z: calculated 478.2101 ($C_{23}H_{32}N_3O_6P$ + H)$^+$, found 478.2107 ($C_{23}H_{32}N_3O_6P$ + H)$^+$, **^{1}H NMR** (700 MHz, $CDCl_3$) δ: 1.34 (t, $^3J_{HH}$ = 7.1 Hz, C<u>H</u>$_3$CH$_2$OP, 6H), 1.54 (s, C(C<u>H</u>$_3$)$_3$, 9H), 3.05 (s, NC<u>H</u>$_3$, 3H), 3.17 (s, NC<u>H</u>$_3$, 3H), 4.08 – 4.20 (m, CH$_3$C<u>H</u>$_2$OP, 4H), 6.94 (d, $^3J_{HH}$ = 15.4 Hz, (CH$_3$)$_2$NC(O)C<u>H</u>=CH, 1H), 7.54 (dd, $^3J_{HH}$ = 9.3 Hz, $^4J_{HH}$ = 1.6 Hz, C<u>H</u>$_{(7)}$, 1H), 7.62 (d, $^3J_{HH}$ = 15.4 Hz, (CH$_3$)$_2$NC(O)CH=C<u>H</u>, 1H), 7.65 (d, $^3J_{HH}$ = 9.3 Hz, C<u>H</u>$_{(8)}$, 1H), 7.81 (d, $^3J_{HH}$ = 23.9 Hz, *(E)* C<u>H</u>=CP, 1H), 8.41 (s, C<u>H</u>$_{(2)}$, 1H), 8.48 (bs, C<u>H</u>$_{(5)}$, 1H), **^{31}P NMR** (283 MHz, $CDCl_3$) δ: 16.58 – *(E)* CH=CP, 13.35 – *(Z)* CH=CP, (E:Z)=(1:0.15), **^{13}C NMR** (176 MHz, $CDCl_3$) δ: 16.39 (d, $^4J_{PC}$ = 6.8 Hz, <u>C</u>H$_3$CH$_2$OP, 2C), 28.12 (s, C(<u>C</u>H$_3$)$_3$, 3C), 36.08 (s, N<u>C</u>H$_3$, 1C), 37.58 (s, N<u>C</u>H$_3$, 1C), 62.61 (d, $^3J_{CP}$ = 5.2 Hz, CH$_3$<u>C</u>H$_2$OP, 2C), 82.89 (s, <u>C</u>(CH$_3$)$_3$, 1C), 118.43 (s, <u>C</u>H$_{(8)}$, 1C), 119.37 (s, (CH$_3$)$_2$NC (O)<u>C</u>H=CH, 1C), 119.41 (d, $^1J_{PC}$ = 182.6 Hz, <u>C</u>P, 1C), 121.04 (d, $^3J_{PC}$ = 24.9 Hz, <u>C</u>$_{(3)}$, 1C), 123.36 (s, <u>C</u>$_{(6)}$, 1C), 124.27 (s, <u>C</u>H$_{(5)}$, 1C), 125.14 (s, <u>C</u>H$_{(7)}$, 1C), 131.76 (d, $^2J_{PC}$ = 10.1 Hz, <u>C</u>H=CP, 1C), 137.62 (s, (CH$_3$)$_2$NC(O)CH=<u>C</u>H, 1C), 140.91 (s, <u>C</u>H$_{(2)}$, 1C), 147.25 (s, C$_{(9)}$, 1C), 165.00 (d, $^2J_{PC}$ = 11.1 Hz, <u>C</u>O$_2$*t*-Bu, 1C), 165.90 (s, <u>C</u>CH=CH, 1C).

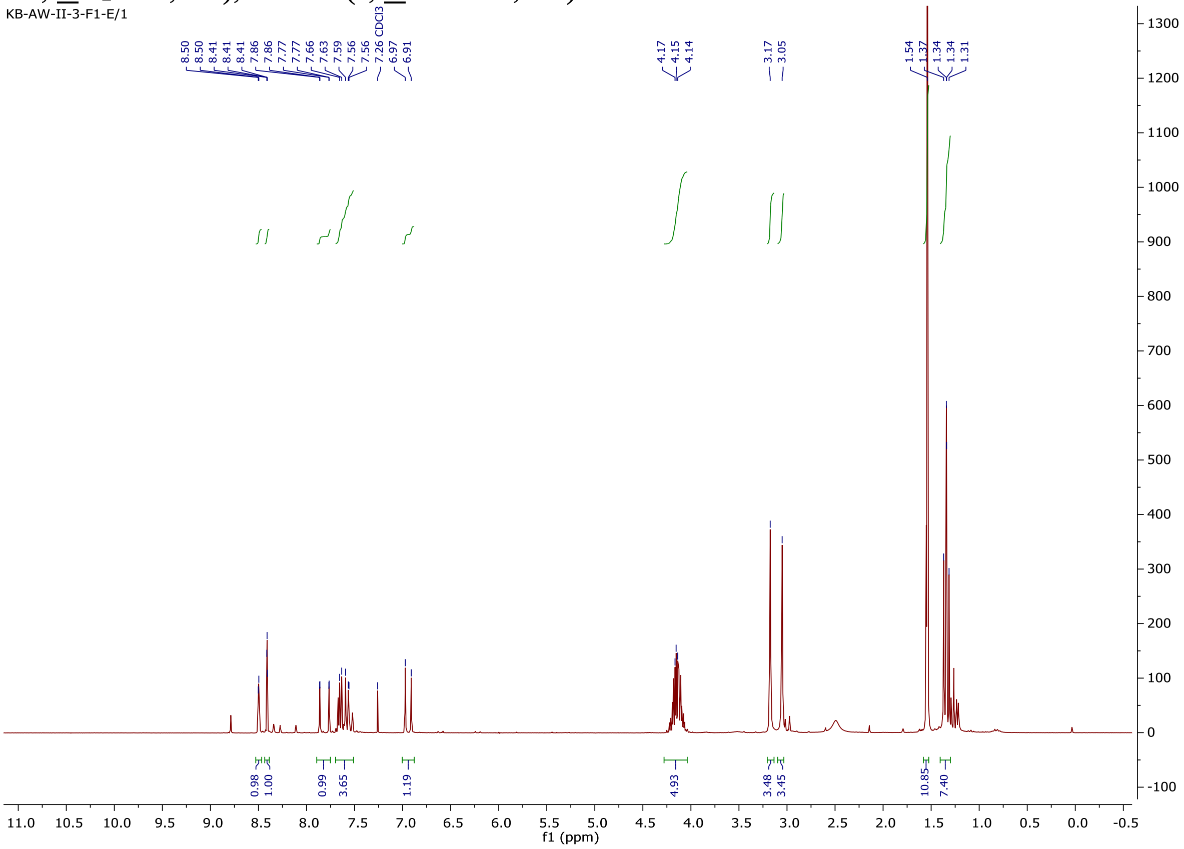

*Figure S13. ^{1}H NMR of compound **9** (700 MHz, $CDCl_3$)*

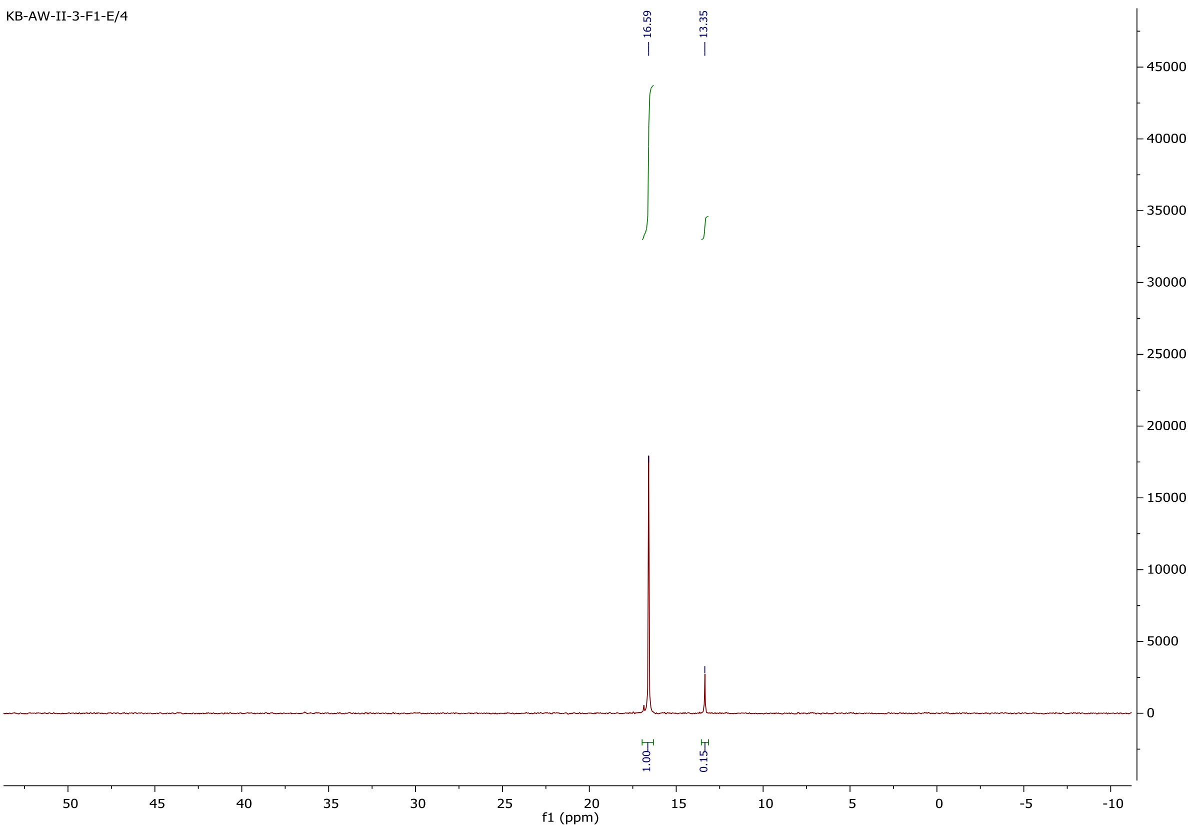

*Figure S14. ^{31}P NMR of compound **9** (ratio E/Z 100/15) (283 MHz, $CDCl_3$)*

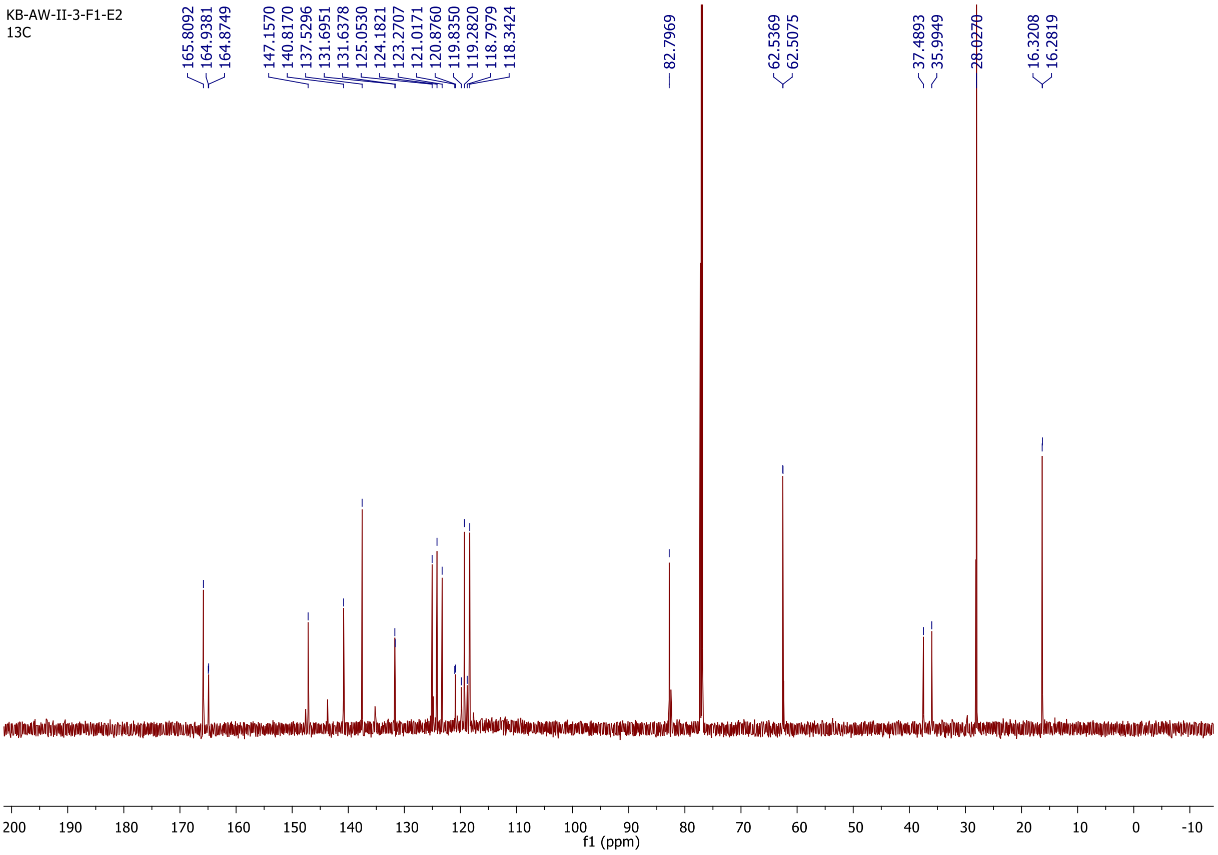

*Figure S15. ^{13}C NMR of compound **9** (176 MHz, $CDCl_3$)*

Other possible Heck products

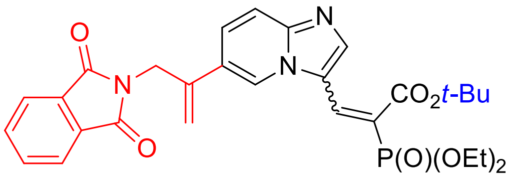

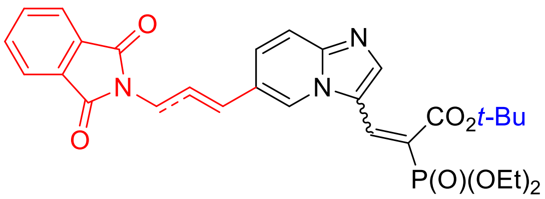

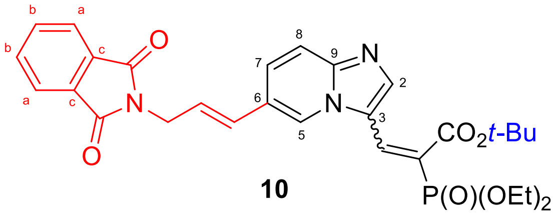

10

Main product

***tert*-Butyl 2-(diethoxyphosphoryl)-3-(6-((*E*)-3-(1,3-dioxoisoindolin-2-yl)prop-1-en-1-yl)imidazo[1,2-*a*]pyridin-3-yl)acrylate (10).** Yield 65%.

HRMS m/z: calculated 566.2051 $(C_{29}H_{32}N_3O_7P + H)^+$, found 566.2061 $(C_{29}H_{32}N_3O_7P + H)^+$, **^{1}H NMR** (700 MHz, $CDCl_3$) δ: 1.30 (t, $^3J_{HH}$ = 7.1Hz, $C\underline{H}_3CH_2OP$, 6H), 1.51 (s, $C(C\underline{H}_3)_3$, 9H), 4.00 – 4.19 (m, $CH_3C\underline{H}_2OP$, 4H), 4.43 (bd, $^3J_{HH}$ = 5.6 Hz, $PhtC\underline{H}_2CH{=}CH$, 2H), 6.28 (dt, $^3J_{HH}$ = 15.8 Hz, $^3J_{HH}$ = 5.9 Hz, $PhtCH_2C\underline{H}{=}CH$, 1H), 6.52 (d, $^3J_{HH}$ = 15.8 Hz, $PhtCH_2CH{=}C\underline{H}$, 1H), 7.43 (bd, $^3J_{HH}$ = 9.2 Hz, $C\underline{H}_{(7)}$, 1H), 7.55 (bd, $^3J_{HH}$ = 9.2 Hz, $C\underline{H}_{(8)}$, 1H), 7.68 (m, $C\underline{H}_{(b)}$, 2H), 7.73 (d, $^3J_{HH}$ = 24.1 Hz, $C\underline{H}{=}CP$, 1H), 7.81 (m, $C\underline{H}_{(a)}$, 2H), 8.16 (bs, $C\underline{H}_{(5)}$, 1H), 8.36 (s, $C\underline{H}_{(2)}$, 1H), **^{31}P NMR** (283 MHz, $CDCl_3$) δ: 16.69 – *(E)* CH=CP, 13.31 – *(Z)* CH=CP, (E:Z)=(1:0.04), **^{13}C NMR** (176 MHz, $CDCl_3$) δ: 16.30 (d, $^4J_{PC}$ = 6.5 Hz, $\underline{C}H_3CH_2OP$, 2C), 28.04 (s, $C(\underline{C}H_3)_3$, 3C), 39.25 ($Pht\underline{C}H_2$, 1C), 62.40 (d, $^3J_{PC}$ = 5.0 Hz, $CH_3\underline{C}H_2OP$, 2C), 82.61 (s, $\underline{C}(CH_3)_3$, 1C), 118.08 (s, $\underline{C}H_{(8)}$, 1C), 118,21 (d, $^1J_{PC}$ = 182.7 Hz, $\underline{C}P$, 1C), 120.66 (d, $^3J_{PC}$ = 25.1 Hz, $\underline{C}_{(3)}$, 1C), 122.11 (s, $\underline{C}H_{(5)}$, 1C), 123.49 (s, $C\underline{H}_{(a)}$, 2C), 124.11 (s, $\underline{C}_{(6)}$, 1C), 124.83 (s, $\underline{C}H_{(7)}$, 1C), 125.26 (s, $PhtCH_2\underline{C}H{=}CH$, 1C), 128.05 (s, $PhtCH_2CH{=}\underline{C}H$, 1C), 132.01 (s, $C\underline{H}_{(c)}$, 2C), 132.07 (d, $H\underline{C}{=}CP$, $^2J_{PC}$ = 9.8 Hz), 134.15 (s, $C\underline{H}_{(b)}$, 2C), 140.72 (s, $\underline{C}H_{(2)}$, 1C), 146.96 (s, $\underline{C}_{(9)}$, 1C), 164.98 (d, $^2J_{PC}$ = 11.4 Hz, $\underline{C}O_2t$-Bu, 1C), 167.84 (s, $O{=}\underline{C}N$, 2C).

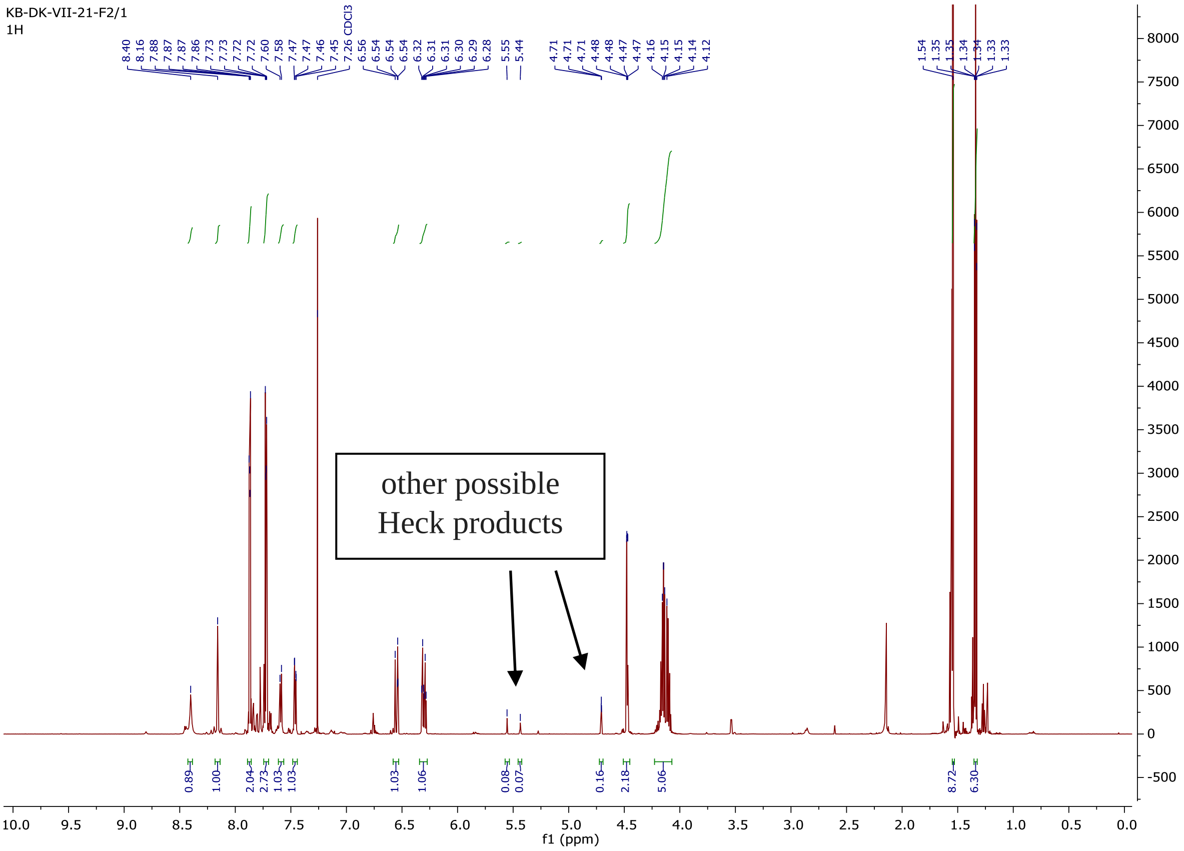

Figure S16. ^{1}*H NMR of compound* ***10*** *(700 MHz,* $CDCl_3$*)*

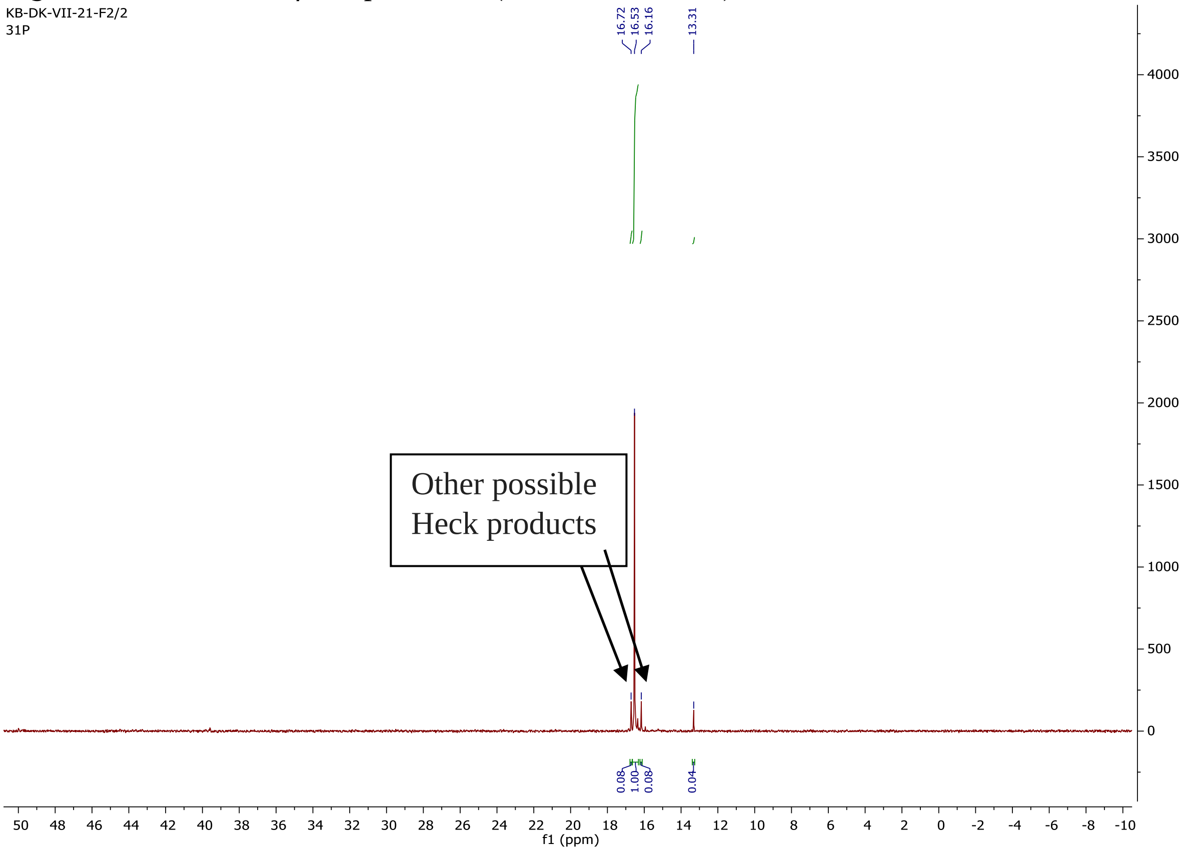

Figure S17. ^{31}*P NMR of compound* ***10*** *(ratio E/Z 100/4) (283 MHz,* $CDCl_3$*)*

^{31}P NMR of fraction after chromatographical purification showing the presence of 4 Heck products; differentiated by the geometry of pre-existing double bond in substrate.

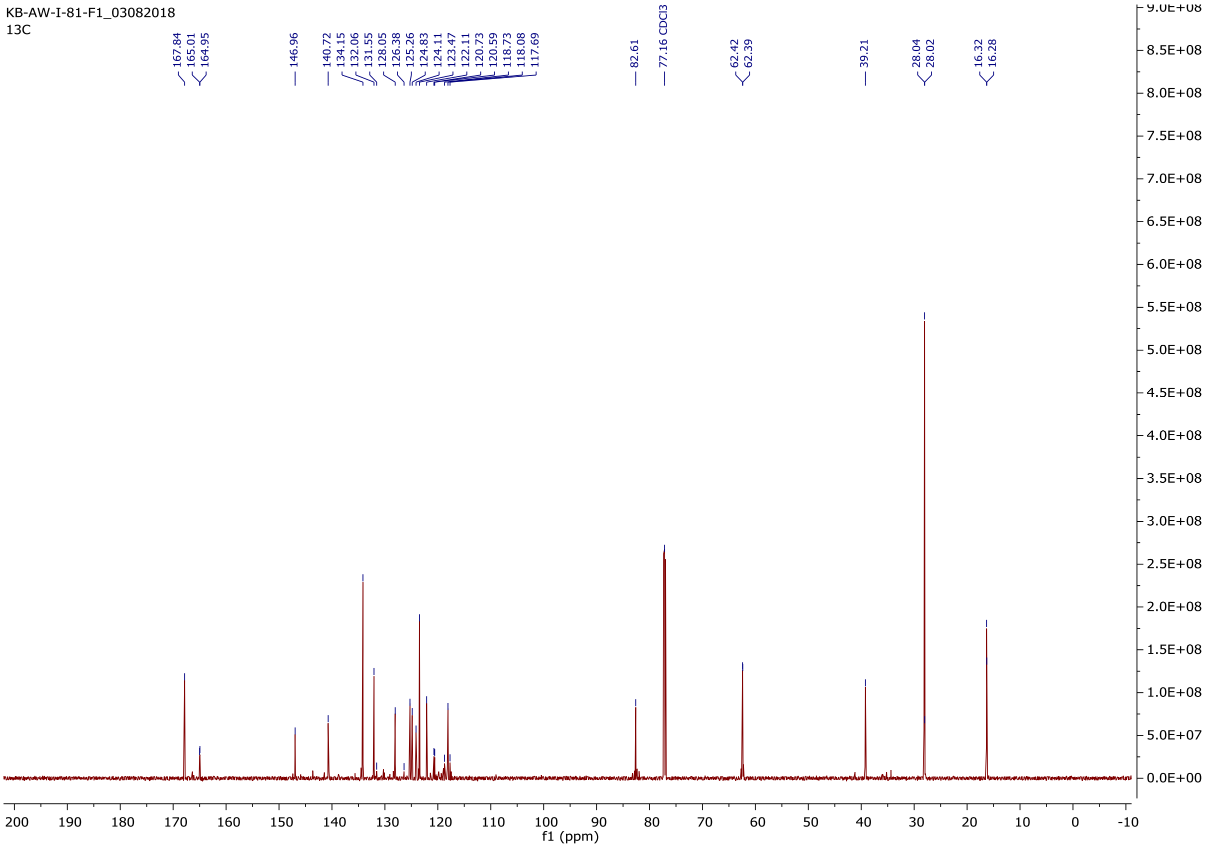

*Figure S18. ^{13}C NMR of compound **10** (176 MHz, $CDCl_3$)*

Composition of the reaction mixture

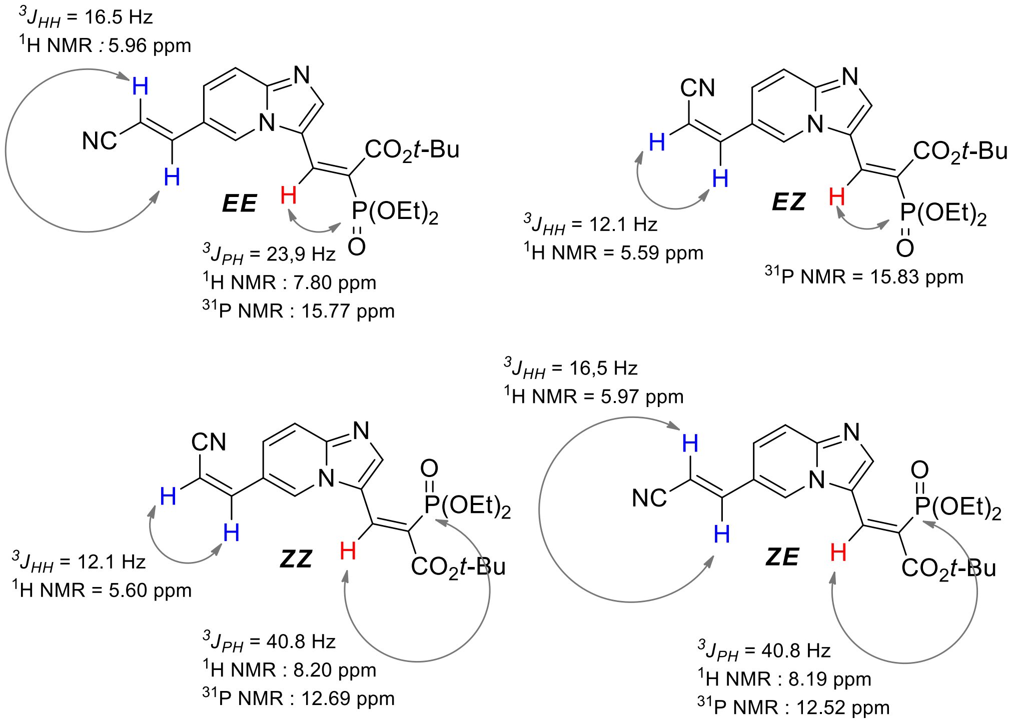

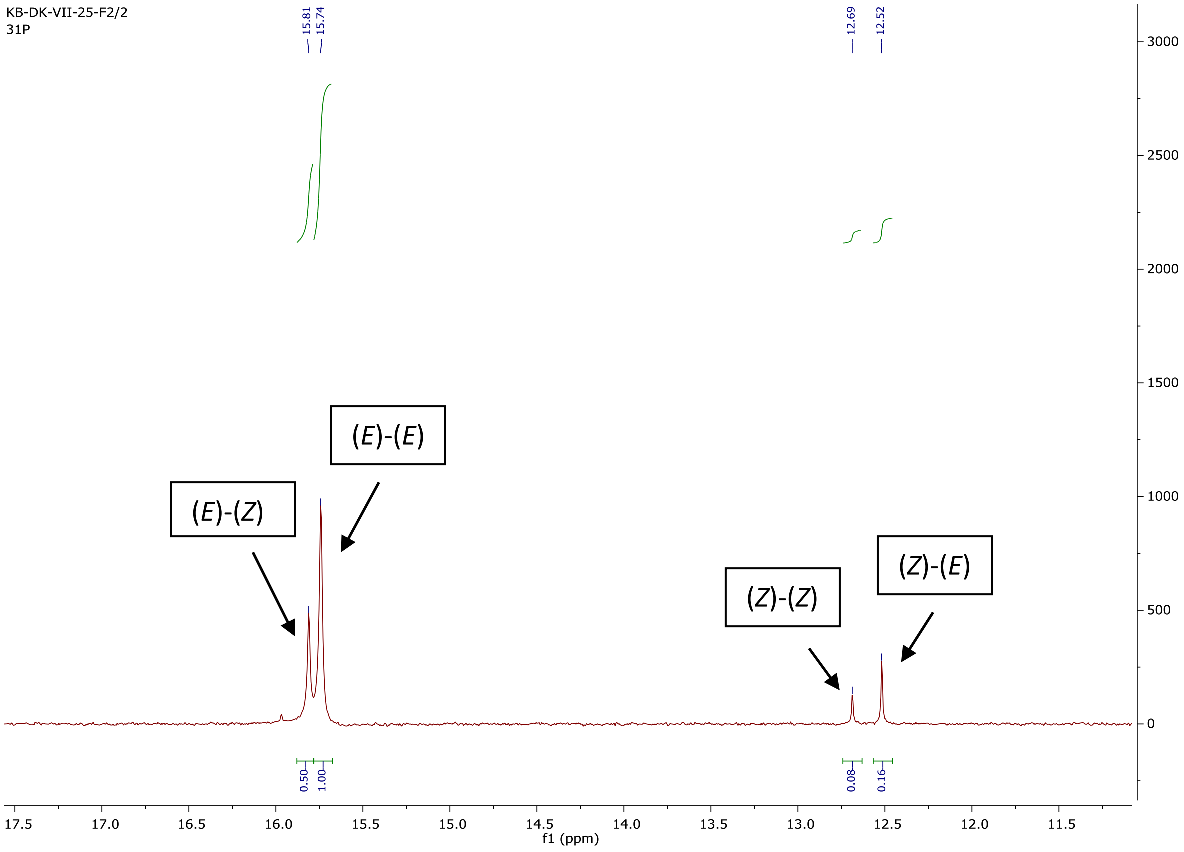

*Figure S19. Composition of the reaction mixture (compound **11**)*

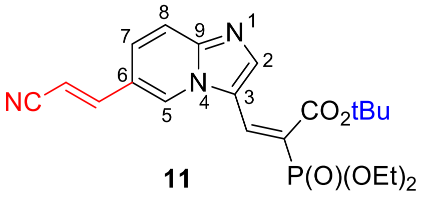

11

Main isomer

(*E*)-*tert*-Butyl 3-(6-((*E*)-2-cyanovinyl)imidazo[1,2-*a*]pyridin-3-yl)-2-(diethoxyphosphoryl)acrylate (11). Yield 50%.

HRMS m/z: calculated 432.1683 ($C_{21}H_{26}N_3O_5P$ + H)$^+$, found 432.1697 ($C_{21}H_{26}N_3O_5P$ + H)$^+$, **^{1}H NMR** (700 MHz, $CDCl_3$) δ: 1.34 – 1.40 (m, C<u>H</u>$_3$CH$_2$OP, 6H), 1.56 (s, C(C<u>H</u>$_3$)$_3$, 9H), 4.08 –4.23 (m, CH$_3$C<u>H</u>$_2$OP, 4H), 5.96 (d, $^3J_{HH}$ = 16.5 Hz, NCC<u>H</u>=CH, 1H), 7.39 (d, $^3J_{HH}$ = 16.5 Hz, NCCH=C<u>H</u>, 1H), 7.46 (dd, $^3J_{HH}$ = 9.3 Hz, $^4J_{HH}$ = 1.7 Hz, C<u>H</u>$_{(7)}$, 1H), 7.71 (d, $^3J_{HH}$ = 9.3 Hz, C<u>H</u>$_{(8)}$, 1H), 7.80 (d, $^3J_{HH}$ = 23.9 Hz, *(E)* C<u>H</u>=CP, 1H), 8.45 (bs, C<u>H</u>$_{(5)}$, 1H), 8.45 (s, C<u>H</u>$_{(2)}$, 1H), **^{31}P NMR** (283 MHz, $CDCl_3$) δ: 16.09 – *(E)* CH=CP, 12.87– *(Z)* CH=CP, (E:Z)=(1:0.12), **^{13}C NMR** (176 MHz, $CDCl_3$) δ: 16.43 (d, $^3J_{PC}$ = 6,9 Hz, <u>C</u>H$_3$CH$_2$OP, 2C), 28.16 (s, C(<u>C</u>H$_3$)$_3$, 3C), 62.73 (d, $^2J_{PC}$ = 5.1 Hz, CH$_3$<u>C</u>H$_2$OP, 2C), 83.14 (s, <u>C</u>(CH$_3$)$_3$, 1C), 98.36 (s, NC<u>C</u>H=CH, 1C), 117.41 (s, N<u>C</u>CH=CH, 1C), 119.19 (s, <u>C</u>H$_{(8)}$, 1C), 120.68 (d, $^1J_{PC}$ = 181.4 Hz, <u>C</u>P, 1C), 121.41 (d, $^3J_{PC}$ = 25.0 Hz, <u>C</u>$_{(3)}$, 1C), 121.71 (s, <u>C</u>$_{(6)}$, 1C), 122.89 (s, <u>C</u>H$_{(7)}$, 1C), 125.22 (s, <u>C</u>H$_{(5)}$, 1C), 131.35 (d, $^2J_{PC}$ = 10.1 Hz, <u>C</u>H=CP, 1C), 141.21 (s, <u>C</u>H$_{(2)}$, 1C), 145.70 (s, NCCH=<u>C</u>H, 1C), 147.25 (s, C$_9$, 1C), 164.86 (d, $^2J_{PC}$ = 10.8 Hz, <u>C</u>O$_2$*t*-Bu, 1C).

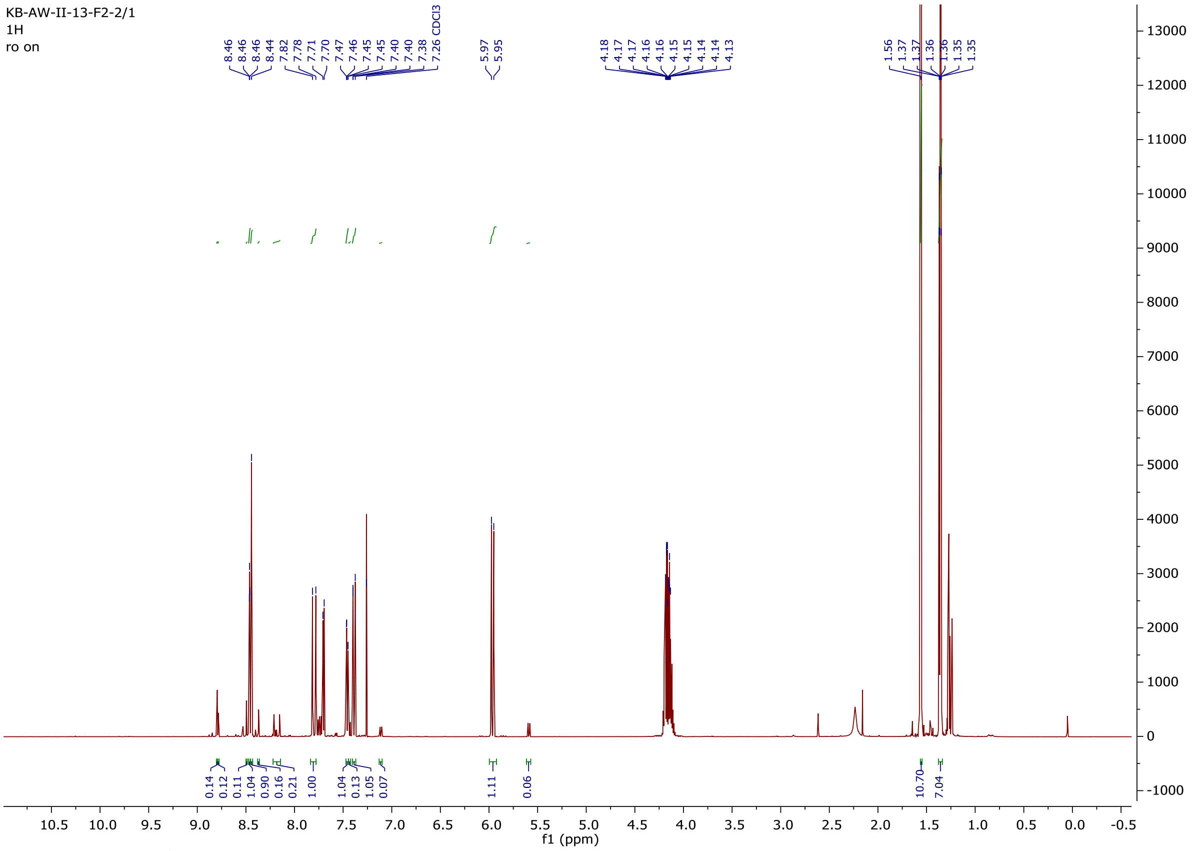

Figure S20. ^{1}*H NMR of compound* ***11*** *(700 MHz, $CDCl_3$)*

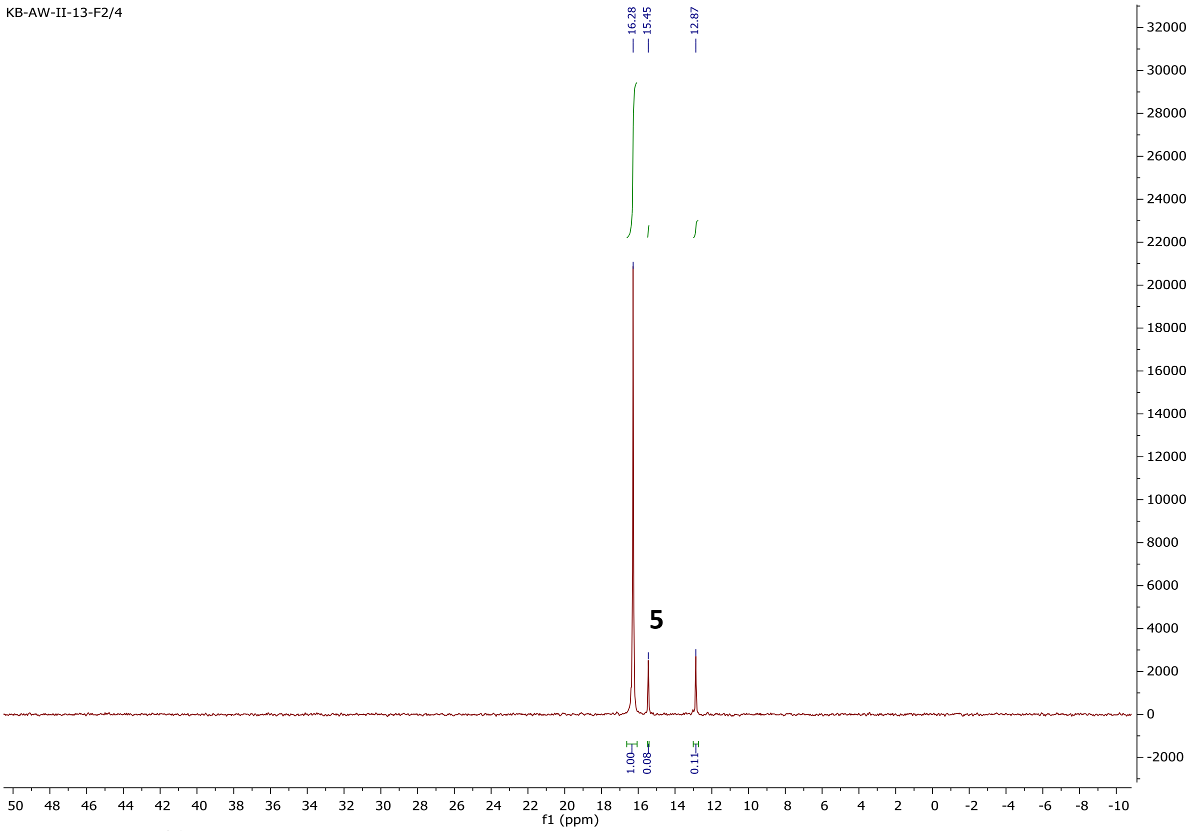

Figure S21. ^{31}*P NMR of compound* ***11*** *(283 MHz, $CDCl_3$)*

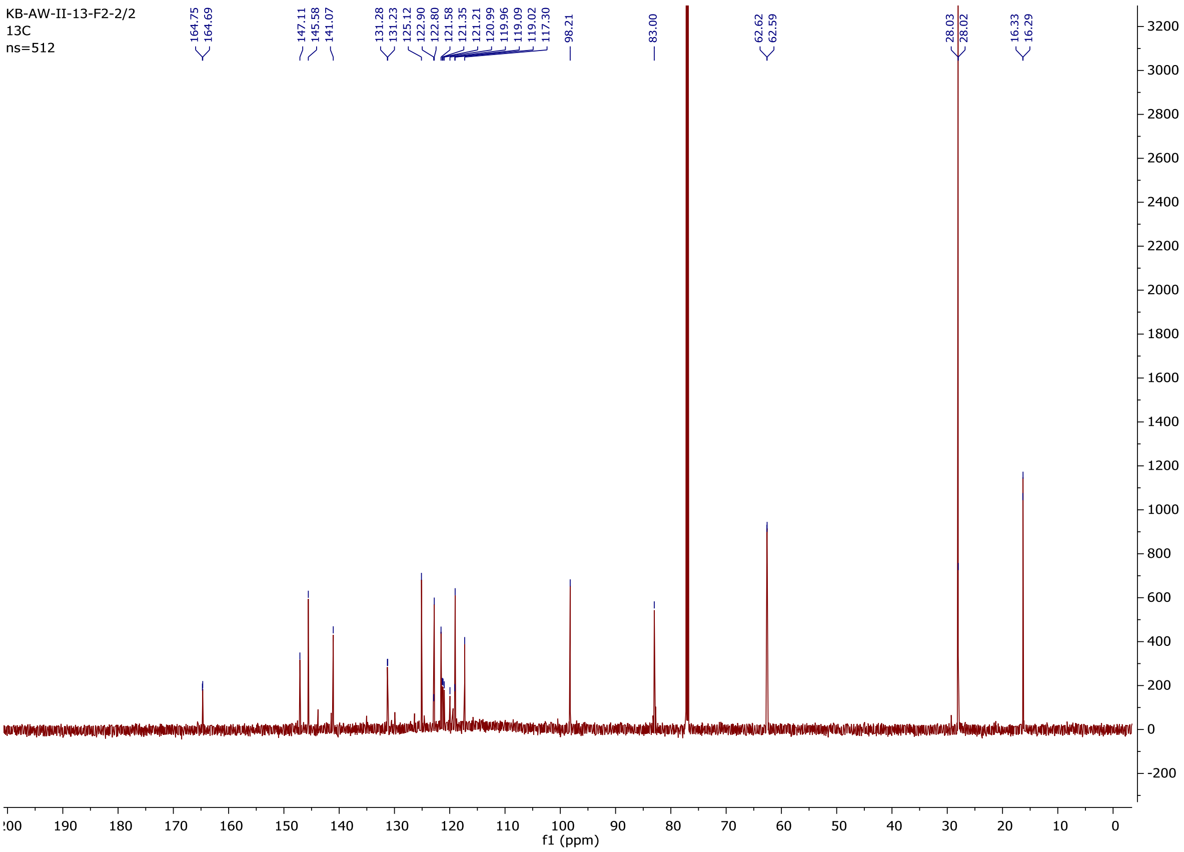

Figure S22. ^{13}*C NMR of compound* ***11*** *(176 MHz,* $CDCl_3$*)*

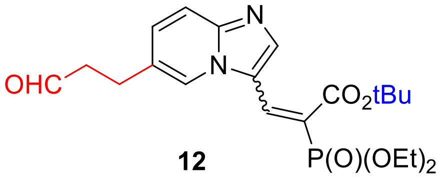

12

***tert*-Butyl 2-(diethoxyphosphoryl)-3-(6-(3-oxopropyl)imidazo[1,2-*a*]pyridin-3-yl)acrylate (12)**. Yield 67%.

HRMS m/z: calculated 437.1836 ($C_{21}H_{29}N_2O_6P$ + H)$^+$, found 437.1840 ($C_{21}H_{29}N_2O_6P$ + H)$^+$, ^{1}**H NMR** (700 MHz, $CDCl_3$) δ: 1.30 (t, $^3J_{HH}$ = 7,0 Hz, C<u>H</u>$_3$CH$_2$OP, 6H), 1.51 (s, C(C<u>H</u>$_3$)$_3$, 9H), 2.81 (t, CHOC<u>H</u>$_2$CH$_2$, $^3J_{HH}$ = 7.2 Hz, 2H), 2.94 (t, CHOCH$_2$C<u>H</u>$_2$, $^3J_{HH}$ = 7.2 Hz, 2H), 3.94 –4.23 (m, CH$_3$C<u>H</u>$_2$OP, 4H), 7.19 (dd, $^3J_{HH}$ = 9.1 Hz, $^4J_{HH}$ = 1.6 Hz, C<u>H</u>$_{(7)}$, 1H), 7.56 (dd, $^3J_{HH}$ = 9.1 Hz, $^4J_{HH}$ = 0.5 Hz, C<u>H</u>$_{(8)}$, 1H), 7.78 (d, $^3J_{PH}$ = 24.0 Hz, C<u>H</u>=CP, 1H), 8.18 (bs, C<u>H</u>$_{(5)}$, 1H), 8.38 (s, C<u>H</u>$_{(2)}$, 1H), 9.77 (t, $^3J_{HH}$ = 0.9 Hz, C<u>H</u>O, 1H), ^{31}**P NMR** (283 MHz, $CDCl_3$) δ: 16.81 – *(E)* CH=CP, 13.46 – *(Z)* CH=CP, (E:Z)=(1:0.16), ^{13}**C NMR** (176 MHz, $CDCl_3$) δ: 16.29 (d, $^4J_{PC}$ = 6.9 Hz, <u>C</u>H$_3$CH$_2$OP, 2C), 24.97 (s, CHOCH$_2$<u>C</u>H$_2$, 1C), 28.03 (s, C(<u>C</u>H$_3$)$_3$, 3C), 44.59 (s, CHO<u>C</u>H$_2$CH$_2$, 1C), 62.43 (d, $^3J_{PC}$ = 5.2 Hz, CH$_3$<u>C</u>H$_2$OP, 2C), 82.59 (s, <u>C</u>(CH$_3$)$_3$, 1C), 117.60 (d, $^1J_{PC}$ = 182.7 Hz, <u>C</u>P, 1C), 118.05 (s, <u>C</u>H$_{(8)}$, 1C), 120.27 (d, $^3J_{PC}$ = 24.8 Hz, <u>C</u>$_{(3)}$, 1C), 122.05 (s, <u>C</u>H$_{(5)}$, 1C), 127.07 (s, <u>C</u>$_{(6)}$, 1C), 128.95 (s, <u>C</u>H$_{(7)}$, 1C), 132.39 (d, $^2J_{PC}$ = 10.2 Hz, <u>C</u>H=CP, 1C), 140.61 (s, <u>C</u>H$_{(2)}$, 1C), 146,69 (s, C$_{(9)}$, 1C), 165.00 (d, $^2J_{PC}$ = 11.3 Hz, <u>C</u>O$_2$*t*-Bu, 1C), 200.19 (s, <u>C</u>HO, 1C).

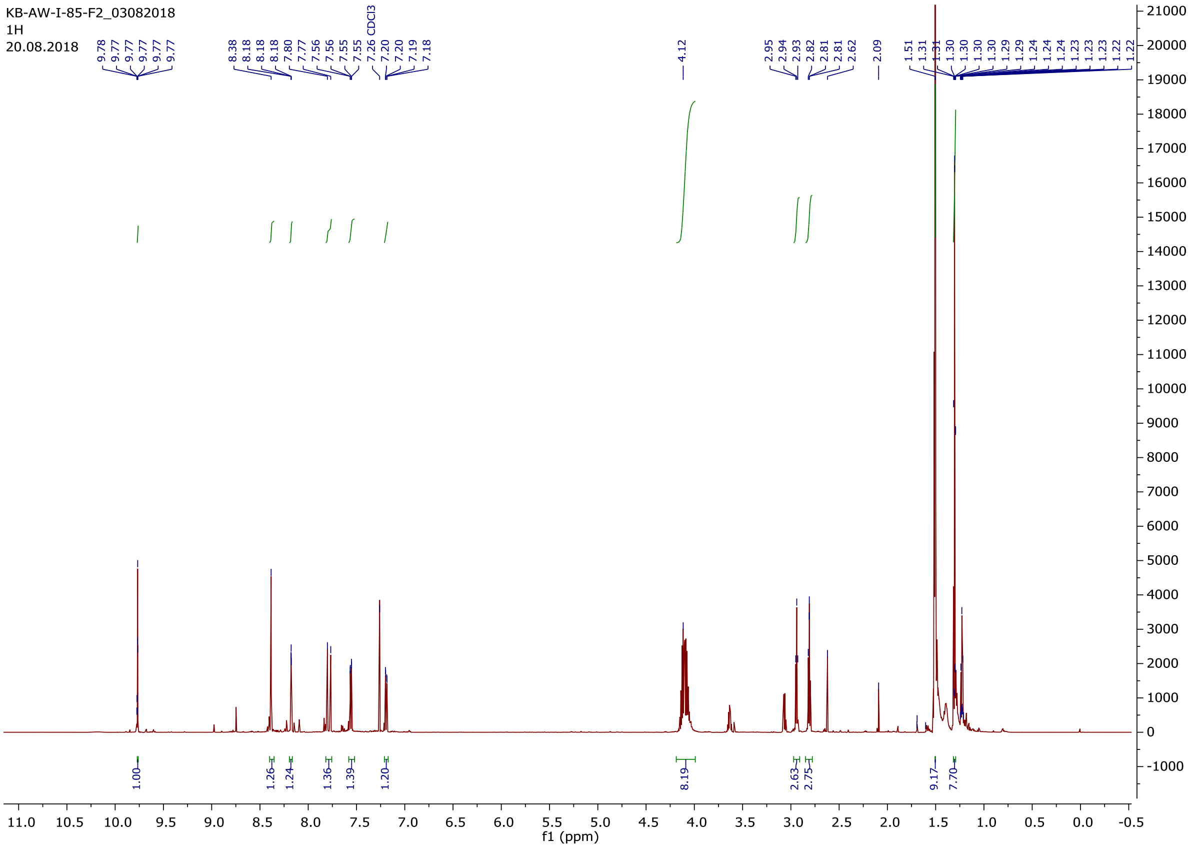

*Figure S23. ^{1}H NMR of compound **12** (residual DIPEAxHBr at 1.5, 3.1, 3.6 ppm) (700 MHz, $CDCl_3$)*

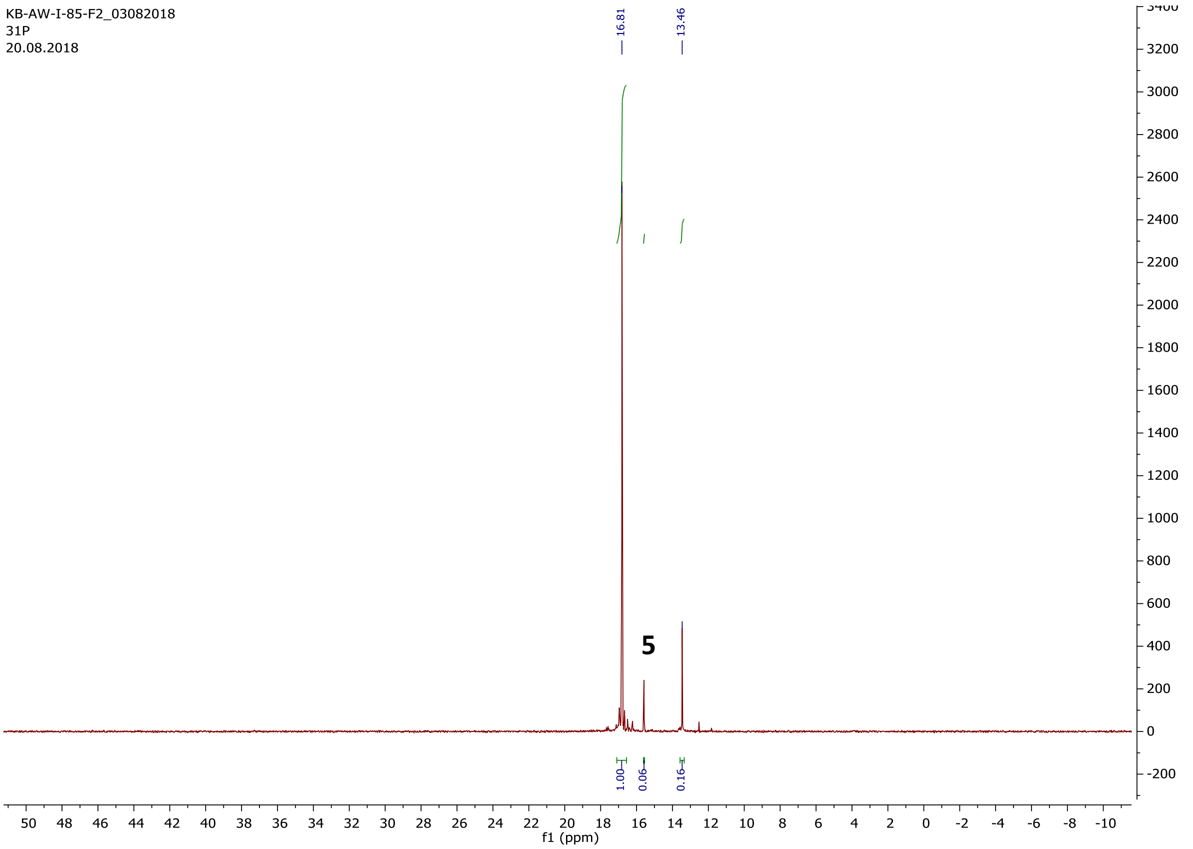

*Figure S24. ^{31}P NMR of compound **12** (ratio E/Z 100/16) (283 MHz, $CDCl_3$)*

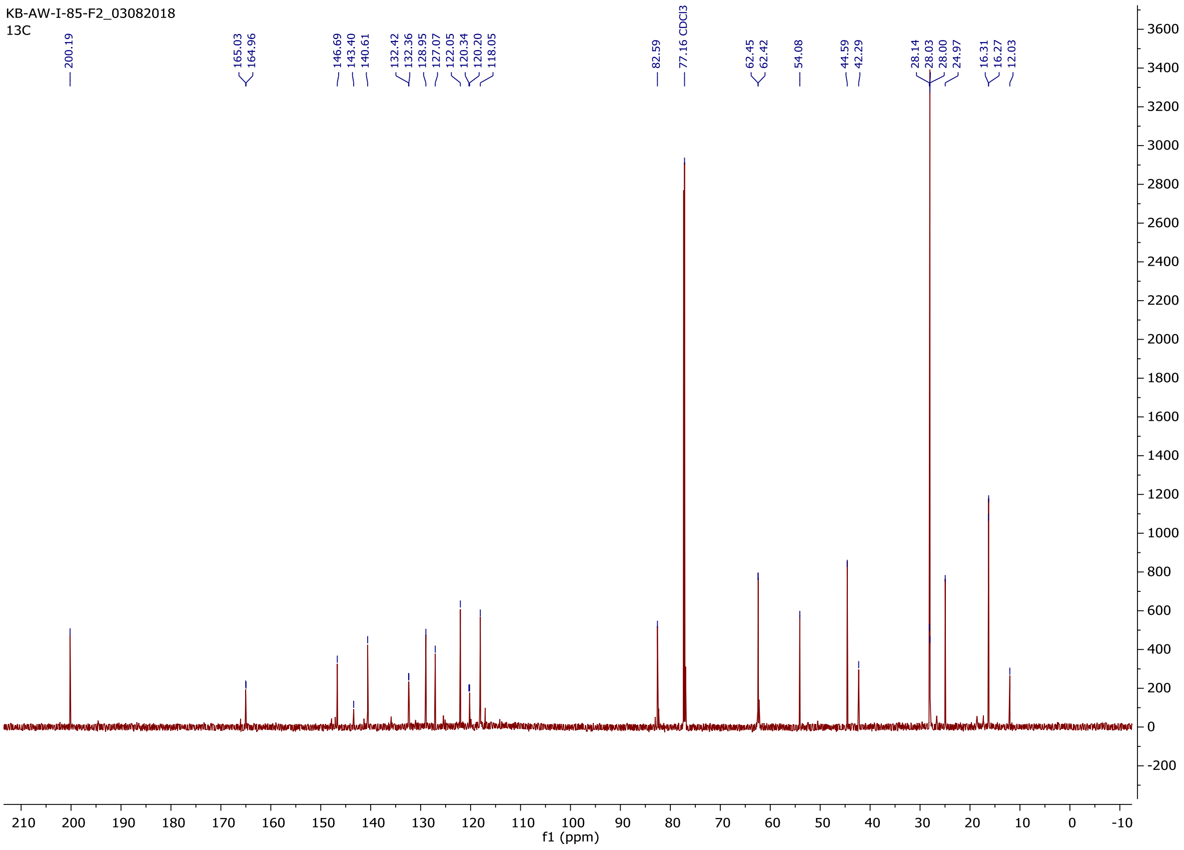

Figure S25. ^{13}*C NMR of compound* ***12*** *(176 MHz, $CDCl_3$)*

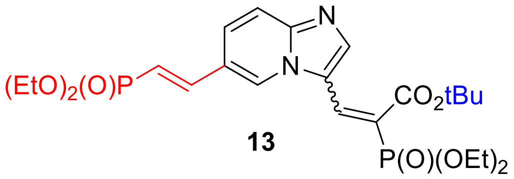

13

***tert*-Butyl 2-(diethoxyphosphoryl)-3-(6-((*E*)-2-(diethoxyphosphoryl)vinyl)imidazo[1,2-*a*]pyridin-3-yl)acrylate (13).** Yield 65%.

HRMS m/z: calculated 543.2020 ($C_{24}H_{36}N_2O_8P_2$ + H)$^+$, found 543.2030 ($C_{24}H_{36}N_2O_8P_2$ + H)$^+$, **^{1}H NMR** (700 MHz, $CDCl_3$) δ: 1.33 (t, $^3J_{HH}$ = 7.1 Hz, ($C\underline{H}_3CH_2)_2OP$, 6H), 1.34 (t, $^3J_{HH}$ = 7.1 Hz, ($C\underline{H}_3CH_2)_2OP$, 6H), 1.53 (s, $C(C\underline{H}_3)_3$, 9H), 4.07–4.18 (m, ($CH_3C\underline{H}_2O)_2P$, 8H), 6.30 (dd, $^3J_{HH}$ = 17.4 Hz, $^2J_{PH}$ = 16.5 Hz, PC$\underline{H}$=CH, 1H), 7.41 (dd, $^3J_{PH}$ = 22.2 Hz, $^3J_{HH}$ = 17.4 Hz, PCH=C$\underline{H}$, 1H), 7.51 (dd, $^3J_{HH}$ = 9.3 Hz, $^4J_{HH}$ = 1.6 Hz, C$\underline{H}_{(7)}$, 1H), 7.71 (d, $^3J_{HH}$ = 9.3 Hz, C$\underline{H}_{(8)}$, 1H), 7.78 (d, $^3J_{HH}$ = 23.9 Hz, *(E)* C$\underline{H}$=CP, 1H), 8.38 (bs, C$\underline{H}_{(5)}$, 1H), 8.41 (s, C$\underline{H}_{(2)}$, 1H), **^{31}P NMR** (283 MHz, $CDCl_3$) δ: 16.01 – *(E)* CH=C$\underline{P}$, 17.46– *(E)* CH=CH$\underline{P}$, 12.89 – *(Z)* CH=CP, 17.62–*(Z)* CH=CH$\underline{P}$, (E:Z) CH=C$\underline{P}$ =(1:0.18), **^{13}C NMR** (176 MHz, $CDCl_3$) δ: 16.37 (d, $^3J_{PC}$ = 7.0 Hz, ($\underline{C}H_3CH_2)_2OP$, 2C), 16.48 (d, $^3J_{PC}$ = 6.3 Hz, ($\underline{C}H_3CH_2)_2OP$, 2C), 28.09 (s, $C(\underline{C}H_3)_3$, 3C), 62.19 (d, $^2J_{PC}$ = 5.6 Hz, ($CH_3\underline{C}H_2)_2OP$, 2C), 62.57 (d, $^2J_{PC}$ = 5.3 Hz, ($CH_3\underline{C}H_2O)_2P$, 2C), 82.88 (s, $\underline{C}(CH_3)_3$, 1C), 116.67 (d, $^1J_{PC}$ = 192.4 Hz, P$\underline{C}$H=CH, 1C), 118.65 (s, $\underline{C}H_{(8)}$, 1C), 119.76 (d, $^1J_{PC}$ = 181.7 Hz, $\underline{C}$P, 1C), 121.06 (d, $^3J_{PC}$ = 25,0 Hz, $\underline{C}_{(3)}$, 1C), 122.95 (d, $^3J_{PC}$ = 24.4 Hz, $\underline{C}_{(6)}$, 1C), 124.24 (s, $\underline{C}H_{(7)}$, 1C), 124.62 (s, $\underline{C}H_{(5)}$, 1C), 131.58 (d, $^2J_{PC}$ = 10.0 Hz, $\underline{C}$H=CP, 1C), 141.07 (s, $\underline{C}H_{(2)}$, 1C), 143.18 (d, $^2J_{PC}$ = 7,0 Hz, CH=$\underline{C}$H, 1C), 147.28 (s, $C_{(9)}$, 1C), 164.87 (d, $^2J_{PC}$ = 10.9 Hz, $\underline{C}O_2$*t*-Bu, 1C).

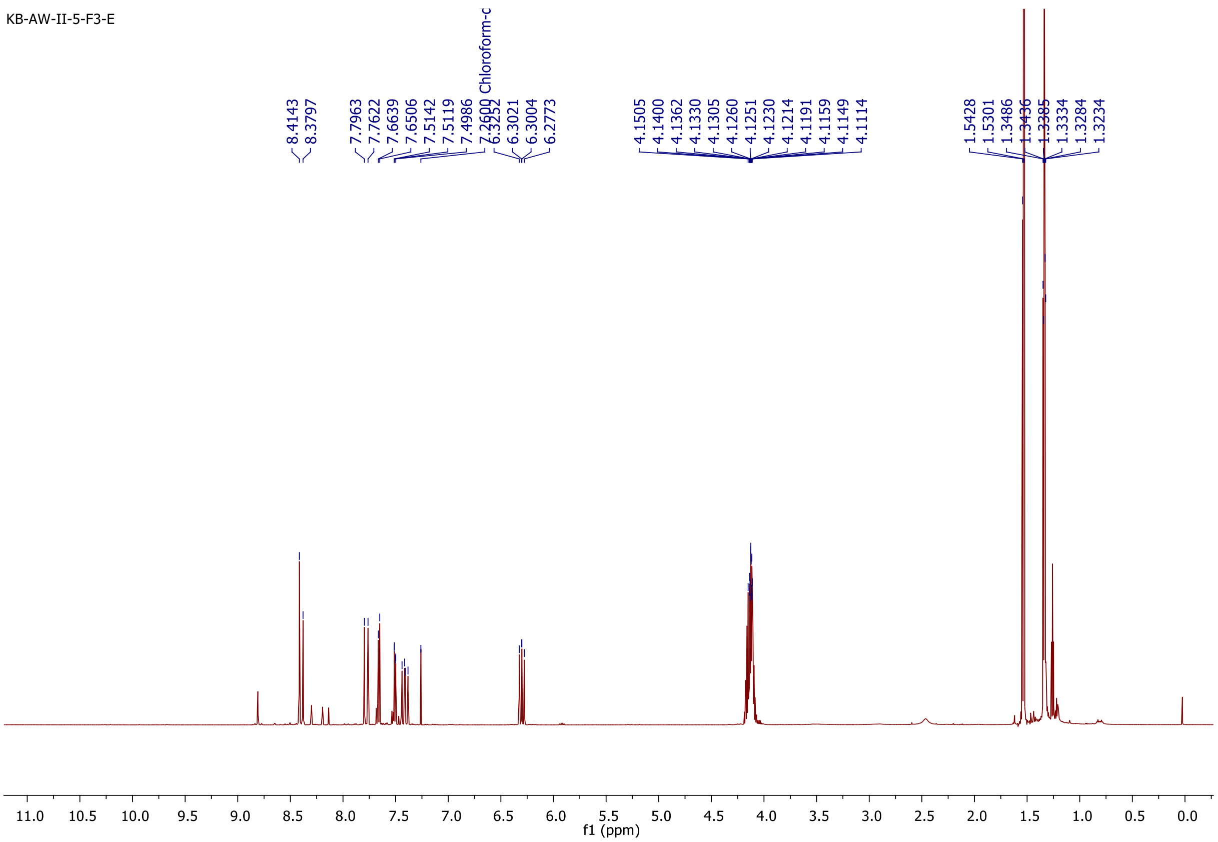

Figure S26. ^{1}*H NMR of compound* ***13*** *(700 MHz, CDCl*$_3$*)*

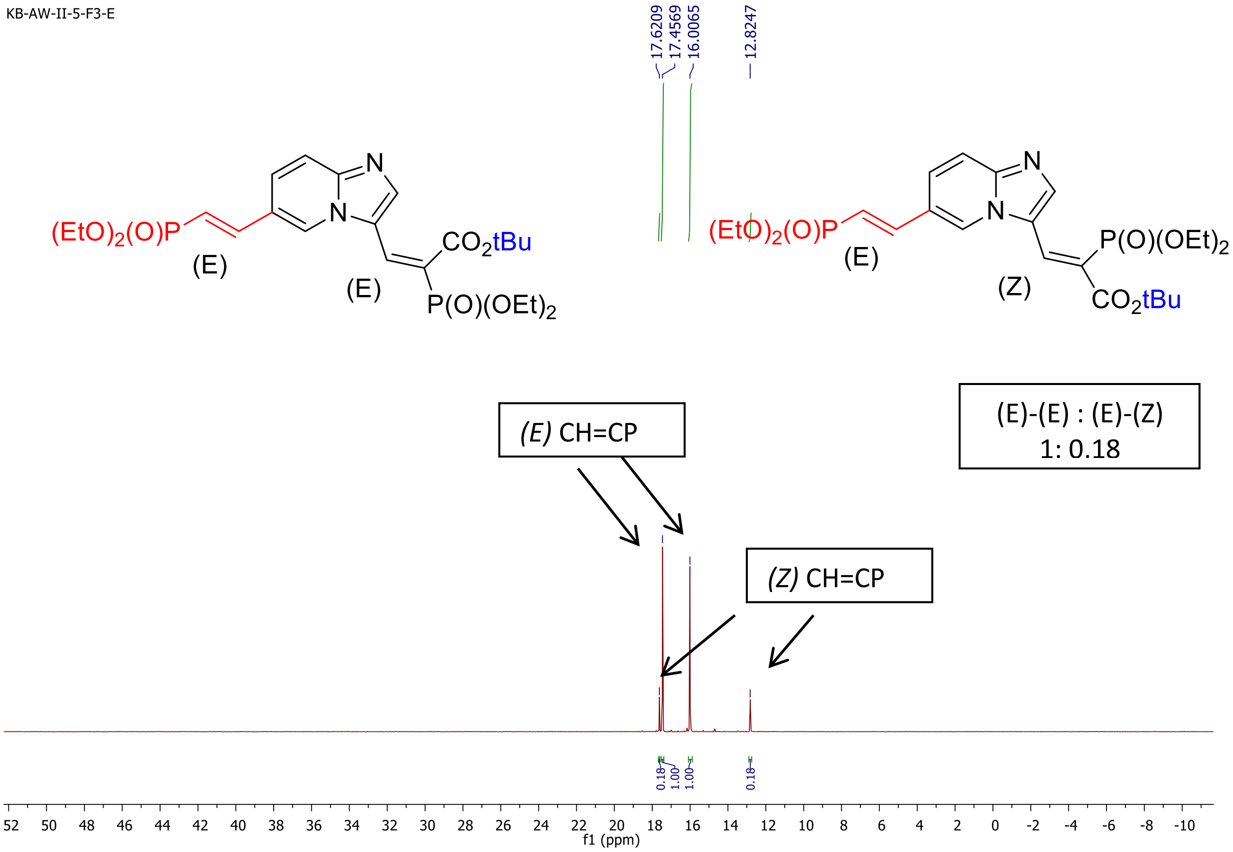

Figure S27. ^{31}*P NMR of compound* ***13*** *(283 MHz, CDCl*$_3$*)*

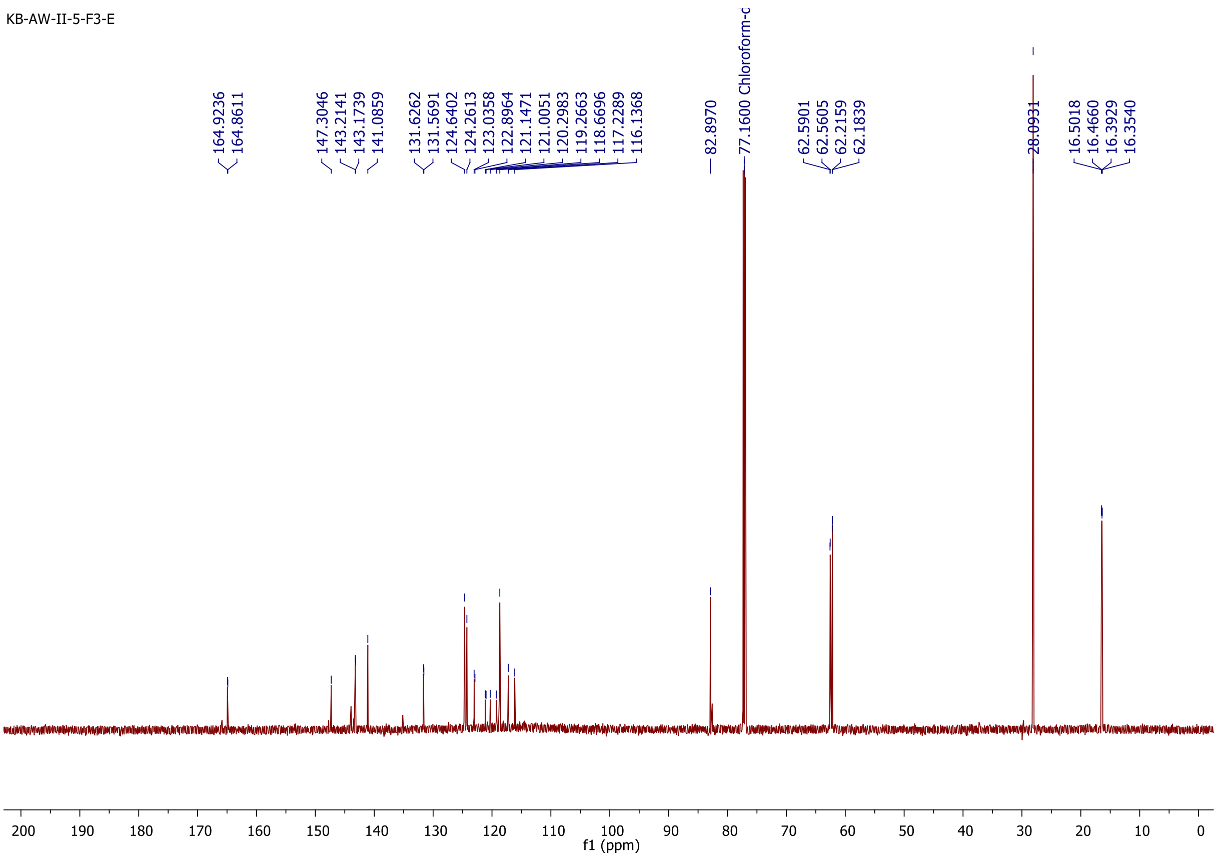

*Figure S28. ^{13}C NMR of compound **13** (176 MHz, $CDCl_3$)*

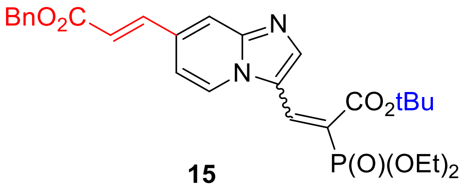

15

***tert*-Butyl 3-(7-((*E*)-3-(benzyloxy)-3-oxoprop-1-en-1-yl)imidazo[1,2-*a*]pyridin-3-yl)-2-(diethoxyphosphoryl)acrylate (15).** Yield 65%

HRMS m/z: calculated 541.2098 ($C_{28}H_{33}N_2O_7P$ + H)$^+$, found 541.2115 ($C_{28}H_{33}N_2O_7P$ + H)$^+$, **^{1}H NMR** (700 MHz, $CDCl_3$) δ: 1.35 (t, $^3J_{HH}$ = 7.0 Hz, C<u>H</u>$_3$CH$_2$OP, 6H), 1.55 (s, C(C<u>H</u>$_3$)$_3$, 9H), 4.06 – 4.22 (m, CH$_3$C<u>H</u>$_2$OP, 4H), 5.25 (s, PhC<u>H</u>$_2$O, 2H), 6.55 (d, $^3J_{HH}$ = 15.9 Hz, BnOC(O)C<u>H</u>=CH, 1H), 7.14 (d, $^3J_{HH}$ = 7.2 Hz, C<u>H</u>$_{(6)}$, 1H), 7.31 – 7.41 (m, <u>Ph</u>CH$_2$O, 5H), 7.69 (d, $^3J_{HH}$ = 15.9 Hz, BnOC(O)CH=C<u>H</u>, 1H), 7.74 (bs, C<u>H</u>$_{(8)}$, 1H), 7.81 (d, $^3J_{HH}$ = 24.0 Hz, *(E)* C<u>H</u>=CP, 1H), 8.33 (d, $^3J_{HH}$ = 7.2 Hz, C<u>H</u>$_{(5)}$, 1H), 8.47 (s, C<u>H</u>$_{(2)}$, 1H), **^{31}P NMR** (283 MHz, $CDCl_3$) δ: 16.58 – *(E)* CH=CP, 13.28 – *(Z)* CH=CP (E:Z)=(1:0.12), **^{13}C NMR** (176 MHz, $CDCl_3$) δ: 16.38 (d, $^4J_{PC}$ = 6.8 Hz, <u>C</u>H$_3$CH$_2$OP, 2C), 28.12 (s, C(<u>C</u>H$_3$)$_3$, 3C), 62.61 (d, $^3J_{PC}$ = 5,2 Hz CH$_3$<u>C</u>H$_2$OP, 2C), 66.80 (s, Ph<u>C</u>H$_2$O, 1C), 82.95 (s, <u>C</u>(CH$_3$)$_3$, 1C), 111.61 (s, <u>C</u>H$_{(6)}$, 1C), 119.20 (s, <u>C</u>H$_{(8)}$, 1C), 119.65 (d, $^1J_{PC}$ = 182.0 Hz, <u>C</u>P, 1C), 120.92 (s, BnOC(O)<u>C</u>H=CH, 1C), 121.34 (d, $^3J_{PC}$ = 24.9 Hz, <u>C</u>$_{(3)}$, 1C), 123.91 (s, <u>C</u>H$_{(5)}$, 1C), 128.44, 128.48, 128.72 (s, Ph, 5C), 131.77 (d, $^2J_{PC}$ = 10.2 Hz, <u>C</u>H=CP, 1C), 133.26 (s, <u>C</u>$_{(7)}$, 1C), 135.81 (s, Ph – C$_{IV}$, 1C), 141.55 (s, <u>C</u>H$_{(2)}$, 1C), 142.02 (s, BnOC(O)CH=<u>C</u>H, 1C), 147.24 (s, C$_9$, 1C), 164.92 (d, $^2J_{PC}$ = 11.1 Hz, <u>C</u>O$_2$tBu, 1C), 166.05 (s, BnO<u>C</u>(O)CH=CH, 1C).

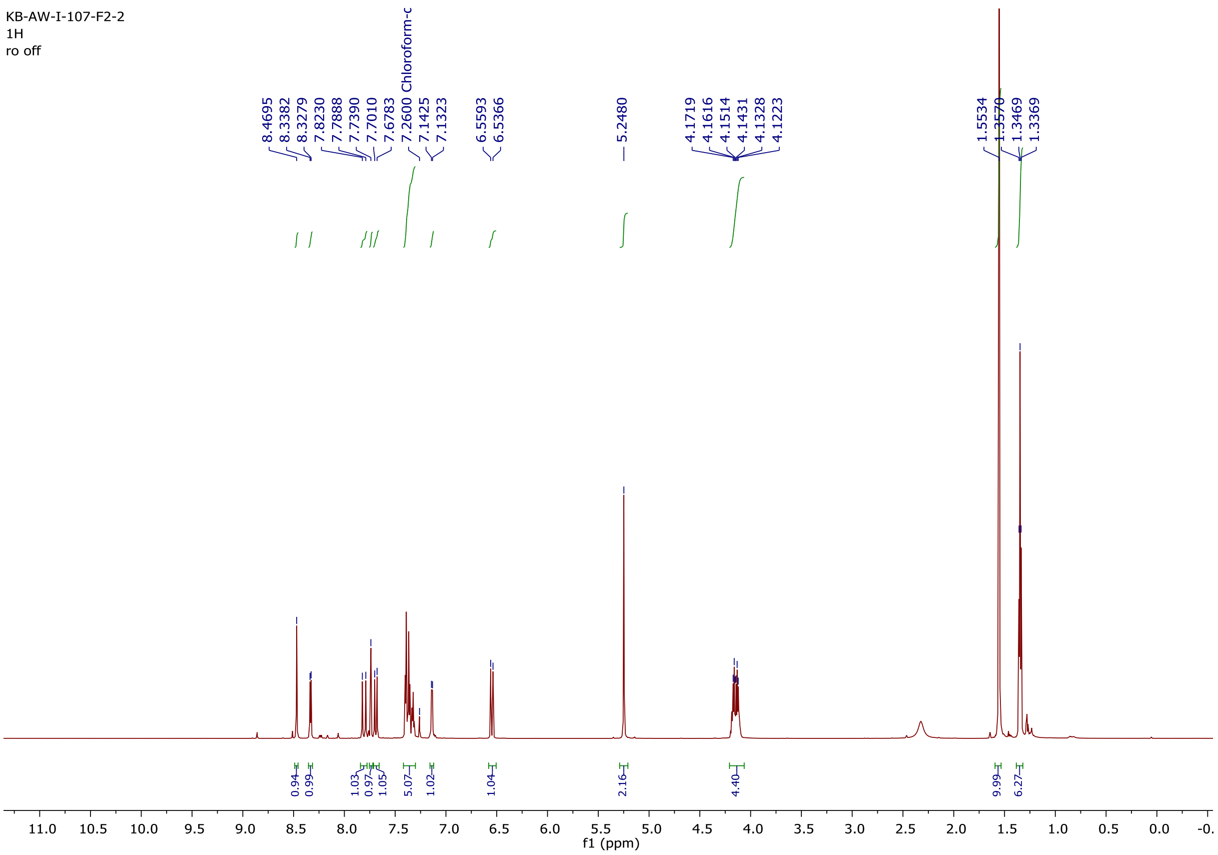

Figure S29. ^{1}H NMR of compound **15** (700 MHz, $CDCl_3$)

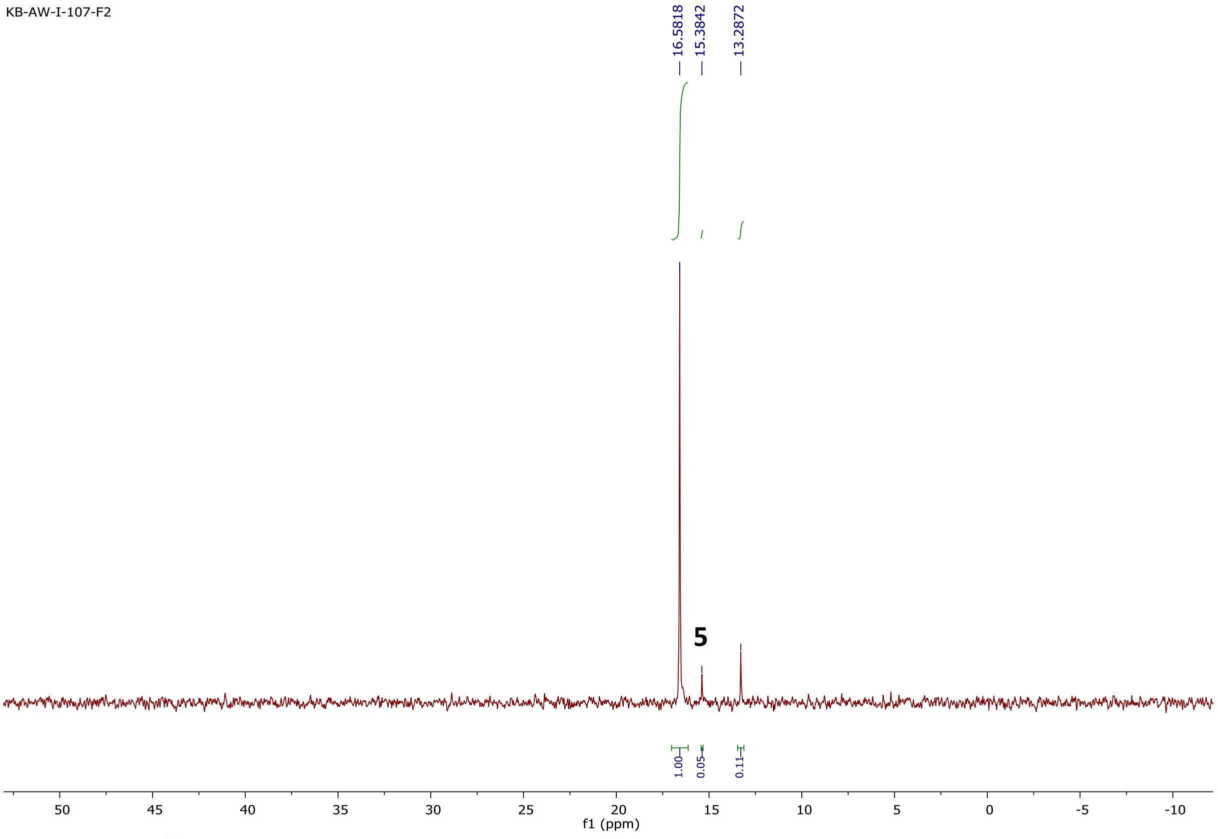

Figure S30. ^{31}P NMR of compound **15** (283 MHz, $CDCl_3$)

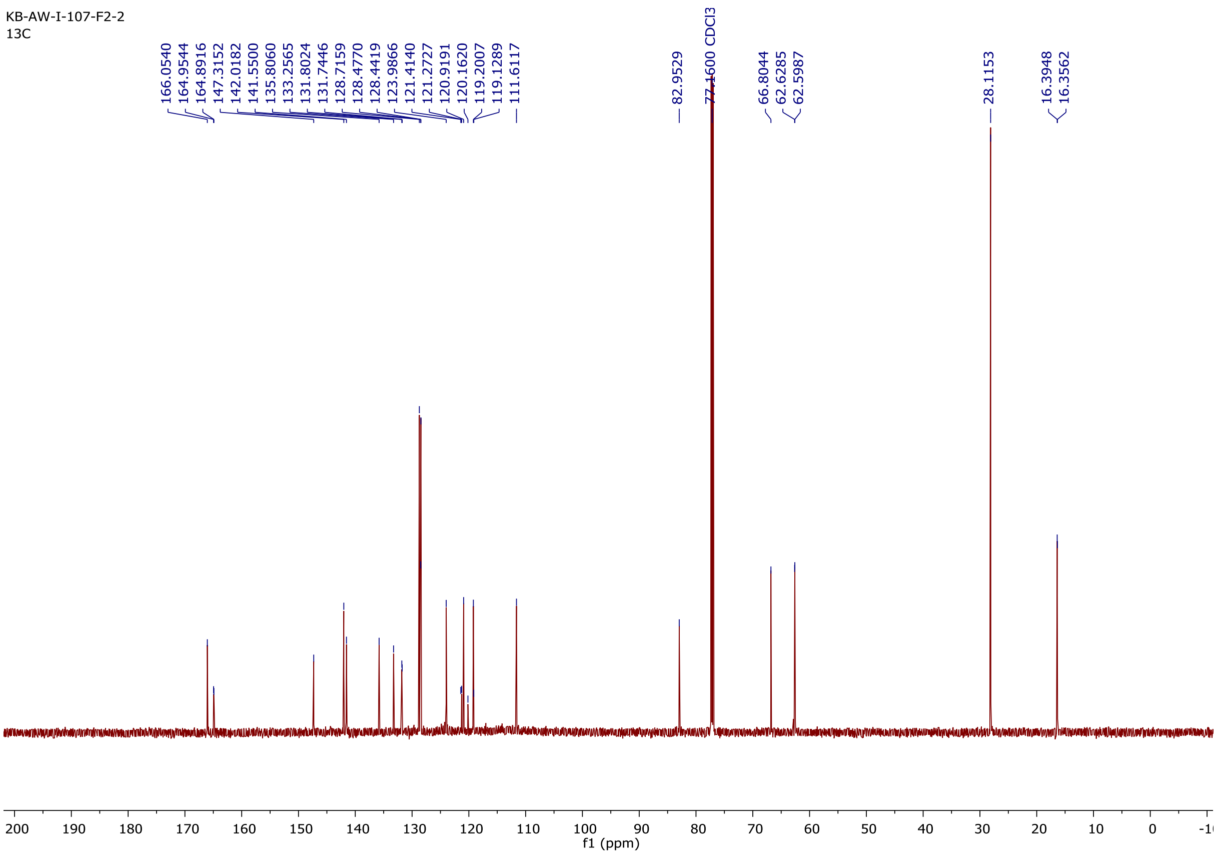

Figure S31. ^{13}C NMR of compound ***15*** *(176 MHz, $CDCl_3$)*

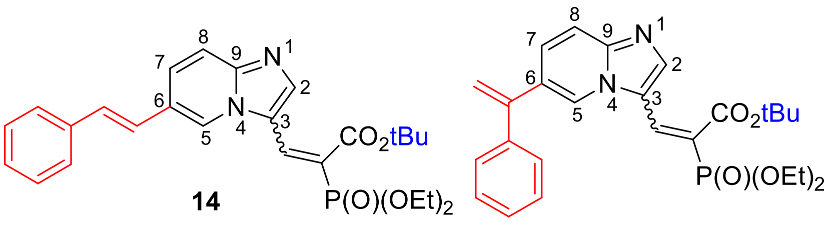

Major product Other possible Heck product

***tert*-Butyl 2-(diethoxyphosphoryl)-3-(6-((*E*)-styryl)imidazo[1,2-*a*]pyridin-3-yl)acrylate (14),** Yield 60%.

^{1}H NMR (700 MHz, $CDCl_3$) δ: 1.38 (t, $^3J_{HH}$ = 7.1 Hz, $C\underline{H}_3CH_2OP$, 6H), 1.58 (s, $C(C\underline{H}_3)_3$, 9H), 4.09 – 4.29 (m, $CH_3C\underline{H}_2OP$, 4H), 7.09 (d, $^3J_{HH}$ = 16.3 Hz, PhC$\underline{H}$=CH, 1H), 7.15 (d, $^3J_{HH}$ = 16.3 Hz, PhCH=C$\underline{H}$, 1H), 7.28-7.34 (m, Ph, 1H), 7.37-7.42 (m, Ph, 2H), 7.50-7.54 (m, Ph, 2H), 7.67 (dd, $^3J_{HH}$ = 9.4 Hz, $^4J_{HH}$ = 1.5 Hz, $C\underline{H}_{(7)}$, 1H), 7.68 (dd, $^3J_{HH}$ = 9.3 Hz, $^5J_{HH}$ = 0.7 Hz $C\underline{H}_{(8)}$, 1H), 7.89 (d, $^3J_{HH}$ = 24.0 Hz, *(E)* C$\underline{H}$=CP, 1H), 8.35 (bs, $C\underline{H}_{(5)}$, 1H), 8.46 (s, $C\underline{H}_{(2)}$, 1H), **^{31}P NMR** (283 MHz, $CDCl_3$) δ: 16.75 – *(E)* CH=CP, 13.45 – *(Z)* CH=CP, (E:Z)=(1:0.3), **^{13}C NMR** (176 MHz, $CDCl_3$) δ: 16.48 (d, $^4J_{PC}$ = 6.9 Hz, $\underline{C}H_3CH_2OP$, 2C), 28.22 (s, $C(\underline{C}H_3)_3$, 3C), 62.57 (d, $^3J_{CP}$ = 5.1 Hz, $CH_3\underline{C}H_2OP$, 2C), 82.79 (s, $\underline{C}(CH_3)_3$, 1C), 118.31 (d, $^1J_{PC}$ = 182.3 Hz, $\underline{C}$P, 1C), 118.36 (s, $\underline{C}H_{(8)}$, 1C), 120.83 (d, $^3J_{PC}$ = 24.7 Hz, $\underline{C}_{(3)}$, 1C), 122.03 (s, $\underline{C}H_{(5)}$, 1C), 123.66 (s, PhCH=$\underline{C}$H, 1C), 124.94 (s, $\underline{C}H_{(7)}$, 1C), 125.43 (s, $\underline{C}_{(6)}$, 1C), 126.74 (s, Ph, 2C), 128.44 (s, Ph, 1C), 129.01 (s, Ph, 2C), 130.81 (s, Ph$\underline{C}$H=CH, 1C), 132.33 (d, $^2J_{PC}$ = 10.2 Hz, $\underline{C}$H=CP, 1C), 136.54 (s, Ph-C_{IV}, 1C), 140.94 (s, $\underline{C}H_{(2)}$, 1C), 147.12 (s, $C_{(9)}$, 1C), 165.18 (d, $^2J_{PC}$ = 11.3 Hz, $\underline{C}O_2t$-Bu, 1C),

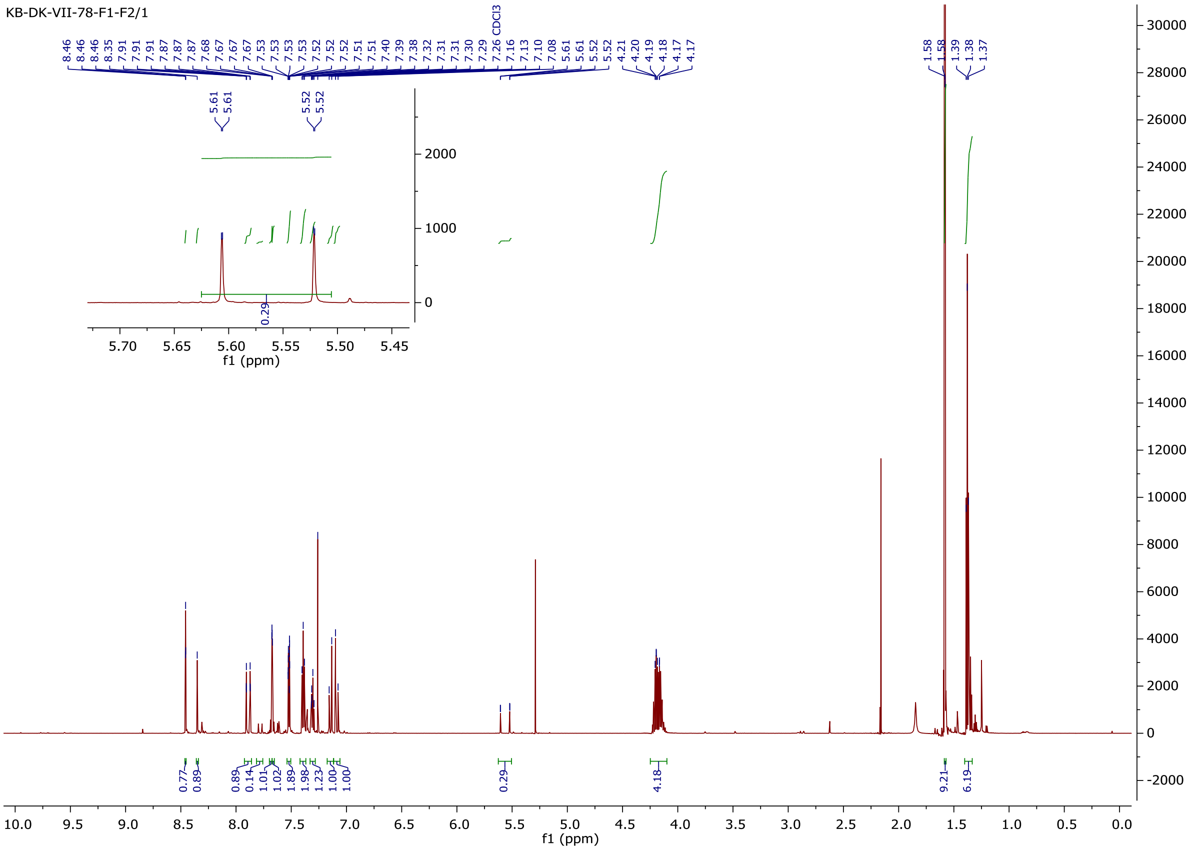

Figure S32. ^{1}H NMR of compound **14** (700 MHz, $CDCl_3$)

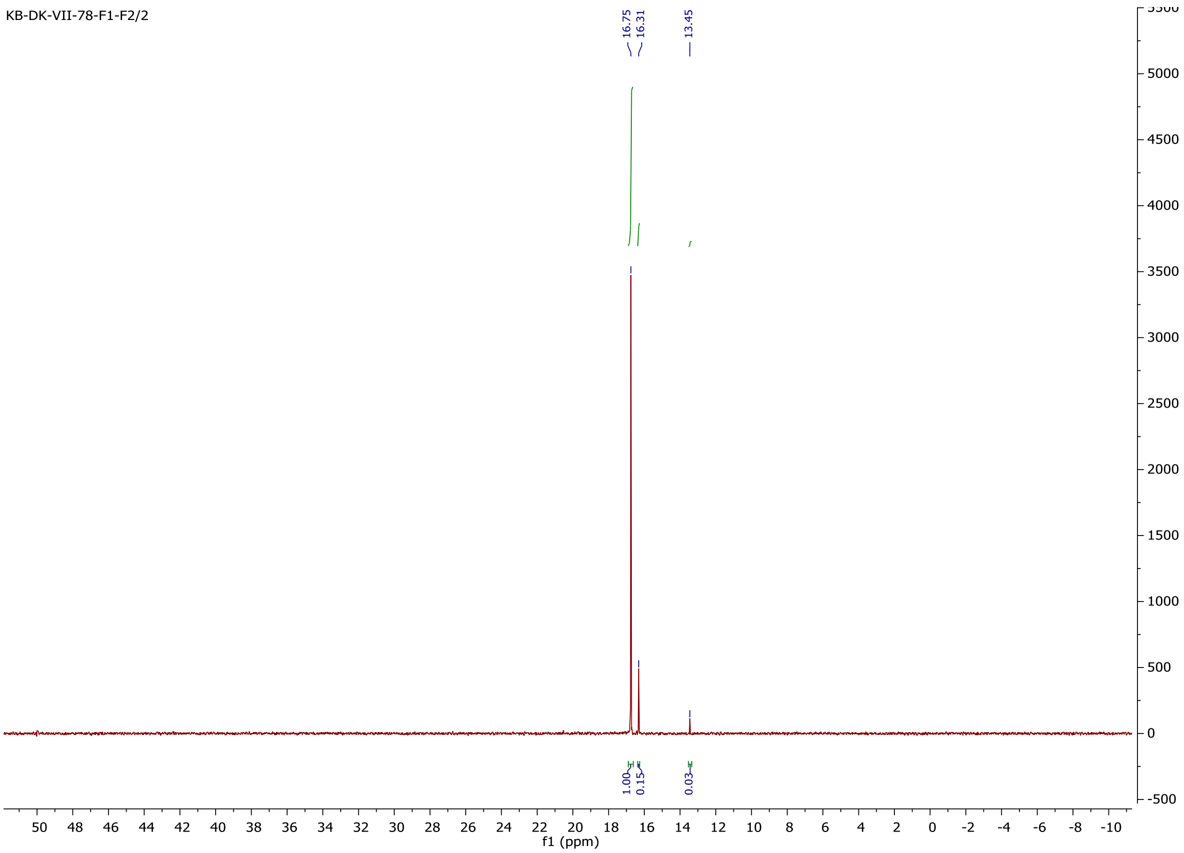

Figure S33. ^{31}P NMR of compound **14** (mixture of Heck products) (283 MHz, $CDCl_3$)

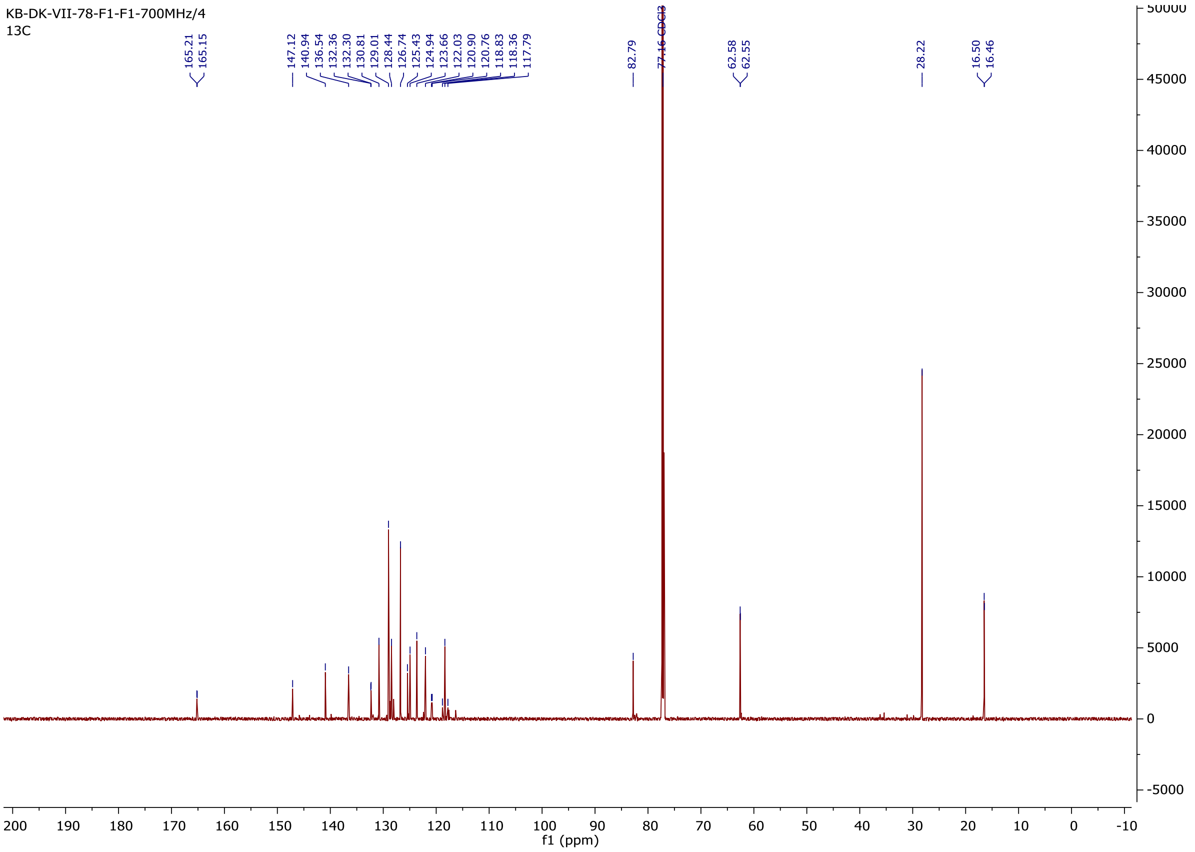

*Figure S34. ^{13}C NMR of compound **14** (176 MHz, $CDCl_3$)*

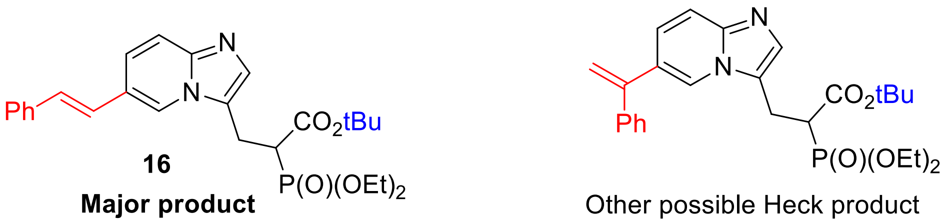

16

Major product

Other possible Heck product

(*E*)-*tert*-Butyl 2-(diethoxyphosphoryl)-3-(6-styrylimidazo[1,2-*a*]pyridin-3-yl)propanoate (16), Yield 84%

^{1}H NMR (700 MHz, $CDCl_3$) δ: 1.33-1.39 (m, (C<u>H</u>$_3$CH$_2$O)$_2$(O)P, C(C<u>H</u>$_3$)$_3$, 15H), 3.26 (ddd, $^{2}J_{PH}$ = 22.7, $^{3}J_{HH}$ = 11.5, $^{3}J_{HH}$ = 2.9, CH$_2$C<u>H</u>P, 1H), 3.34 (ddd, $^{2}J_{HH}$ = 15.7, $^{3}J_{PH}$ = 10.1, $^{3}J_{HH}$ = 2.9, C<u>H</u>$_2$CHP, 1H), 3.57 (ddd, $^{2}J_{HH}$ = 15.7, $^{3}J_{HH}$ = 11.5, $^{3}J_{PH}$ = 6.7, C<u>H</u>$_2$CHP, 1H), 4.16-4.23 (m, (CH$_3$C<u>H</u>$_2$O)$_2$P, 4H), 7.04 (d, $^{3}J_{AB}$ = 16.2, PhCH=C<u>H</u>, 1H), 7.07 (d, $^{3}J_{AB}$ = 16.2, PhC<u>H</u>=CH, 1H), 7.23-7.28 (m, <u>Ph</u>, 1H), 7.32-7.37 (m, <u>Ph</u>, 2H), 7.40 (s, C<u>H</u>$_{(2)}$, 1H), 7.47 (dd, $^{3}J_{HH}$ = 9.4, $^{4}J_{HH}$ = 1.7, C<u>H</u>$_{(7)}$, 1H), 7.48-7.50 (m, <u>Ph</u>, 2H), 7.56 (dd, $^{3}J_{HH}$ = 9.4, $^{5}J_{HH}$ = 0,8, C<u>H</u>$_{(8)}$, 1H), 8.02 (bs, C<u>H</u>$_{(5)}$, 1H), **^{31}P NMR** (283 MHz, $CDCl_3$) δ: 21.61, **^{13}C NMR** (176 MHz, $CDCl_3$) δ: 16.44-16.57 (m, <u>C</u>H$_3$CH$_2$OP, 2C), 21.67 (d, $^{2}J_{PC}$ = 3.4 Hz, <u>C</u>H$_2$CHP, 1C), 27.88 (s, C(<u>C</u>H$_3$)$_3$, 3C), 45.32 (d, $^{1}J_{PC}$ = 128.8 Hz, CH$_2$<u>C</u>HP, 1C), 63.04 (d, $^{2}J_{PC}$ = 7.0 Hz, CH$_3$<u>C</u>H$_2$OP, 1C), 63.11 (d, $^{2}J_{PC}$ = 6.4 Hz, CH$_3$<u>C</u>H$_2$OP, 1C), 82.68 (s, <u>C</u>(CH$_3$)$_3$, 1C), 117.89 (s, <u>C</u>H$_{(8)}$, 1C), 121.65 (s, <u>CH</u>$_{(7)}$, 1C), 121.70 (s, <u>C</u>H$_{(5)}$, 1C), 121.89 (d, $^{3}J_{PC}$ = 19.1 Hz, <u>C</u>$_{(3)}$, 1C), 123.45 (s, <u>C</u>$_{(6)}$, 1C), 124.49 (s, PhCH=<u>C</u>H, 1C), 126.52 (s, <u>Ph</u>, 2C), 127.99 (s, <u>Ph</u>, 1C), 128.83 (s, <u>Ph</u>, 2C), 129.39 (s, Ph<u>C</u>H=CH, 1C), 132.05 (s, <u>C</u>H$_{(2)}$, 1C), 136.83 (s, <u>Ph-C$_{IV}$</u>, 1C), 145.10 (s, C$_{(9)}$, 1C), 167.34 (d, $^{2}J_{PC}$ = 5.2 Hz, <u>C</u>O$_2$*t*-Bu, 1C).

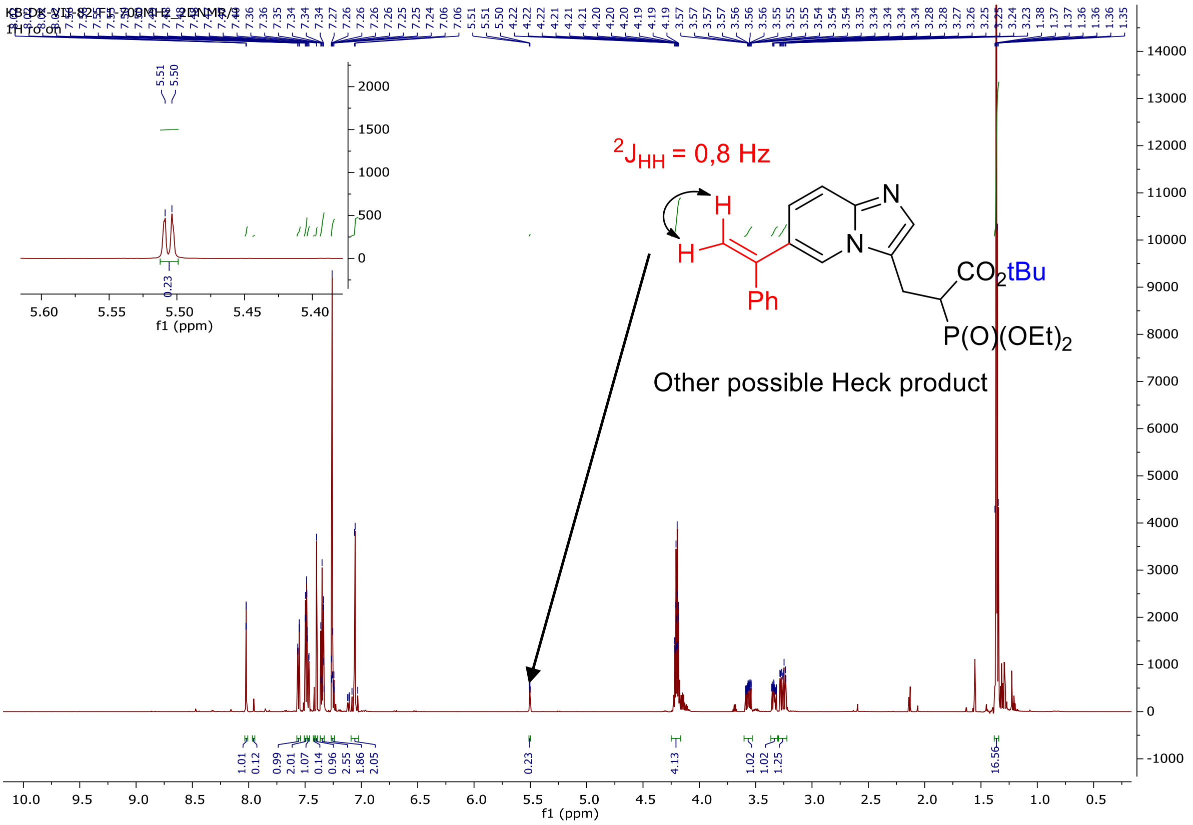

*Figure S35. ^{1}H NMR of compound **16** (700 MHz, $CDCl_3$)*

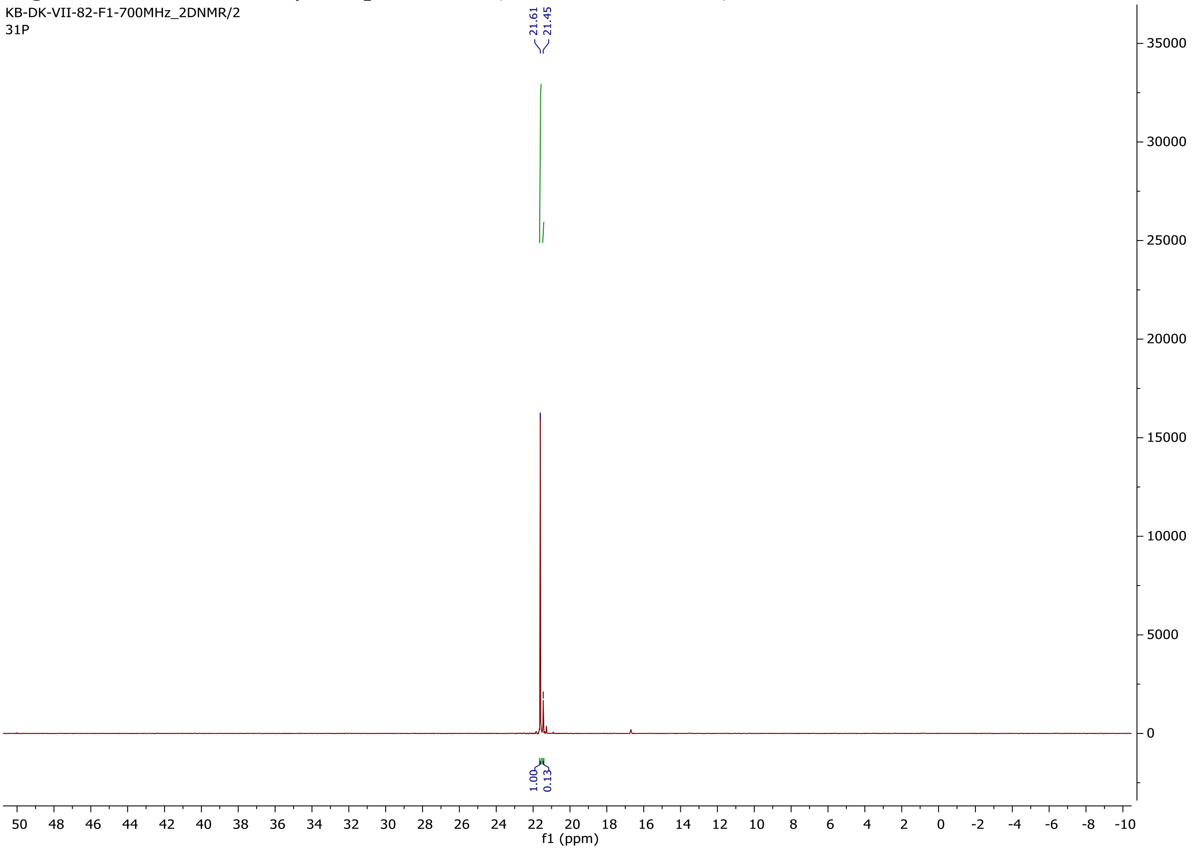

*Figure S36. ^{31}P NMR of compound **16 (**mixture of Heck products) (283 MHz, $CDCl_3$)*

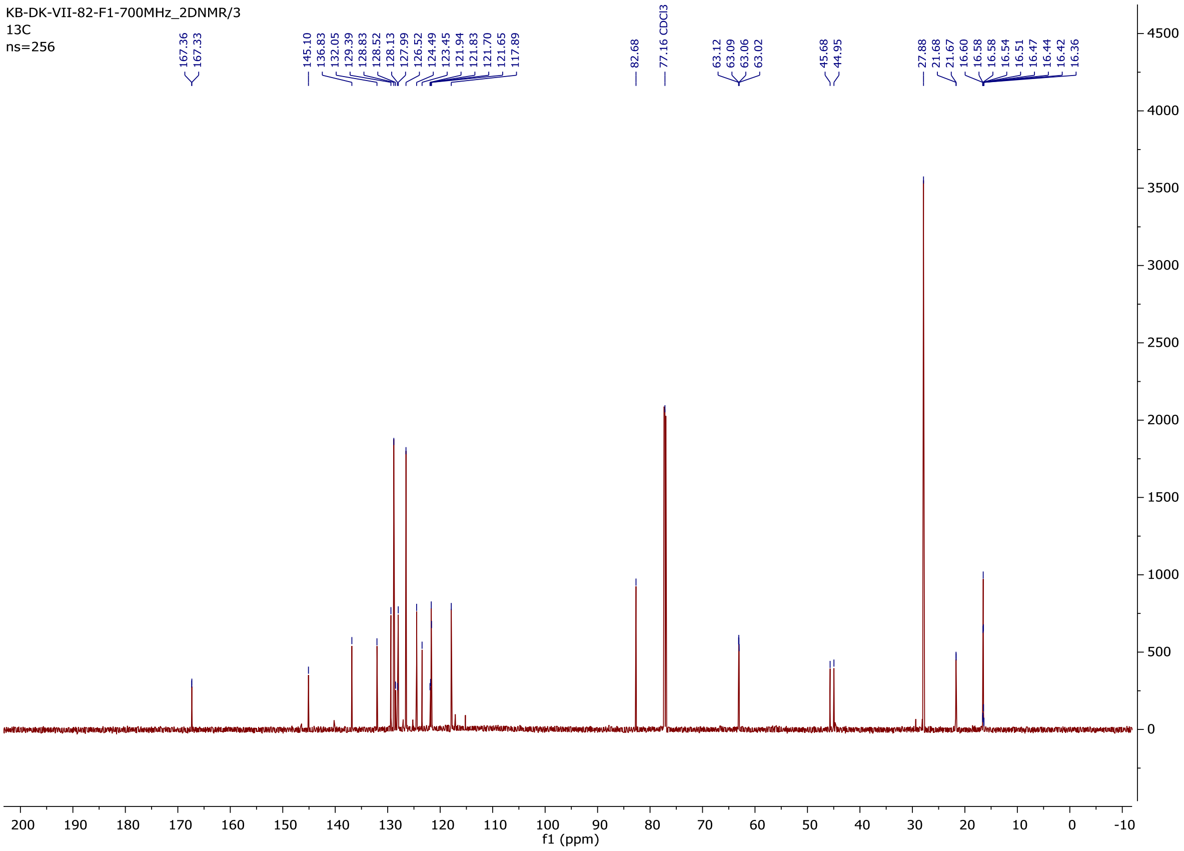

*Figure S37. ^{13}C NMR of compound **16** (176 MHz, $CDCl_3$)*

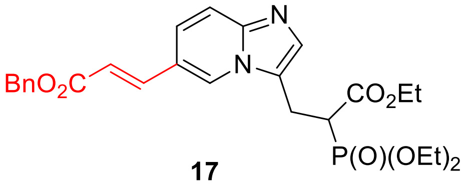

17

(*E*)-Benzyl 3-(3-(2-(diethoxyphosphoryl)-3-ethoxy-3-oxopropyl)imidazo[1,2-*a*]pyridin-6-yl)acrylate (17). Yield 58%

^{1}H NMR (700 MHz, $CDCl_3$) δ: 1.20 (t, $^3J_{HH}$ = 7.1 Hz, C̲H̲$_3$CH$_2$OC, 3H), 1.37 (t, $^3J_{HH}$ = 7.1 Hz, (C̲H̲$_3$CH$_2$O)$_2$P, 6H), 3.35 (ddd, $^2J_{PH}$ = 23.0, $^3J_{HH}$ = 11.6, $^3J_{PH}$ = 3.3, CH$_2$CH̲P, 1H), 3.36-3.41 (m, CH̲$_2$CHP, 1H), 3.61 (ddd, $^2J_{HH}$ = 16.5, $^3J_{HH}$ = 11.6, $^3J_{PH}$ = 7.2, CH̲$_2$CHP, 1H), 4.06 – 4.25 (m, (CH$_3$CH̲$_2$O)$_2$P, CH$_3$CH̲$_2$OC, 6H), 5.27 (s, PhCH̲$_2$, 2H), 6.49 (d, $^3J_{HH}$ = 15.9 Hz, *Bn*OC(O)CH̲=CH, 1H), 7.34-7.38 (m, CH$_2$C$_6$H̲$_5$, 1H), 7.37-7.42 (m, CH$_2$C$_6$H̲$_5$, CH̲$_{(7)}$, 3H), 7.42-7.46 (m, CH$_2$C$_6$H̲$_5$, CH̲$_{(2)}$, 3H), 7.59 (d, $^3J_{HH}$ = 9.5 Hz, CH̲$_{(8)}$, 1H), 7.72 (d, $^3J_{HH}$ = 15.9 Hz, *Bn*OC(O)CH=CH̲, 1H), 8.15 (bs, CH̲$_{(5)}$, 1H), **^{31}P NMR** (283 MHz, $CDCl_3$) δ: 20.95, **^{13}C NMR** (176 MHz, $CDCl_3$) δ: 13.96 (s, C̲H$_3$CH$_2$OC, 1C), 16.34 (d, $^3J_{PC}$ = 6.2 Hz, C̲H$_3$CH$_2$OP, 1C), 16.38 (d, $^3J_{PC}$ =6.1 Hz, C̲H$_3$CH$_2$OP, 1C), 21.44 (d, $^2J_{PC}$ = 3.7 Hz, C̲H$_2$CHP, 1C), 44.40 (d, $^1J_{PC}$ = 129.6 Hz, CH$_2$C̲HP, 1C), 61.93 (s, CH$_3$C̲H$_2$OC, 1C), 63.12 (d, $^2J_{PC}$ = 6.9 Hz, CH$_3$C̲H$_2$OP, 1C), 63.24 (d, $^2J_{PC}$ = 6.2 Hz, CH$_3$C̲H$_2$OP, 1C), 66.47 (s, C̲H$_2$Ph, 1C), 118.26 (s, C̲H$_{(8)}$, 1C), 118.43 (s, *Bn*OC(O)C̲H=CH, 1C), 120.66 (s, C̲$_{(6)}$, 1C), 121.12 (s, C̲H$_{(7)}$, 1C), 122.25 (d, $^3J_{PC}$ = 18.2 Hz, C̲$_{(3)}$, 1C), 125.08 (s, C̲H$_{(5)}$, 1C), 128.28 (s, CH$_2$C̲$_6$H$_5$, 3C), 128.58 (s, CH$_2$C̲$_6$H$_5$, 2C), 132.68 (s, C̲H$_{(2)}$, 1C), 135.87 (s, CH$_2$C̲$_6$H$_5$ - C$_{IV}$, 1C), 141.03 (s,

*Bn*OC(O)CH=C̲H, 1C), 145.33 (s, $C_{(9)}$, 1C), 166.20 (s, *Bn*O C̲(O)CH=CH, 1C), 168.12 (d, $^{2}J_{PC}$ = 5,2 Hz, C̲O_2*Et*, 1C).

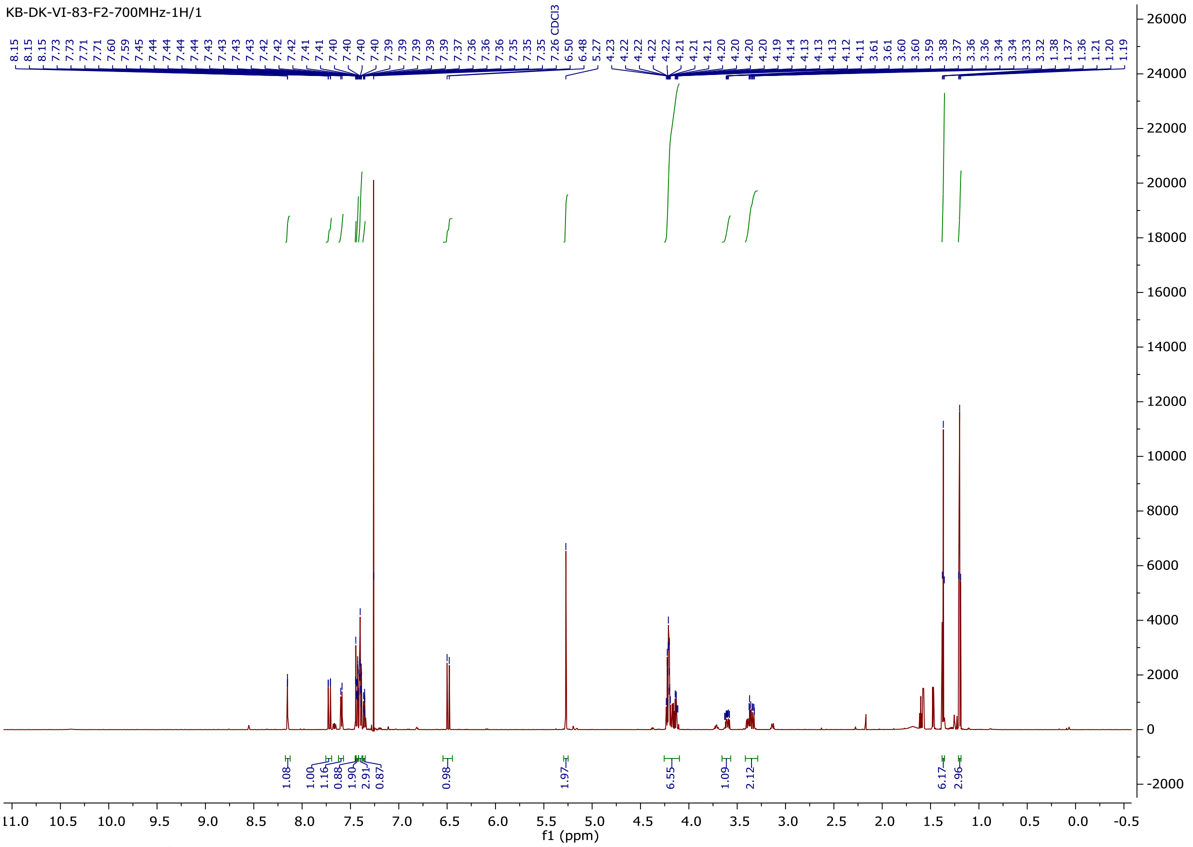

Figure S38. ^{1}H *NMR of compound* ***17*** *(700 MHz,* $CDCl_3$*)*

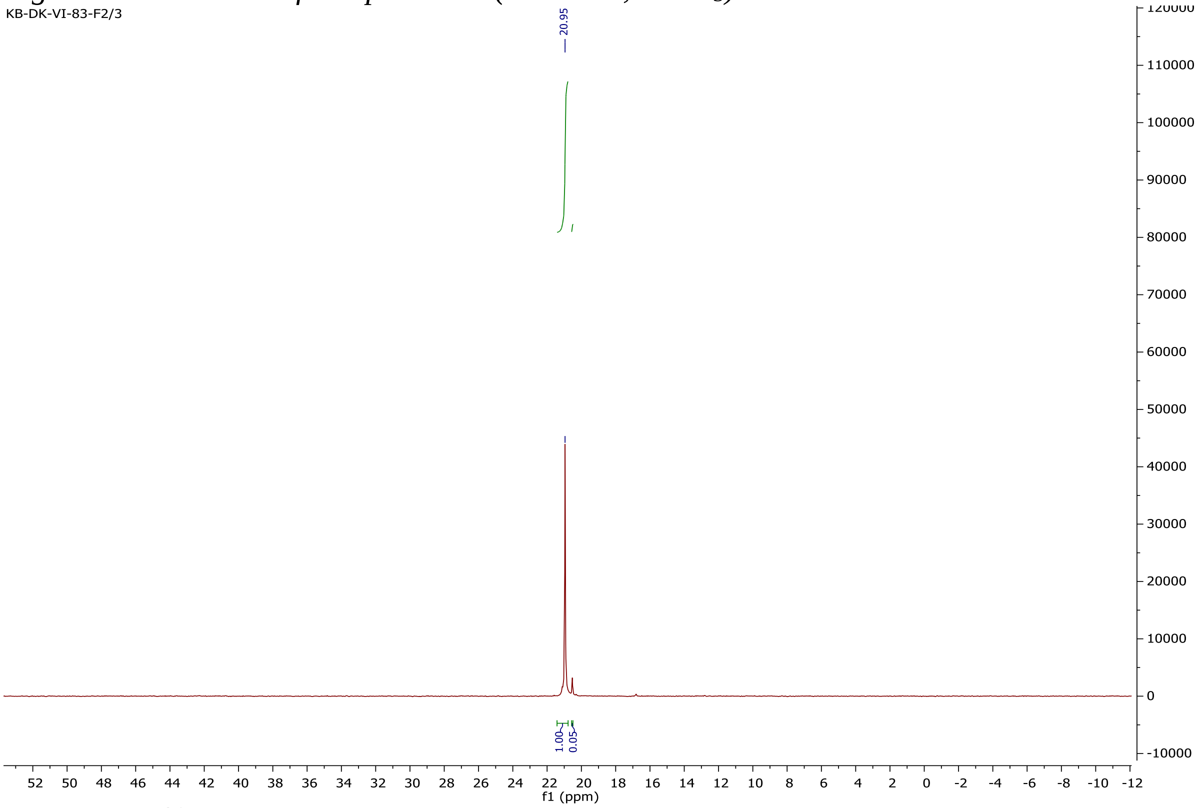

Figure S39. ^{31}P *NMR of compound* ***17*** *(283 MHz,* $CDCl_3$*)*

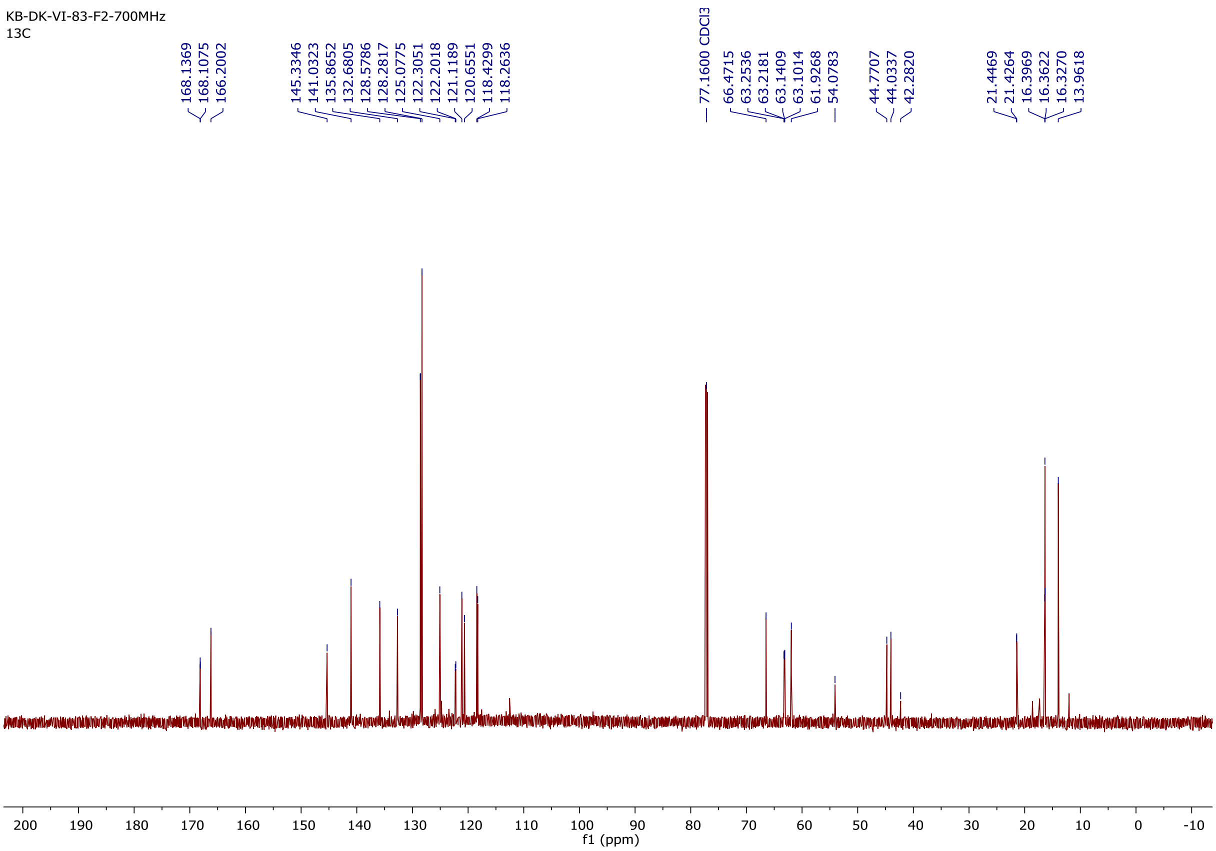

*Figure S40. ^{13}C NMR of compound **17** (176 MHz, $CDCl_3$)*

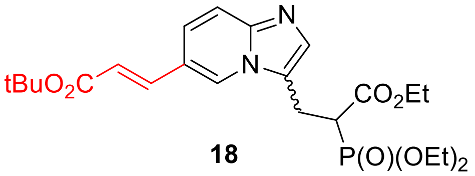

18

(*E*)-*tert*-Butyl 3-(3-(2-(diethoxyphosphoryl)-3-ethoxy-3-oxopropyl)imidazo[1,2-*a*]pyridin-6-yl)acrylate (18). Yield 66%

HRMS m/z: calculated 481.2098 ($C_{23}H_{33}N_2O_7P$ + H)$^+$, found 481.2106 ($C_{23}H_{33}N_2O_7P$ + H)$^+$, **^{1}H NMR** (700 MHz, $CDCl_3$) δ: 1.20 (t, $^3J_{HH}$ = 7.1 Hz, C<u>H</u>$_3$CH$_2$OC, 3H), 1.37 (t, $^3J_{HH}$ = 7.1 Hz, (C<u>H</u>$_3$CH$_2$O)$_2$P, 6H), 1.55 (s, C(C<u>H</u>$_3$)$_3$, 9H), 3.31-3.42 (m, C<u>H</u>$_2$C<u>H</u>P, 2H) 3.61 (ddd, $^2J_{HH}$ = 16.3, $^3J_{HH}$ = 11.6, $^3J_{PH}$ = 7.2, C<u>H</u>$_2$CHP, 1H), 4.09 – 4.25 (m, (CH$_3$C<u>H</u>$_2$O)$_2$P, CH$_3$C<u>H</u>$_2$OC, 6H), 6.37 (d, $^3J_{HH}$ = 15.8 Hz, *t*-BuOC(O)C<u>H</u>=CH, 1H), 7.40 (dd, $^3J_{HH}$ = 9.4 Hz, $^4J_{HH}$ = 1.7, C<u>H</u>$_{(7)}$, 1H), 7.44 (s, C<u>H</u>$_{(2)}$, 1H), 7.57 (d, $^3J_{HH}$ = 15.8 Hz, *t*-BuOC(O)CH=C<u>H</u>, 1H), 7.60 (d, $^3J_{HH}$ = 9.4 Hz, C<u>H</u>$_{(8)}$, 1H), 8.12 (bs, C<u>H</u>$_{(5)}$, 1H), **^{31}P NMR** (283 MHz, $CDCl_3$) δ: 20.63, **^{13}C NMR** (176 MHz, $CDCl_3$) δ: 14.15 (s, <u>C</u>H$_3$CH$_2$OC, 1C), 16.54 (d, $^3J_{PC}$ = 6.0 Hz, <u>C</u>H$_3$CH$_2$OP, 1C), 16.58 (d, $^3J_{PC}$ = 6.0 Hz, <u>C</u>H$_3$CH$_2$OP, 1C), 28.35 (s, C(<u>C</u>H$_3$)$_3$, 3C), 21.66 (d, $^2J_{PC}$ = 3.5 Hz, <u>C</u>H$_2$CHP, 1C), 44.60 (d, $^1J_{PC}$ = 129.6 Hz, CH$_2$<u>C</u>HP, 1C), 62.14 (s, CH$_3$<u>C</u>H$_2$OC, 1C), 63.31 (d, $^2J_{PC}$ = 6.9 Hz, CH$_3$<u>C</u>H$_2$OP, 1C), 63.43 (d, $^2J_{PC}$ = 6.6 Hz, CH$_3$<u>C</u>H$_2$OP, 1C), 81.02 (s, <u>C</u>(CH$_3$)$_3$, 1C), 118.33 (s, <u>C</u>H$_{(8)}$, 1C), 121.16 (s, *t*-BuOC(O)<u>C</u>H=CH, 1C), 121.25 (s, <u>C</u>$_{(6)}$, 1C), 121.68 (s, <u>C</u>H$_{(7)}$, 1C), 122.32 (d, $^3J_{PC}$ = 18.4 Hz, <u>C</u>$_{(3)}$, 1C), 124.62 (s, <u>C</u>H$_{(5)}$, 1C), 132.56 (s, <u>C</u>H$_{(2)}$, 1C), 139.44 (s, *t*-BuOC(O)CH=<u>C</u>H, 1C), 145.43 (s, C$_{(9)}$, 1C), 165.86 (s, *t*-BuO<u>C</u>(O)CH=CH, 1C), 168.29 (d, $^2J_{PC}$ = 5.2 Hz, <u>C</u>O$_2$*Et*, 1C).

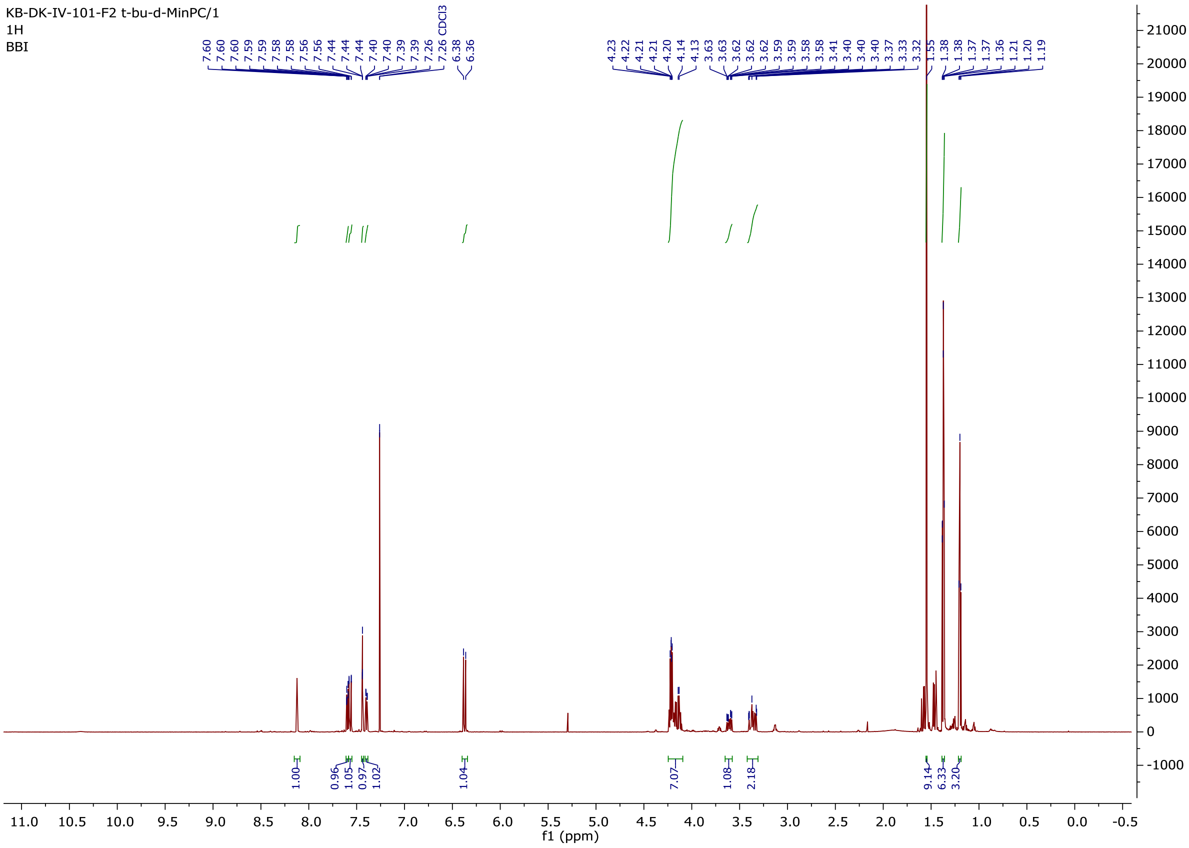

*Figure S41. ^{1}H NMR of compound **18** (700 MHz, $CDCl_3$)*

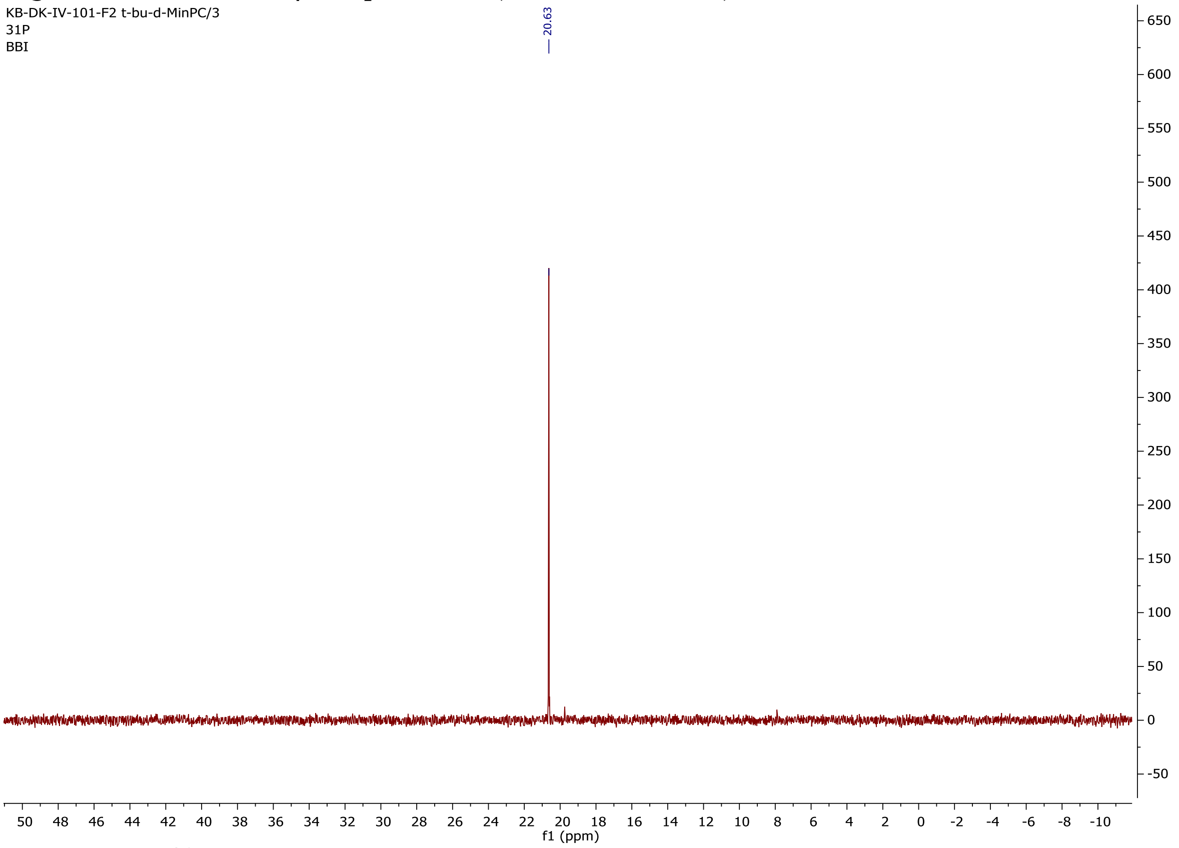

*Figure S42. ^{31}P NMR of compound **18** (286 MHz, $CDCl_3$)*

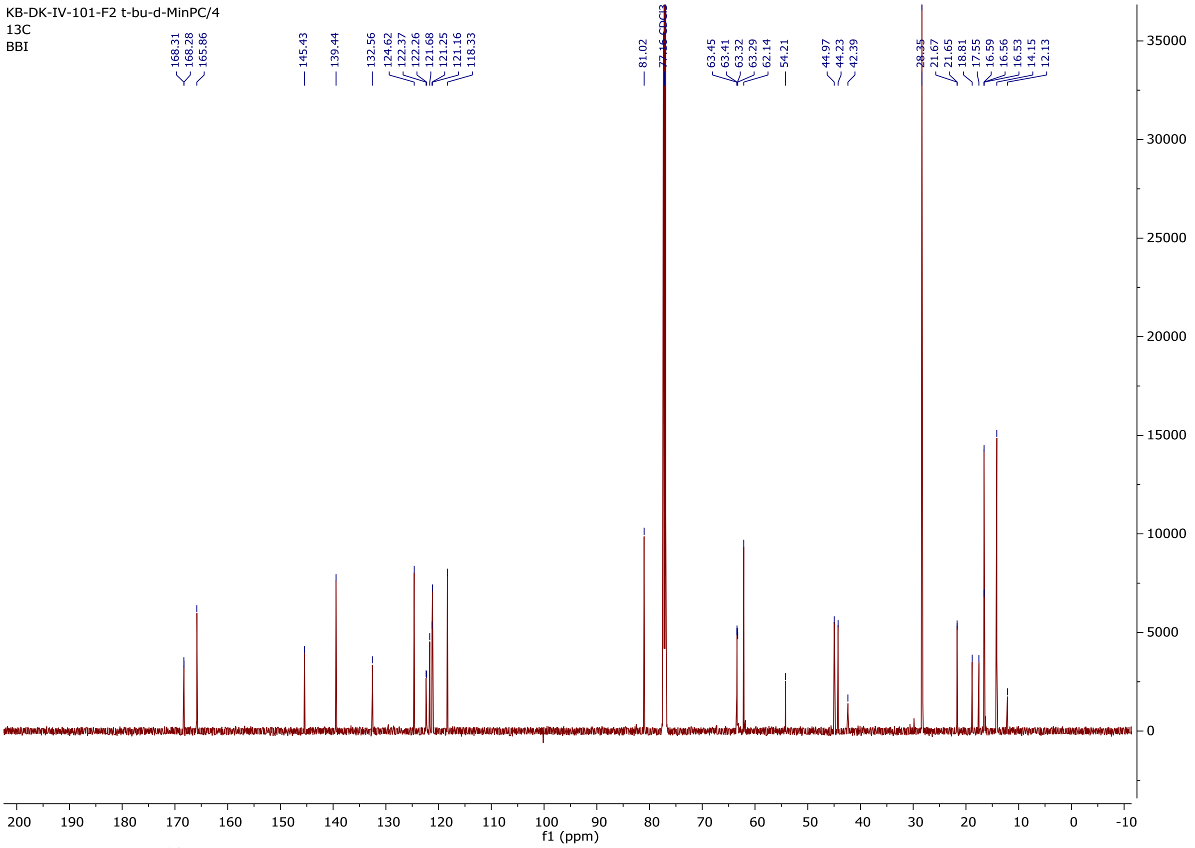

*Figure S43. ^{13}C NMR of compound **18** (176 MHz, CDCl$_3$)*

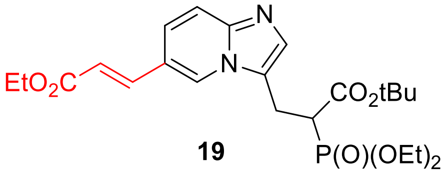

19

(*E*)-Ethyl 3-(3-(3-(*tert*-butoxy)-2-(diethoxyphosphoryl)-3-oxopropyl)imidazo[1,2-*a*]pyridin-6-yl)acrylate (19). Yield 67%.

HRMS m/z: calculated 481.2098 ($C_{23}H_{33}N_2O_7P$ + H)$^+$, found 481.2097 ($C_{23}H_{33}N_2O_7P$ + H)$^+$, **^{1}H NMR** (700 MHz, CDCl$_3$) δ: 1.33 (t, $^3J_{HH}$ = 7.1 Hz, C$\underline{H_3}$CH$_2$OC(O)CH=CH, 3H), 1.35-1.37 (m, (C$\underline{H_3}$CH$_2$O)$_2$(O)P, C(C$\underline{H_3}$)$_3$, 15H), 3.23 (ddd, $^2J_{PH}$ = 22.8, $^3J_{HH}$ = 11.5, $^3J_{HH}$ = 3.2, CH$_2$C$\underline{H}$P, 1H), 3.33 (ddd, $^2J_{HH}$ = 15.7, $^3J_{PH}$ = 10.0, $^3J_{HH}$ = 3.2, C$\underline{H_2}$CHP, 1H), 3.55 (ddd, $^2J_{HH}$ = 15.7, $^3J_{HH}$ = 11.5, $^3J_{PH}$ = 6.9, C$\underline{H_2}$CHP, 1H), 4.15-4.22 (m, (CH$_3$C$\underline{H_2}$O)$_2$P, 4H), 4.26 (q, $^3J_{HH}$ = 7.1 Hz, CH$_3$C$\underline{H_2}$OC(O)CH=CH, 2H), 6.41 (d, $^3J_{HH}$ = 15.9 Hz, (CH$_3$CH$_2$OC(O)C$\underline{H}$=CH, 1H), 7.38 (dd, $^3J_{HH}$ = 9.5 Hz, $^4J_{HH}$ = 1.7, C$\underline{H}_{(7)}$, 1H), 7.43 (s, C$\underline{H}_{(2)}$, 1H), 7.57 (bd, $^3J_{HH}$ = 9.5 Hz, C$\underline{H}_{(8)}$, 1H), 7.66 (d, $^3J_{HH}$ = 15.9 Hz, (CH$_3$CH$_2$OC(O)CH=C$\underline{H}$, 1H), 8.15 (bs, C$\underline{H}_{(5)}$, 1H), **^{31}P NMR** (284 MHz, CDCl$_3$) δ: 21.33, **^{13}C NMR** (176 MHz, CDCl$_3$) δ: 14.42 (s, $\underline{C}$H$_3$CH$_2$OC(O)CH=CH, 1C),16.47-16.62 (m, $\underline{C}$H$_3$CH$_2$OP, 2C), 21.60 (d, $^2J_{PC}$ = 3.4 Hz, $\underline{C}$H$_2$CHP, 1C), 27.90 (s, C($\underline{C}$H$_3$)$_3$, 3C), 45.41 (d, $^1J_{PC}$ = 129.2 Hz, CH$_2\underline{C}$HP, 1C), 60.78 (s, CH$_3\underline{C}$H$_2$OC(O)CH=CH, 1C), 63.11 (d, $^2J_{PC}$ = 6.9 Hz, CH$_3\underline{C}$H$_2$OP, 1C), 63.22 (d, $^2J_{PC}$ = 6.4 Hz, CH$_3\underline{C}$H$_2$OP, 1C), 82.83 (s, $\underline{C}$(CH$_3$)$_3$, 1C), 118.37 (s, $\underline{C}$H$_{(8)}$, 1C), 118.92 (s, CH$_3$CH$_2$OC(O)$\underline{C}$H=CH, 1C), 120.84 (s, $\underline{C}_{(6)}$, 1C), 121.22 (s, $\underline{CH}_{(7)}$, 1C), 122.58 (d, $^3J_{PC}$ = 19.1 Hz, $\underline{C}_{(3)}$, 1C), 125.13 (s, $\underline{C}$H$_{(5)}$, 1C), 132.78 (s, $\underline{C}$H$_{(2)}$, 1C), 140.64 (s, CH$_3$CH$_2$OC(O)CH=$\underline{C}$H, 1C), 145.45 (s, C$_{(9)}$, 1C), 166.59 (s, CH$_3$CH$_2$O$\underline{C}$(O)CH=CH, 1C), 167.32 (d, $^2J_{PC}$ = 5.1 Hz, $\underline{C}$O$_2$*t*-Bu, 1C).

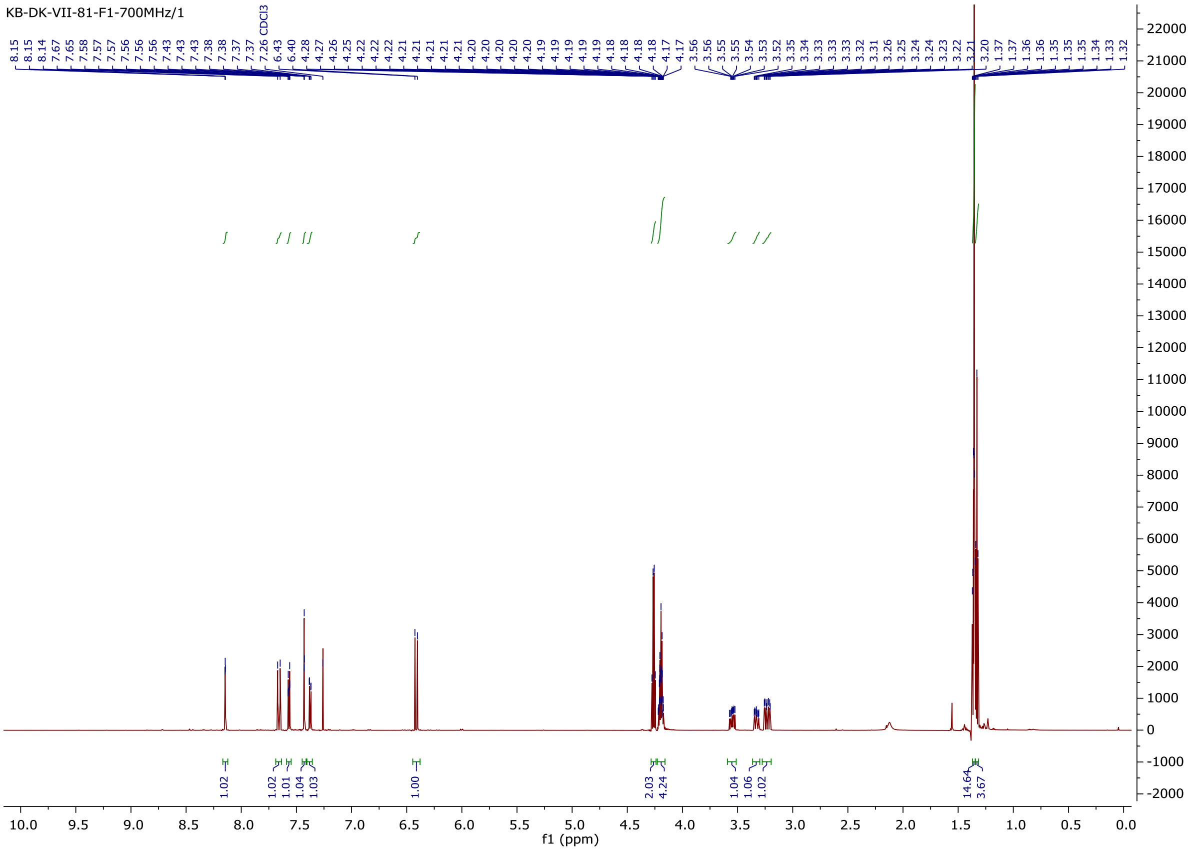

Figure S44. ^{1}H NMR of compound **19** (700 MHz, $CDCl_3$)

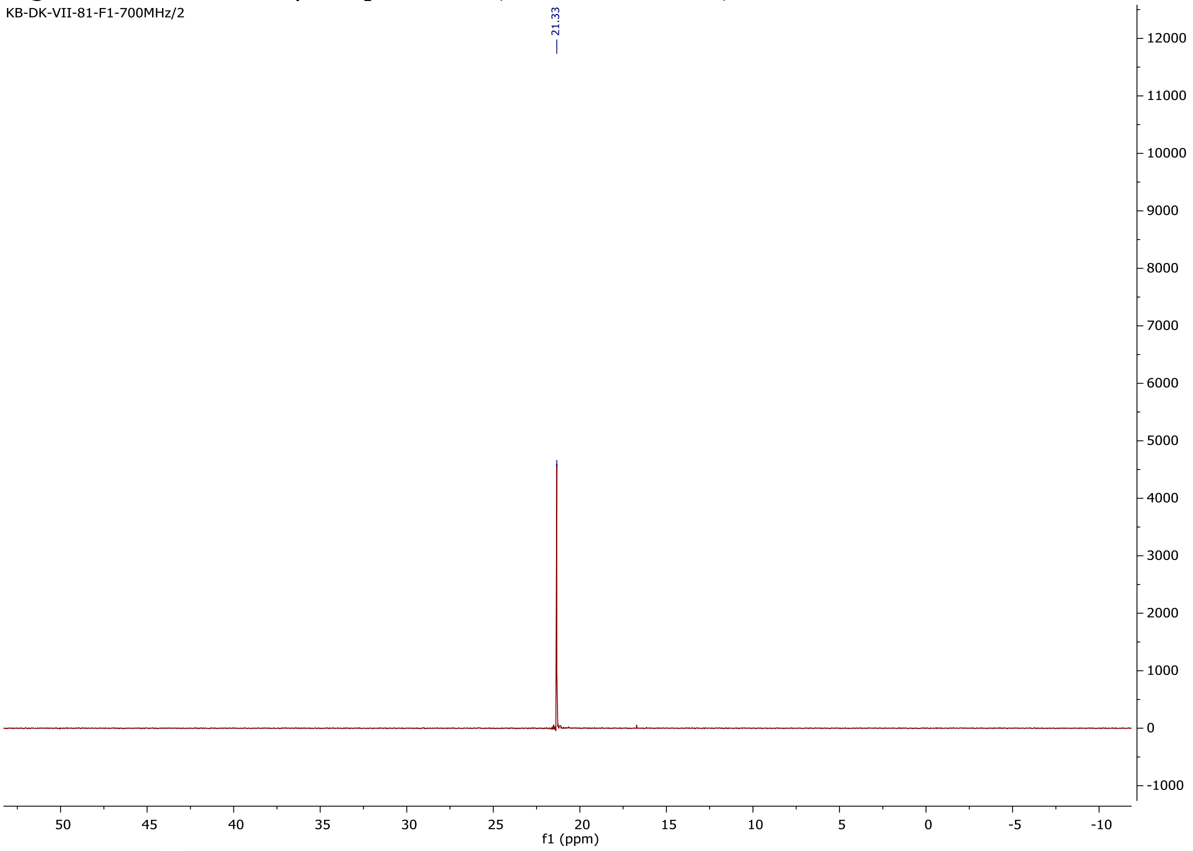

Figure S45. ^{31}P NMR of compound **19** (284 MHz, $CDCl_3$)

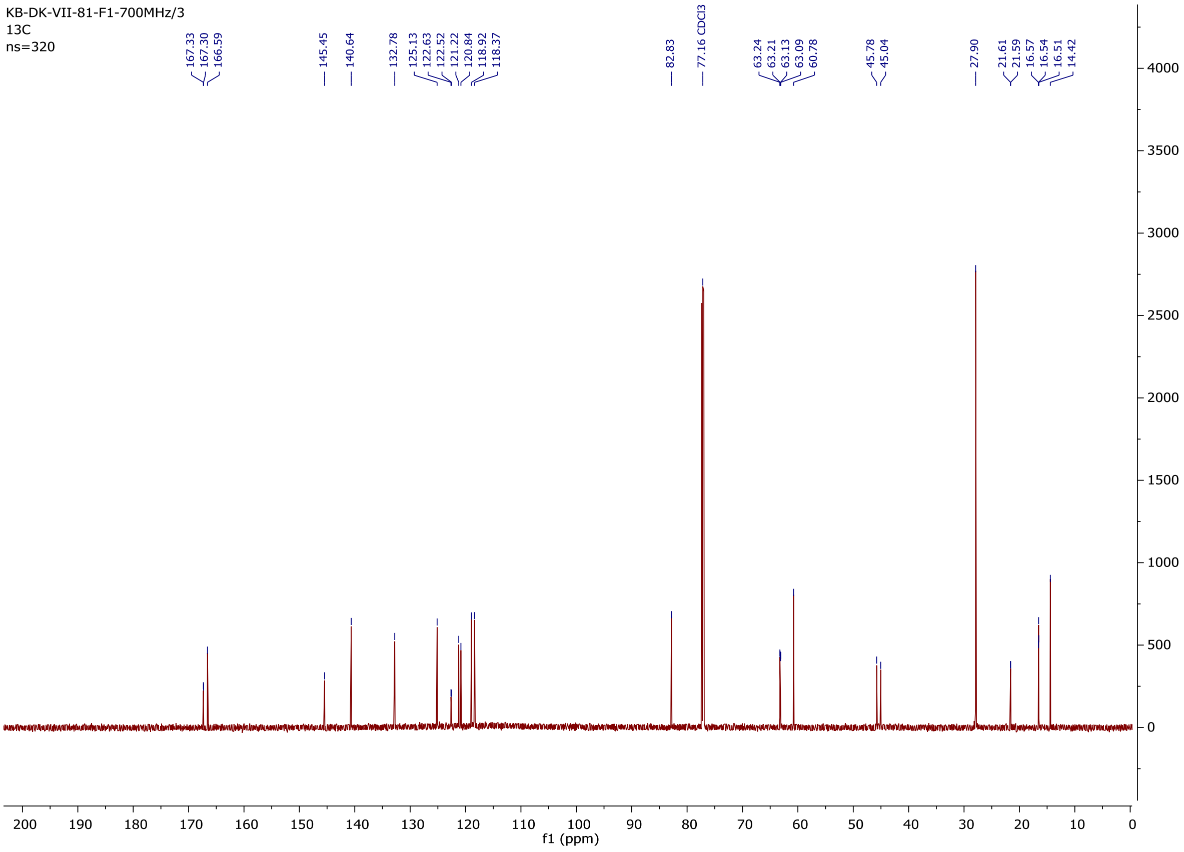

Figure S46. ^{13}*C NMR of compound* ***19*** *(176 MHz, CDCl*$_3$*)*

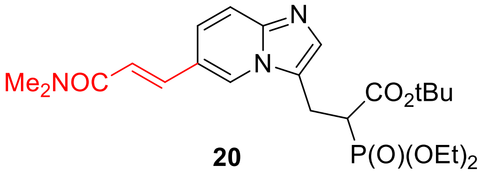

(*E*)-*tert*-Butyl 2-(diethoxyphosphoryl)-3-(6-(3-(dimethylamino)-3-oxoprop-1-en-1-yl)imidazo[1,2-*a*]pyridin-3-yl)propanoate (20). Yield 69%

HRMS m/z: calculated 480.2258 ($C_{23}H_{34}N_3O_6P + H)^+$, found 480.2261 ($C_{23}H_{34}N_3O_6P + H)^+$, **^{1}H NMR** (700 MHz, $CDCl_3$) δ: 1.30-1.38 (m, (C<u>H</u>$_3$CH$_2$O)$_2$(O)P, C(C<u>H</u>$_3$)$_3$, 15H), 3.09 (s, NC<u>H</u>$_3$, 3H), 3.20 (s, NC<u>H</u>$_3$, 3H), 3.26 (ddd, $^2J_{PH}$ = 22.8, $^3J_{HH}$ = 11.6, $^3J_{HH}$ = 3.1, CH$_2$C<u>H</u>P, 1H), 3.34 (ddd, $^2J_{HH}$ = 16.8, $^3J_{PH}$ = 9.8, $^3J_{HH}$ = 3.1, C<u>H</u>$_2$CHP, 1H), 3.57 (ddd, $^2J_{HH}$ = 16.8, $^3J_{HH}$ = 11.6, $^3J_{PH}$ = 6.9, C<u>H</u>$_2$CHP, 1H), 4.16 – 4.26 (m, (CH$_3$C<u>H</u>$_2$O)$_2$P, 4H), 6.91 (d, $^3J_{HH}$ = 15.3 Hz, (CH$_3$)$_2$NC(O)C<u>H</u>=CH, 1H), 7.41 (dd, $^3J_{HH}$ = 9.4 Hz, $^4J_{HH}$ = 1.7, C<u>H</u>$_{(7)}$, 1H), 7.44 (s, C<u>H</u>$_{(2)}$, 1H), 7.58 (d, $^3J_{HH}$ = 9.4 Hz, C<u>H</u>$_{(8)}$, 1H), 7.67 (d, $^3J_{HH}$ = 15.3 Hz, (CH$_3$)$_2$NC(O)CH=C<u>H</u>, 1H), 8.16 (bs, C<u>H</u>$_{(5)}$, 1H), **^{31}P NMR** (283 MHz, CDCl$_3$) δ: 21.40, **^{13}C NMR** (176 MHz, CDCl$_3$) δ: 16.40-16.57 (m, <u>C</u>H$_3$CH$_2$OP, 2C), 21.61 (d, $^2J_{PC}$ = 3.6 Hz, <u>C</u>H$_2$CHP, 1C), 27.85 (s, C(<u>C</u>H$_3$)$_3$, 3C), 36.03 (s, N<u>C</u>H$_3$, 1C), 37.49 (s, N<u>C</u>H$_3$, 1C), 45.27 (d, $^1J_{PC}$ = 129.1 Hz, CH$_2$<u>C</u>HP, 1C), 63.02 (d, $^2J_{PC}$ = 6.9 Hz, CH$_3$<u>C</u>H$_2$OP, 1C), 63.15 (d, $^2J_{PC}$ = 6.4 Hz, CH$_3$<u>C</u>H$_2$OP, 1C), 82.70 (s, <u>C</u>(CH$_3$)$_3$, 1C), 117.91 (s, (CH$_3$)$_2$NC(O)<u>C</u>H=CH, 1C), 118.07 (s, <u>C</u>H$_{(8)}$, 1C), 121.48 (s, <u>C</u>$_{(6)}$, 1C), 121.54 (s, <u>C</u>H$_{(7)}$, 1C), 122.40 (d, $^3J_{PC}$ = 19.0 Hz, <u>C</u>$_{(3)}$, 1C), 124.39 (s, <u>C</u>H$_{(5)}$, 1C), 132.51 (s, <u>C</u>H$_{(2)}$, 1C), 138.43 (s, (CH$_3$)$_2$NC(O)CH=<u>C</u>H, 1C), 145.32 (s, C$_{(9)}$, 1C), 166.19 (s, N(O)<u>C</u>CH=CH, 1C), 167.25 (d, $^2J_{PC}$ = 5.1 Hz, <u>C</u>O$_2$*t*-Bu, 1C).

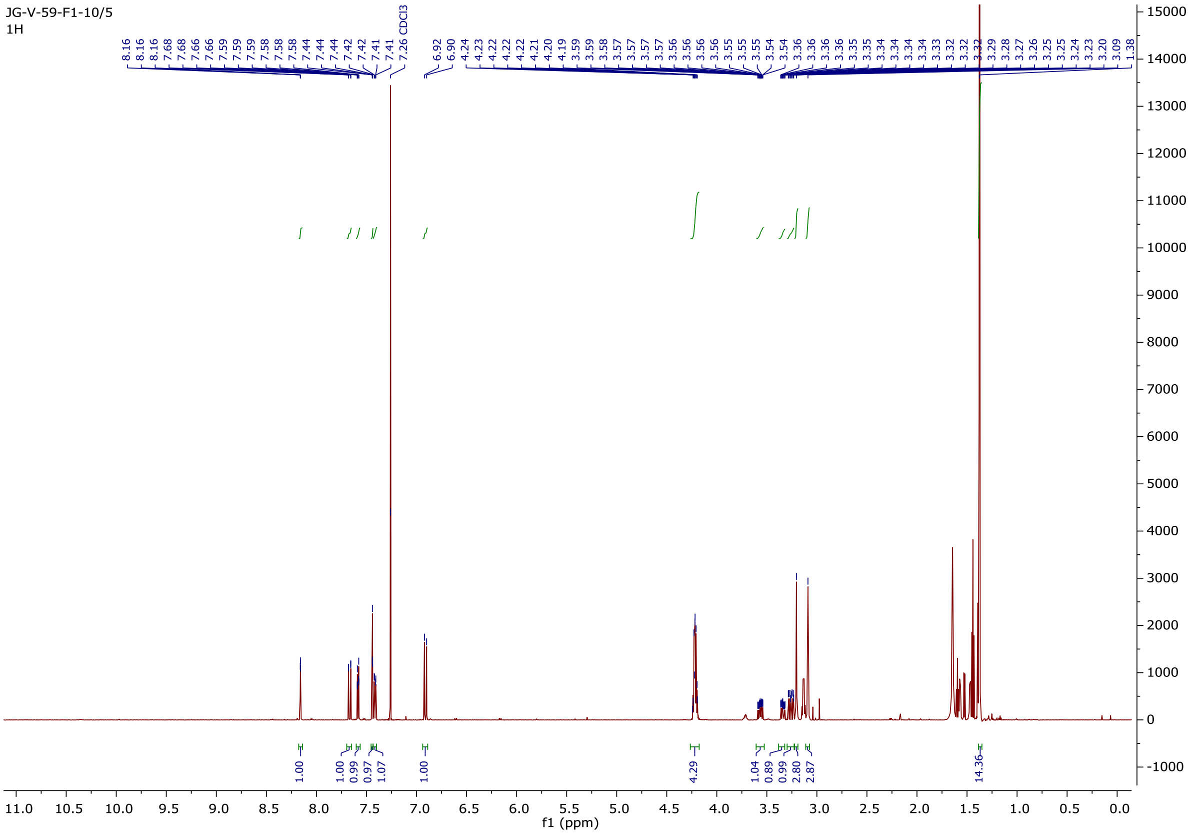

*Figure S47. ^{1}H NMR of compound **20** (700 MHz, $CDCl_3$)*

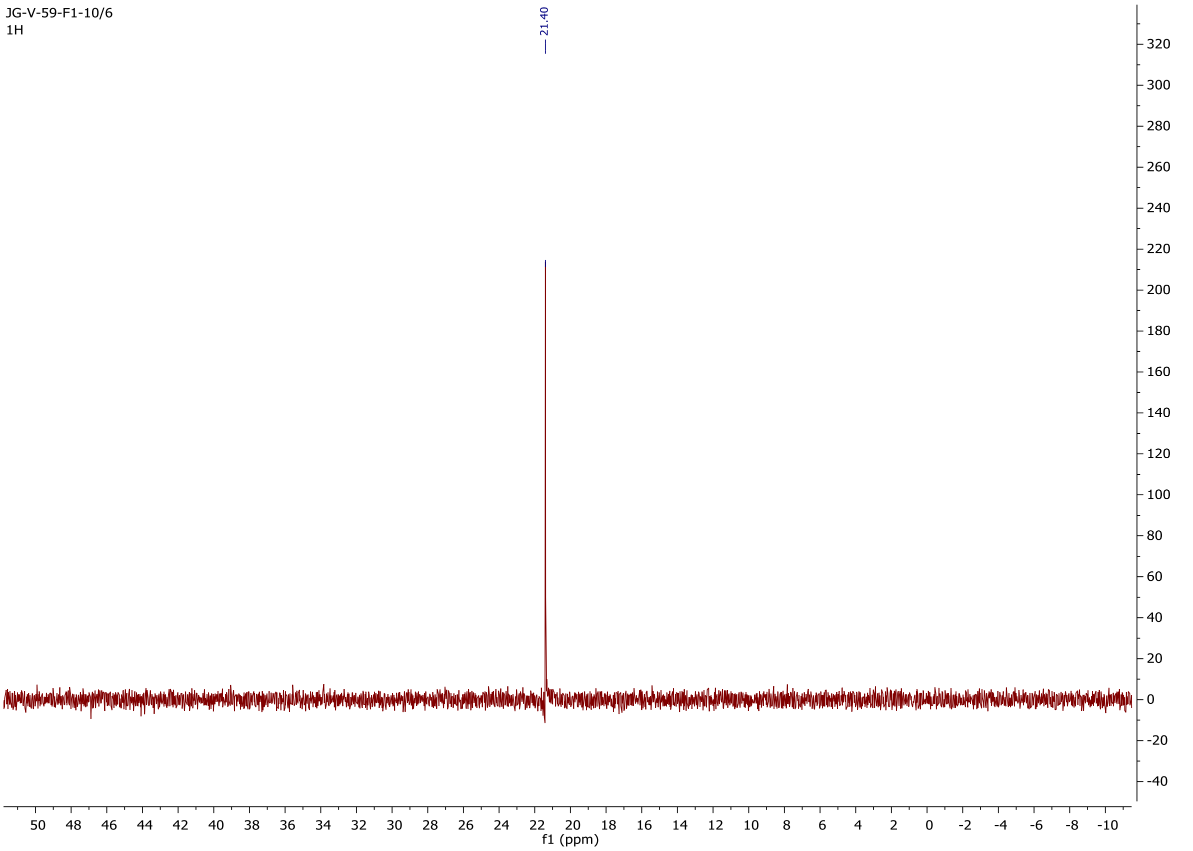

*Figure S48. ^{31}P NMR of compound **20** (283 MHz, $CDCl_3$)*

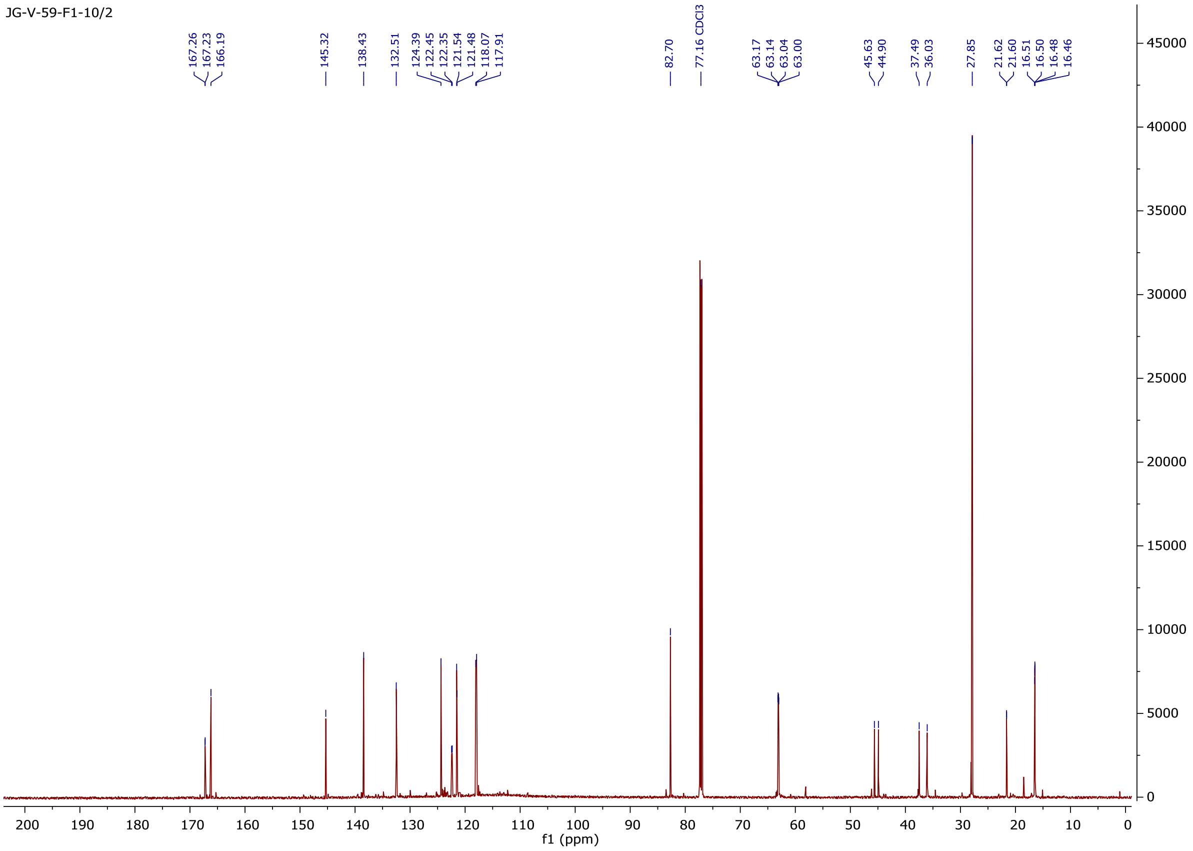

*Figure S49. ^{13}C NMR of compound **20** (176 MHz, $CDCl_3$)*

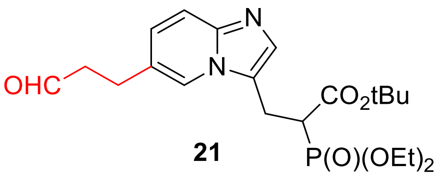

21

***tert*-Butyl 2-(diethoxyphosphoryl)-3-(6-(3-oxopropyl)imidazo[1,2-*a*]pyridin-3-yl)propanoate (21).** Yield 60%.

HRMS m/z: calculated 439.1992 ($C_{21}H_{31}N_2O_6P$ + H)$^+$, found 439.2006 ($C_{21}H_{31}N_2O_6P$ + H)$^+$, **^{1}H NMR** (700 MHz, $CDCl_3$) δ: 1.25-1.42 (m, C(C$\underline{H}_3$)$_3$, (C$\underline{H}_3$CH$_2$O)$_2$P, 15H), 2.76-2.90 (m, CHOC$\underline{H}_2$CH$_2$, 2H), 2.94-3.04 (m, CHOCH$_2$C$\underline{H}_2$, 2H), 3.12-3.41 (m, C$\underline{H}_2$CHP, CH$_2$C$\underline{H}$P, 2H), 3.44-3.65 (m, C$\underline{H}_2$CHP, 1H), 4.08 – 4.29 (m, (CH$_3$C$\underline{H}_2$O)$_2$P, 4H), 7.19 (dd, $^3J_{HH}$ = 9.3 Hz, $^4J_{HH}$ = 1.7 Hz, C$\underline{H}_{(7)}$, 1H), 7.43 (s, C$\underline{H}_{(2)}$, 1H), 7.71 (d, $^3J_{HH}$ = 9.3 Hz, $^5J_{HH}$ = 0.9 Hz C$\underline{H}_{(8)}$, 1H), 7.97 (bs, C$\underline{H}_{(5)}$, 1H), 9.82 (t, $^3J_{HH}$ = 0.9 Hz, C$\underline{H}$O, 1H), **^{31}P NMR** (283 MHz, $CDCl_3$) δ: 21.87

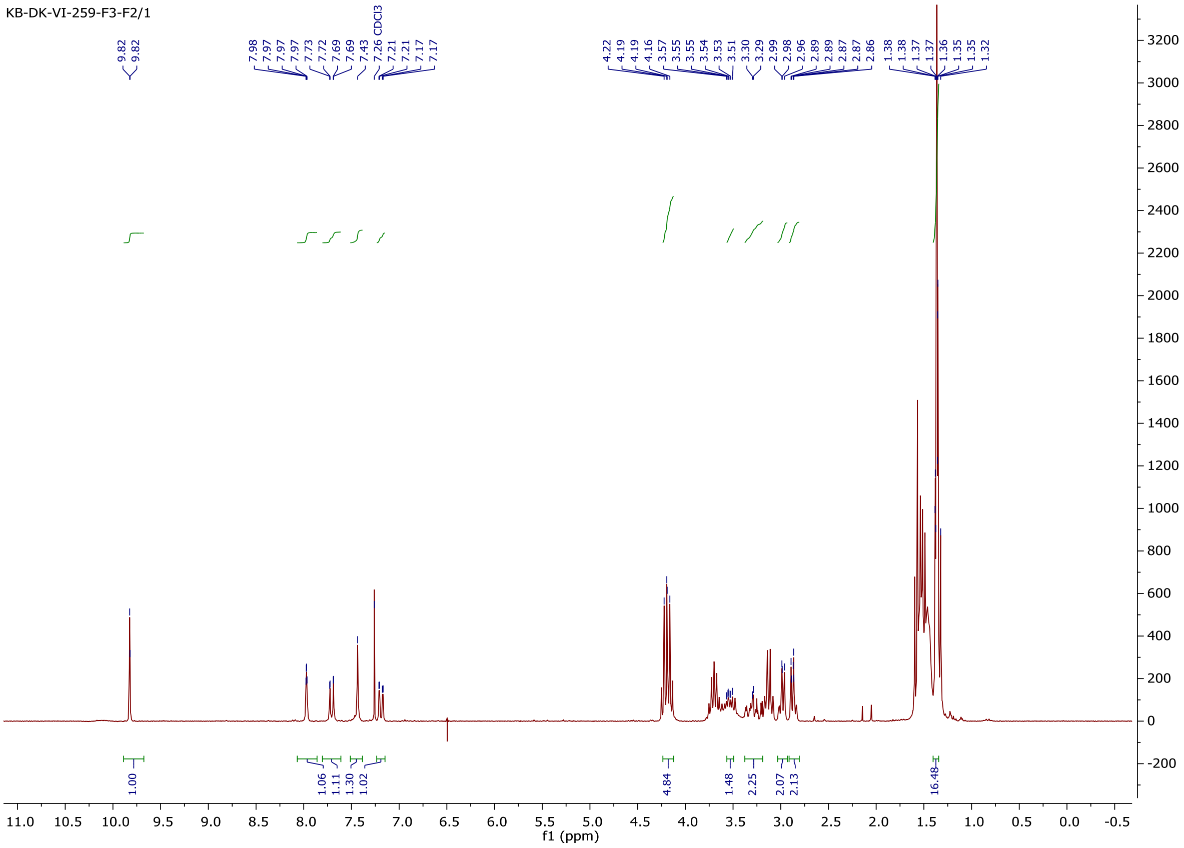

Figure S50. [1]*H NMR of compound* ***21*** *(residual DIPEAxHBr at 1.5, 3.1, 3.6 ppm) (700 MHz, $CDCl_3$)*

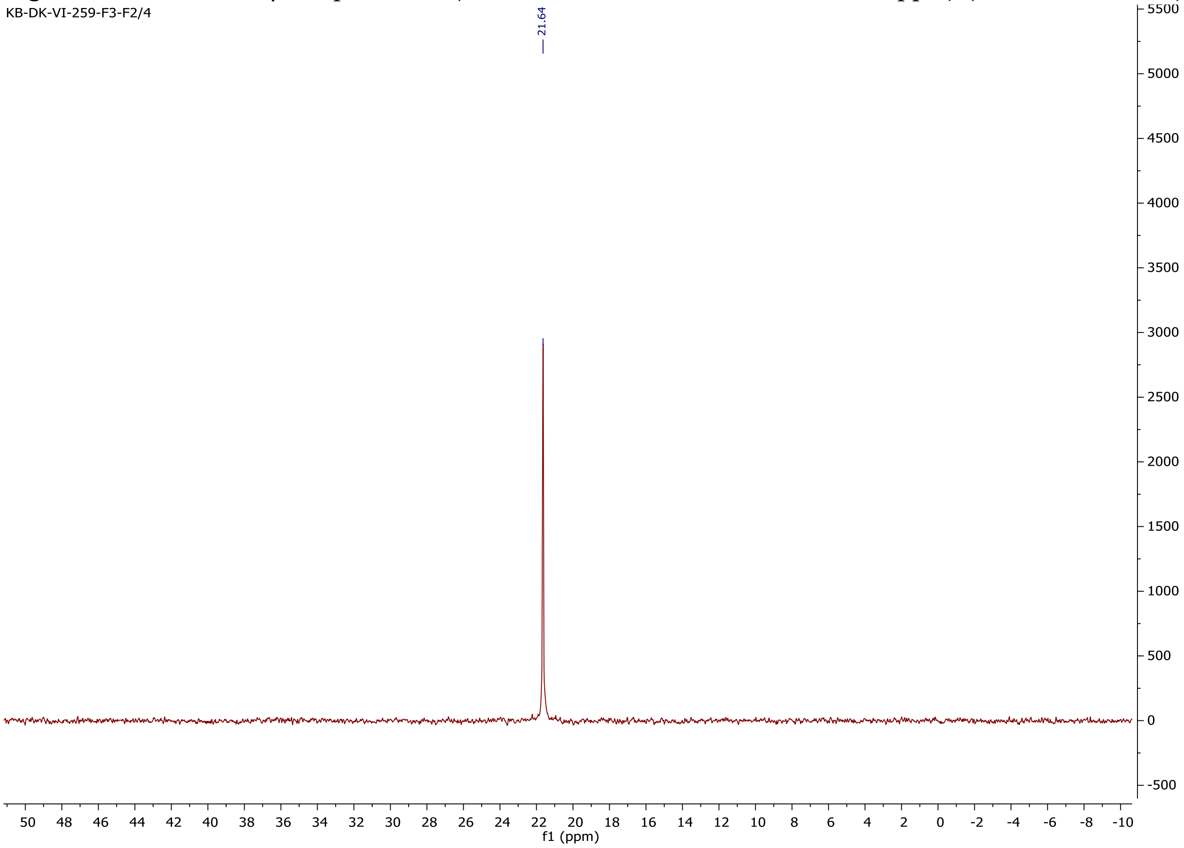

Figure S51. [31]*P NMR of compound* ***21*** *(283 MHz, $CDCl_3$)*

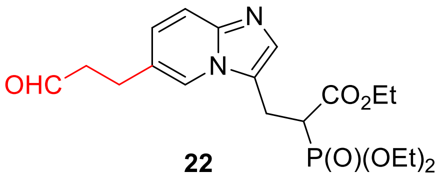

22

Ethyl 2-(diethoxyphosphoryl)-3-(6-(3-oxopropyl)imidazo[1,2-*a*]pyridin-3-yl)propanoate (22). Yield 41%.

^{1}H NMR (700 MHz, $CDCl_3$) δ: 1.17 (t, $^{3}J_{HH}$ = 7.1 Hz, C<u>H</u>$_3$CH$_2$OC, 3H), 1.33 (t, $^{3}J_{HH}$ = 7.1 Hz, (C<u>H</u>$_3$CH$_2$O)$_2$P, 3H), 1.34 (t, $^{3}J_{HH}$ = 7.1 Hz, (C<u>H</u>$_3$CH$_2$O)$_2$P, 6H), 2.84 (t, CHOC<u>H</u>$_2$CH$_2$, $^{3}J_{HH}$ = 7.2 Hz, 2H), 2.96 (t, CHOCH$_2$C<u>H</u>$_2$, $^{3}J_{HH}$ = 7.2 Hz, 2H), 3.27-3.36 (m, C<u>H</u>$_2$CHP, CH$_2$C<u>H</u>P, 2H), 3.56 (ddd, $^{2}J_{HH}$ = 16.5, $^{3}J_{HH}$ = 11.6, $^{3}J_{PH}$ = 7.2, C<u>H</u>$_2$CHP, 1H), 4.00 – 4.20 (m, (CH$_3$C<u>H</u>$_2$O)$_2$P, CH$_3$C<u>H</u>$_2$OC, 6H), 7.11 (dd, $^{3}J_{HH}$ = 9.1 Hz, $^{4}J_{HH}$ = 1.6 Hz, C<u>H</u>$_{(7)}$, 1H), 7.39 (s, C<u>H</u>$_{(2)}$, 1H), 7.60 (d, $^{3}J_{HH}$ = 9.1 Hz, C<u>H</u>$_{(8)}$, 1H), 7.91 (bs, C<u>H</u>$_{(5)}$, 1H), 9.81 (bs, C<u>H</u>O, 1H), **^{31}P NMR** (283 MHz, $CDCl_3$) δ: 20.72, **^{13}C NMR** (176 MHz, $CDCl_3$) δ: 14.05 (s, <u>C</u>H$_3$CH$_2$OC, 1C), 16.41 (d, $^{3}J_{PC}$ = 6.1 Hz, <u>C</u>H$_3$CH$_2$OP, 1C), 16.45 (d, $^{3}J_{PC}$ =5.7 Hz, <u>C</u>H$_3$CH$_2$OP, 1C), 21.53 (d, $^{2}J_{PC}$ = 3.7 Hz, <u>C</u>H$_2$CHP, 1C), 25.06 (s, CHOCH$_2$<u>C</u>H$_2$, 1C), 44.22 (d, $^{1}J_{PC}$ = 129.5 Hz, CH$_2$<u>C</u>HP, 1C), 44.48 (s, CHO<u>C</u>H$_2$CH$_2$, 1C), 62.02 (s, CH$_3$<u>C</u>H$_2$OC, 1C), 63.27 (d, $^{2}J_{PC}$ = 7.0 Hz, CH$_3$<u>C</u>H$_2$OP, 1C), 63.35 (d, $^{2}J_{PC}$ = 6.3 Hz, CH$_3$<u>C</u>H$_2$OP, 1C), 117.21 (s, <u>C</u>H$_{(8)}$, 1C), 121.45 (d, $^{3}J_{PC}$ = 18.2 Hz, <u>C</u>$_{(3)}$, 121.47 (s, <u>C</u>H$_{(5)}$, 1C), 125.77 (s, <u>C</u>$_{(6)}$, 1C), 126.94 (s, <u>C</u>H$_{(7)}$, 1C), 130.03 (s, <u>C</u>H$_{(2)}$, 1C), 143.93 (s, C$_{(9)}$, 1C), 168.17 (d, $^{2}J_{PC}$ = 5.1 Hz, <u>C</u>O$_2$*Et*, 1C), 200.45 (s, <u>C</u>HO, 1C).

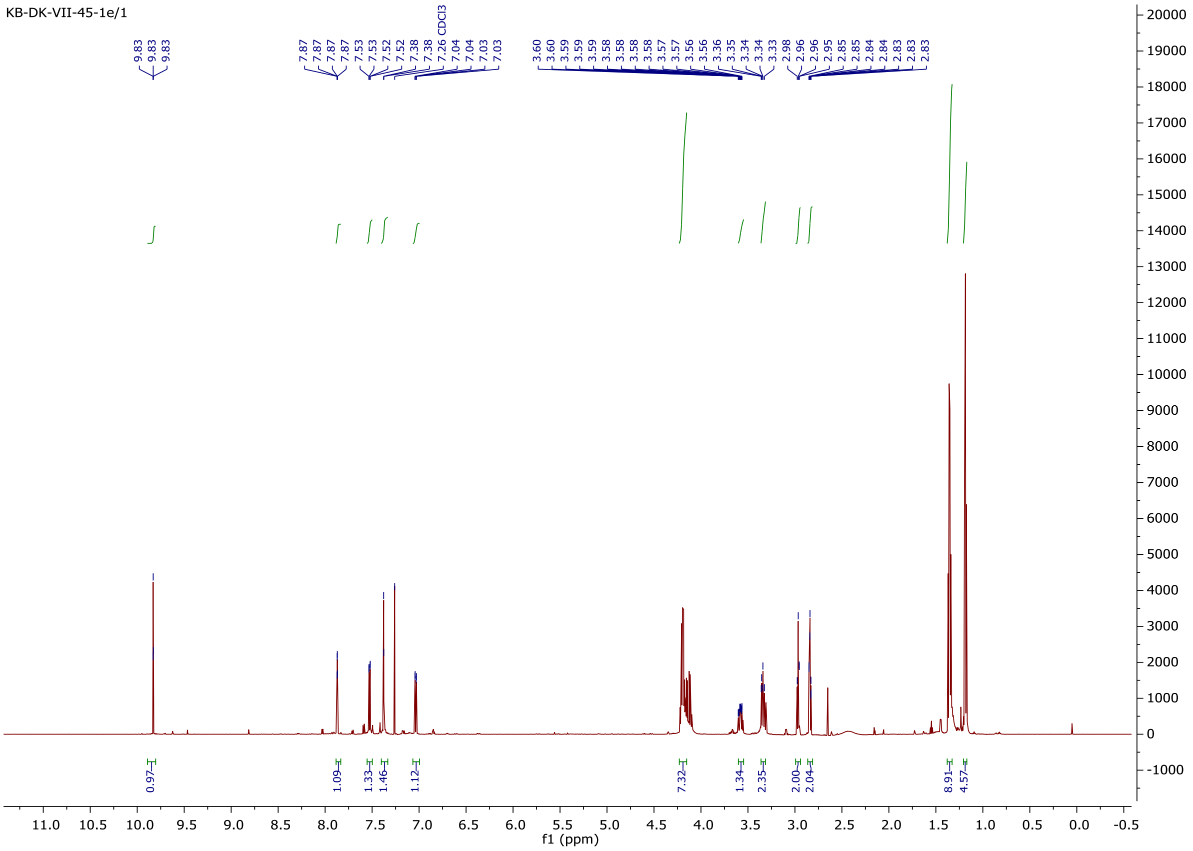

*Figure S52. ^{1}H NMR of compound **22** (700 MHz, $CDCl_3$)*

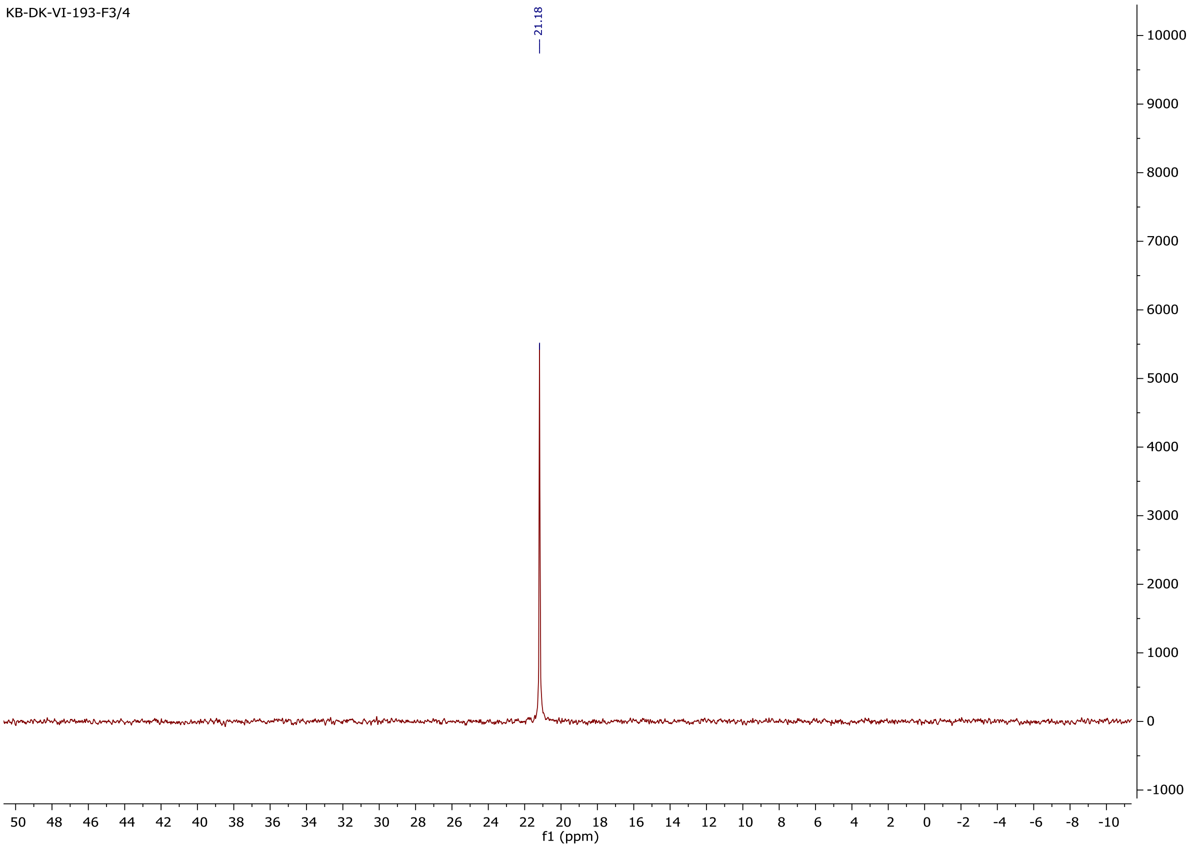

*Figure S53. ^{31}P NMR of compound **22** (283 MHz, $CDCl_3$)*

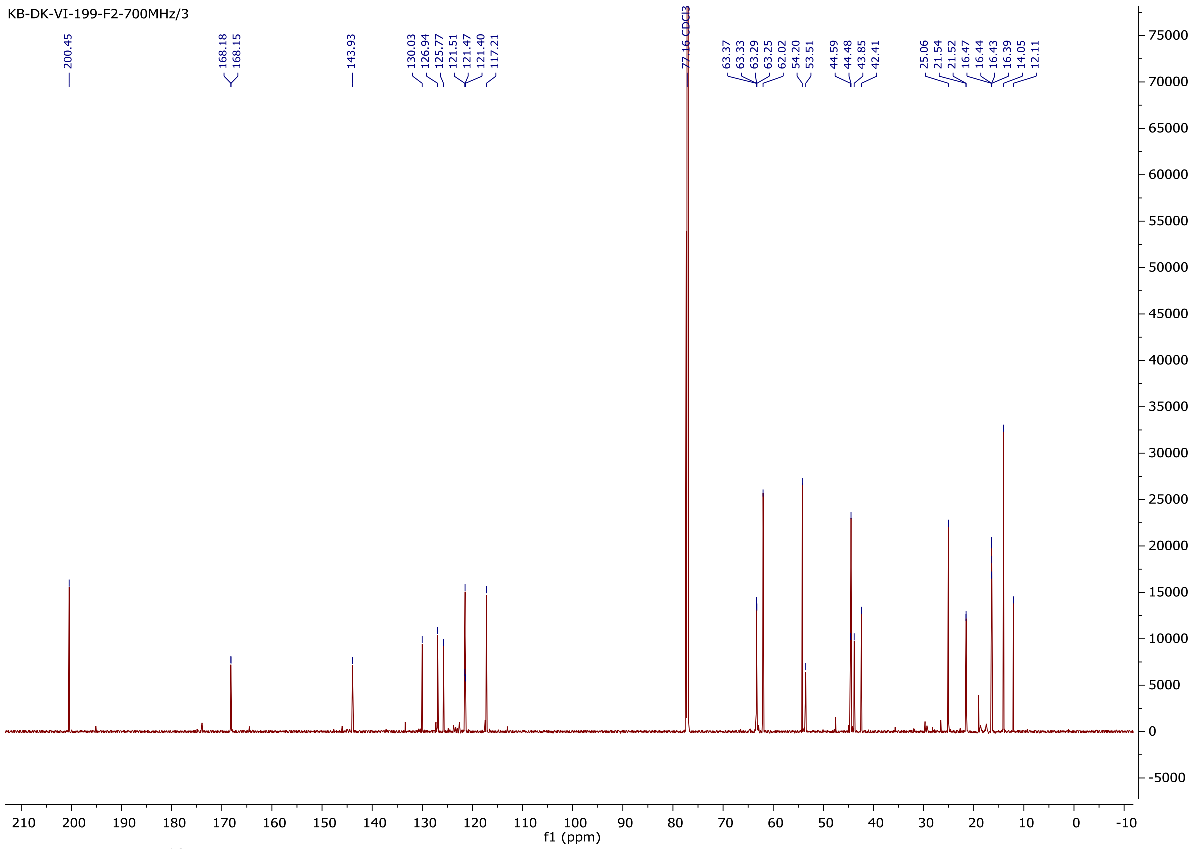

*Figure S54. ^{13}C NMR of compound **22** (176 MHz, CDCl$_3$)*

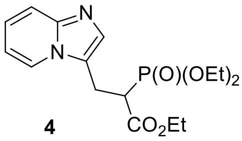

4

Ethyl 2-(diethoxyphosphoryl)-3-(imidazo[1,2-*a*]pyridin-3-yl)propanoate (4).

HRMS m/z: calculated 355.1417 ($C_{16}H_{23}N_2O_5P$ + H)$^+$, found 355.1428 ($C_{16}H_{23}N_2O_5P$ + H)$^+$, **^{1}H NMR** (700 MHz, CDCl$_3$) δ: 1.17 (t, $^3J_{HH}$ = 7.1 Hz, C$\underline{H}_3$CH$_2$OC, 3H), 1.36 (m, (C$\underline{H}_3$CH$_2$O)$_2$P, 6H), 3.22-3.47 (m, C$\underline{H}_2$CHP, CH$_2$C$\underline{H}$P, 2H), 3.53-3.73 (m, C$\underline{H}_2$CHP, 1H), 4.04 – 4.30 (m, (CH$_3$C$\underline{H}_2$O)$_2$P, CH$_3$C$\underline{H}_2$OC, 6H), 6.86 (td, $^3J_{HH}$ = 6.8 Hz, $^4J_{HH}$ = 1.2 Hz, C$\underline{H}_{(6)}$, 1H), 7.18 (ddd, $^3J_{HH}$ = 9.1 Hz, $^3J_{HH}$ = 6.8 Hz, $^4J_{HH}$ = 1.2 Hz, C$\underline{H}_{(7)}$, 1H), 7.43 (s, C$\underline{H}_{(2)}$, 1H),7.60 (dt, $^3J_{HH}$ = 9.1 Hz, $^4J_{HH}$ = 1.2 Hz, C$\underline{H}_{(8)}$, 1H), 8.04 (dt, $^3J_{HH}$ = 6.8 Hz, $^4J_{HH}$ = 1.2 Hz, C$\underline{H}_{(5)}$, 1H), **^{31}P NMR** (283 MHz, CDCl$_3$) δ: 21.29.

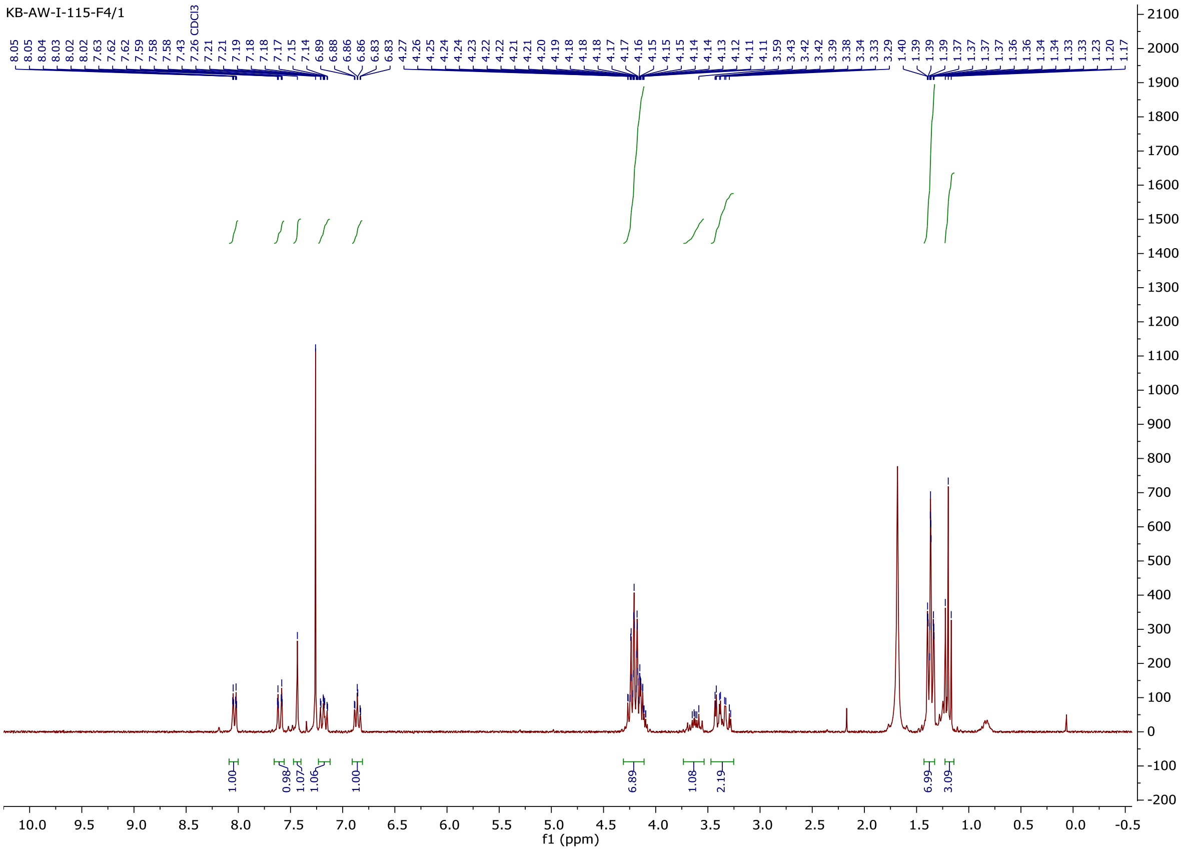

Figure S55. ^{1}H NMR of compound ***4*** *(700 MHz, $CDCl_3$)*

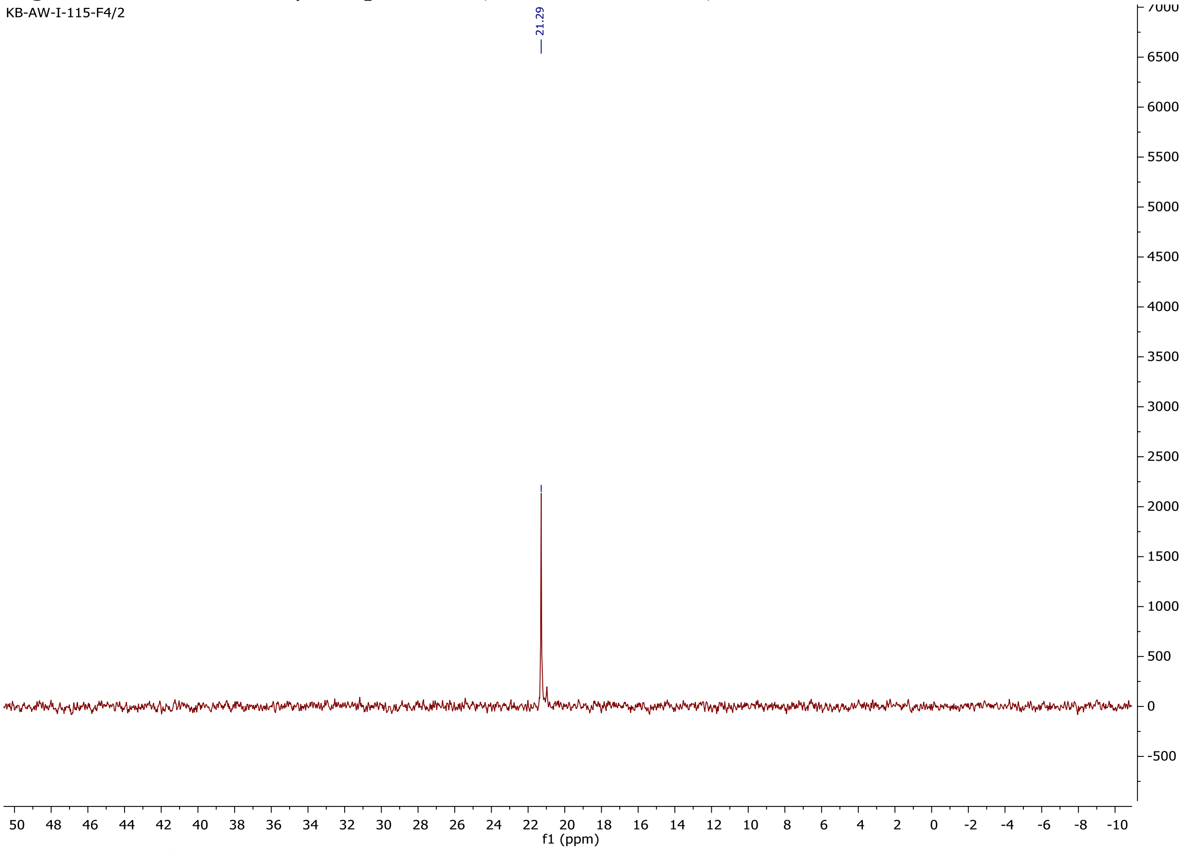

Figure S56. ^{31}P NMR of compound ***4*** *(283 MHz, $CDCl_3$)*

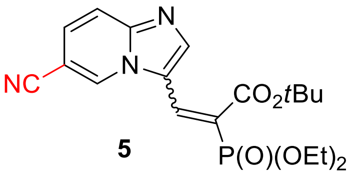

5

(E)-tert-Butyl 3-(6-cyanoimidazo[1,2-a]pyridin-3-yl)-2-(diethoxyphosphoryl)acrylate (5).

HRMS m/z: calculated 406.1526 ($C_{19}H_{24}N_3O_5P$ + H)$^+$, found 406.1528 ($C_{19}H_{24}N_3O_5P$ + H)$^+$, **^{1}H NMR** (700 MHz, $CDCl_3$) δ: 1.38 (t, $^3J_{HH}$ = 7.1 Hz, C<u>H</u>$_3$CH$_2$OP, 6H), 1.57 (s, C(C<u>H</u>$_3$)$_3$, 9H), 4.12 – 4.24 (m, CH$_3$C<u>H</u>$_2$OP, 4H), 7.45 (dd, $^3J_{HH}$ = 9.3 Hz, $^4J_{HH}$ = 1.6 Hz, C<u>H</u>$_{(7)}$, 1H), 7.76 (d, $^3J_{HH}$ = 23.7 Hz, C<u>H</u>=CP, 1H), 7.78 (dd, $^3J_{HH}$ = 9.2 Hz, $^4J_{HH}$ = 1.0 Hz, C<u>H</u>$_{(8)}$, 1H), 8.51 (s, C<u>H</u>$_{(2)}$, 1H), 8.74 (bs, C<u>H</u>$_{(5)}$, 1H), **^{31}P NMR** (283 MHz, $CDCl_3$) δ: 14.93 – *(E)* CH=CP, **^{13}C NMR** (176 MHz, $CDCl_3$) δ: 16.48 (d, $^4J_{PC}$ = 6.8 Hz, <u>C</u>H$_3$CH$_2$OP, 2C), 28.18 (s, C(<u>C</u>H$_3$)$_3$, 3C), 62.86 (d, $^3J_{CP}$ = 5.2 Hz, CH$_3$<u>C</u>H$_2$OP, 2C), 83.41 (s, <u>C</u>(CH$_3$)$_3$, 1C), 100.64 (s, <u>C</u>$_{(6)}$, 1C), 115.92 (s, <u>C</u>N, 1C), 119.62 (s, <u>C</u>H$_{(8)}$, 1C), 121.47 (d, $^3J_{PC}$ = 25.0 Hz, <u>C</u>$_{(3)}$, 1C), 122.72 (d, $^1J_{PC}$ = 180.2 Hz, <u>C</u>P, 1C), 126.49 (s, <u>C</u>H$_{(7)}$, 1C), 129.92 (s, <u>C</u>H$_{(5)}$, 1C), 130.62 (d, $^2J_{PC}$ = 10.0 Hz, <u>C</u>H=CP, 1C), 141.62 (s, <u>C</u>H$_{(2)}$, 1C), 146.61 (s, C$_{(9)}$, 1C), 164.67 (d, $^2J_{PC}$ = 10.4 Hz, <u>C</u>O$_2$t-Bu, 1C).

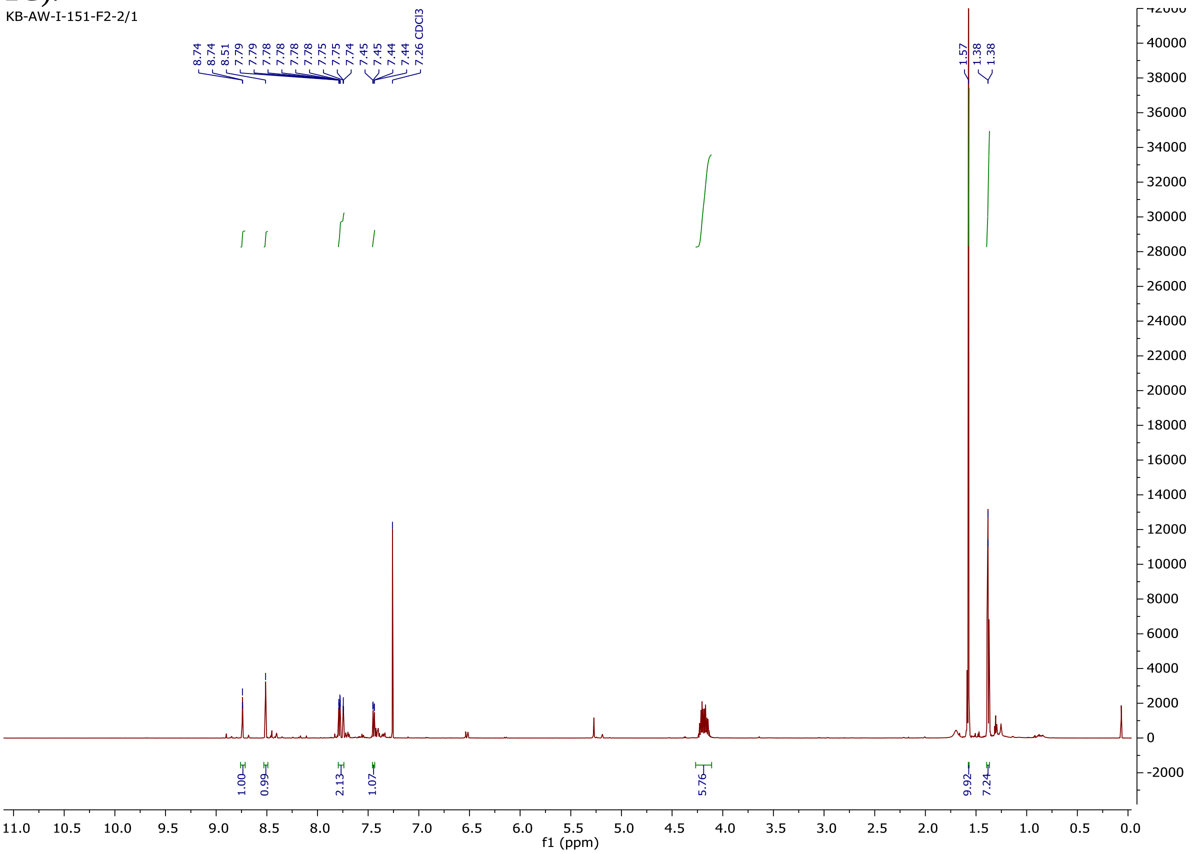

*Figure S57. ^{1}H NMR of compound **5** (700 MHz, $CDCl_3$)*

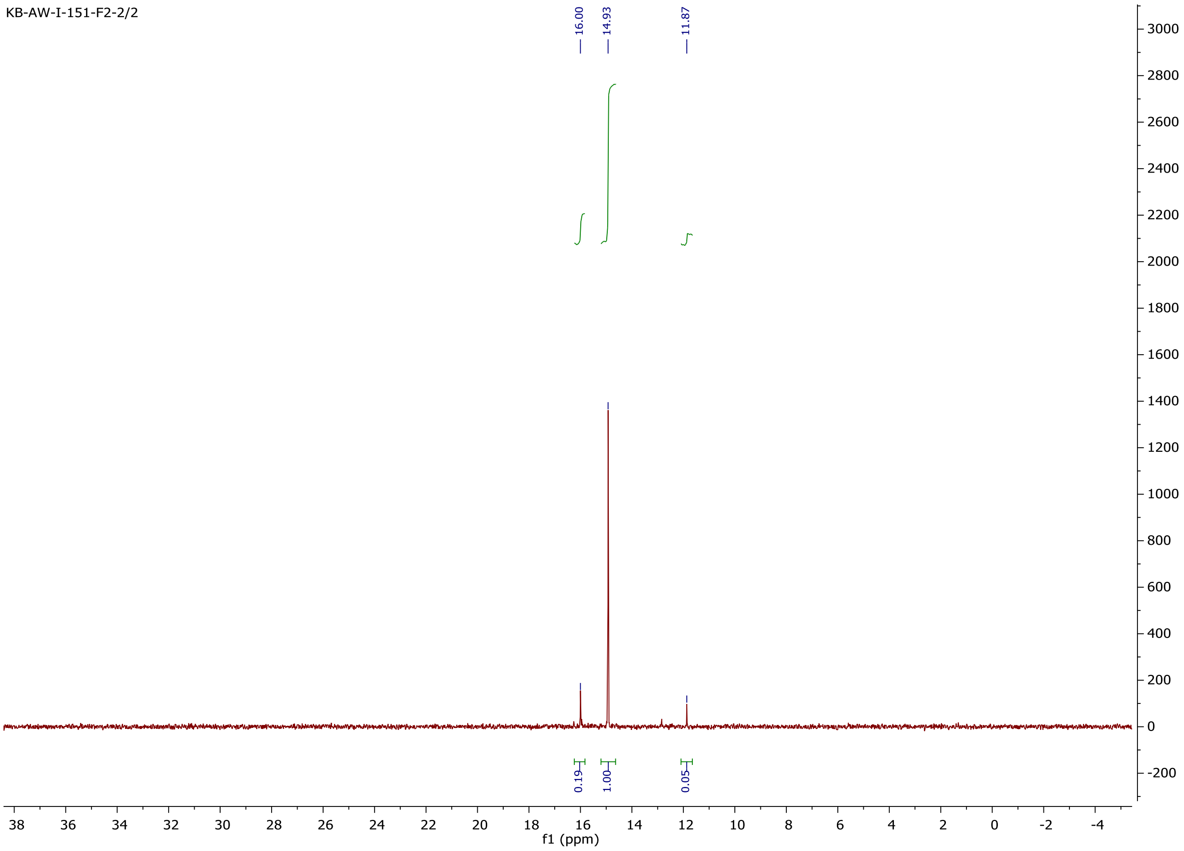

Figure S58. ^{31}P NMR of compound ***5*** *(283 MHz, $CDCl_3$)*

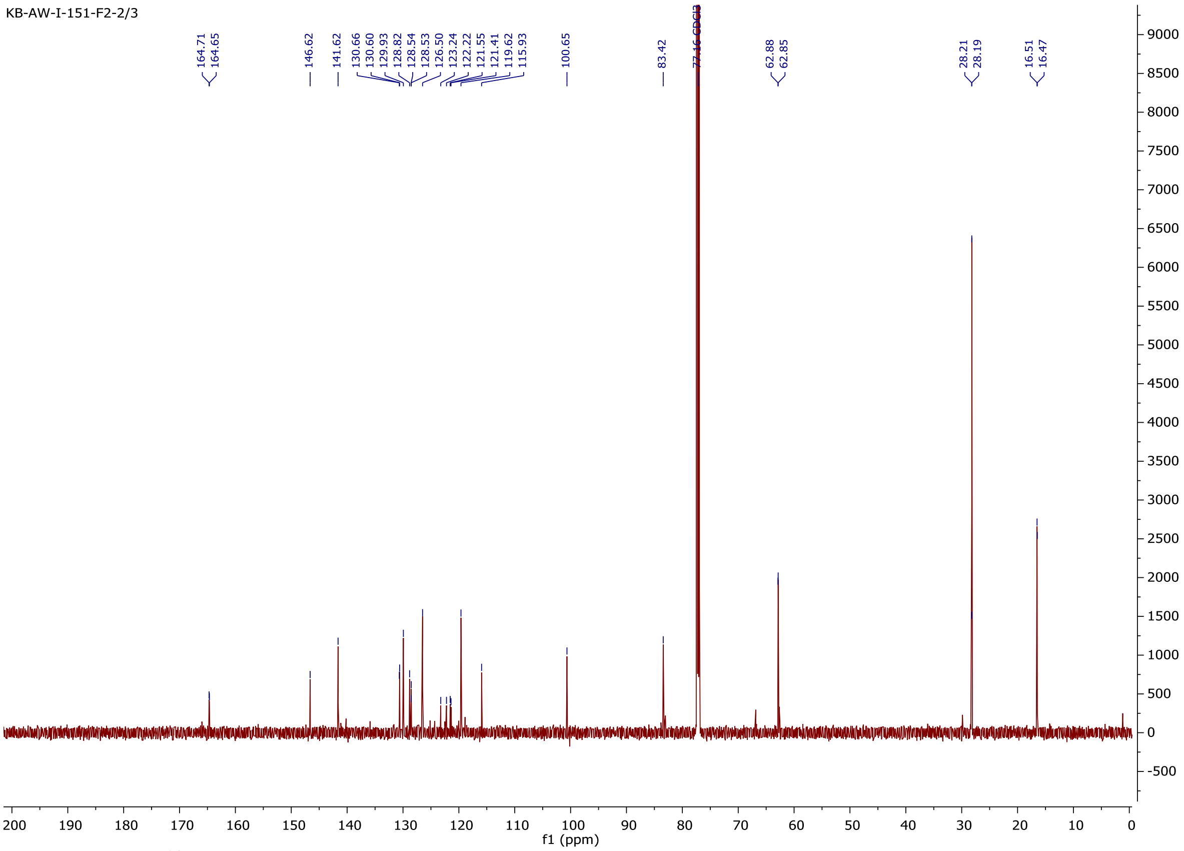

Figure S59. ^{13}C NMR of compound ***5*** *(176 MHz, $CDCl_3$)*\

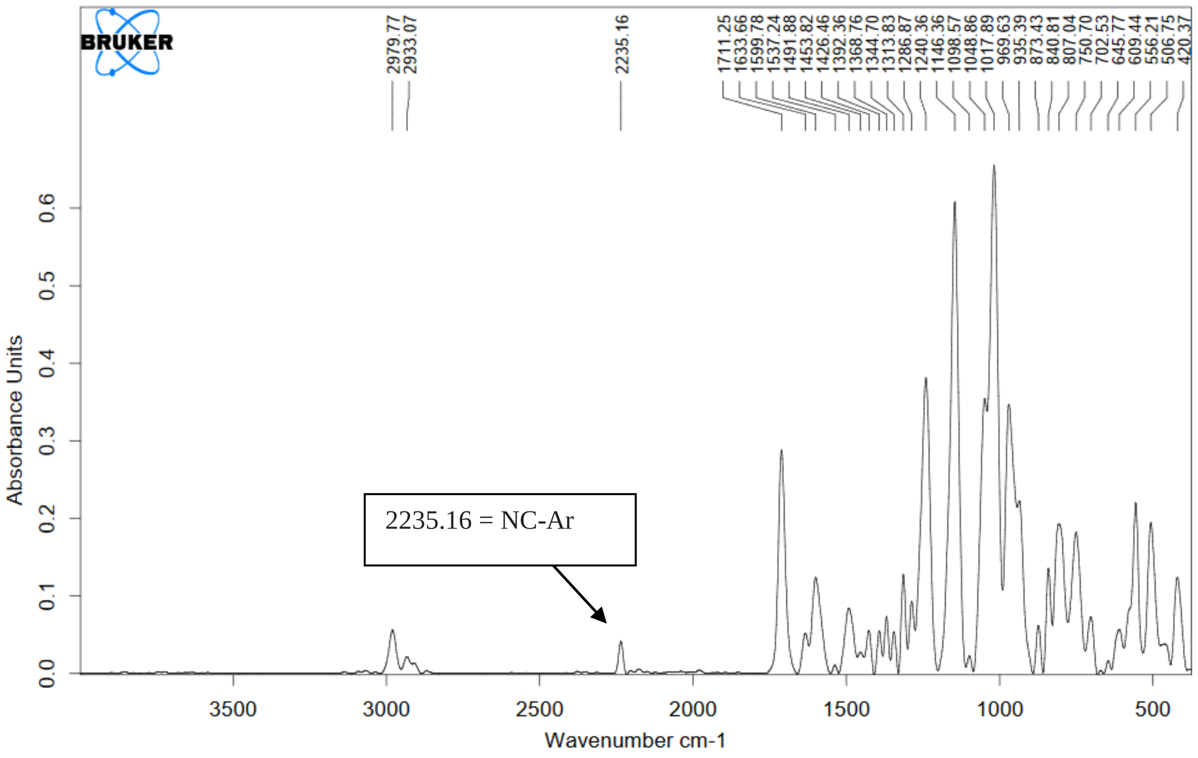

Figure S60. Infrared spectroscopy of ***5***

Side product **5** obtained via different synthetic route [1]

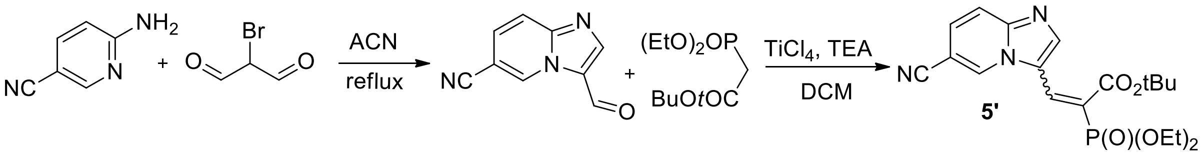

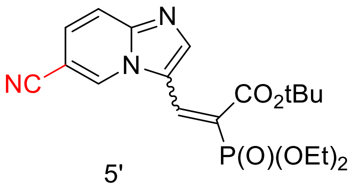

(*E*)-*tert*-Butyl 3-(6-cyanoimidazo[1,2-*a*]pyridin-3-yl)-2-(diethoxyphosphoryl)acrylate (5')

^{1}H NMR (700 MHz, $CDCl_3$) δ: 1.30 (t, $^{3}J_{HH}$ = 7.1 Hz, C<u>H</u>$_3$CH$_2$OP, 6H), 1.50 (s, C(C<u>H</u>$_3$)$_3$, 9H), 4.03 – 4.16 (m, CH$_3$C<u>H</u>$_2$OP, 4H), 7.39 (dd, $^{3}J_{HH}$ = 9.2 Hz, $^{4}J_{HH}$ = 1.6 Hz, C<u>H</u>$_{(7)}$, 1H), 7.69-7.75 (m, C<u>H</u>=CP, C<u>H</u>$_{(8)}$, 2H), 8.42 (s, C<u>H</u>$_{(2)}$, 1H), 8.76 (bs, C<u>H</u>$_{(5)}$, 1H), **^{31}P NMR** (283 MHz, $CDCl_3$) δ: 14.82 – *(E)* CH=CP, **^{13}C NMR** (176 MHz, $CDCl_3$) δ: 16.26 (d, $^{4}J_{PC}$ = 6.8 Hz, <u>C</u>H$_3$CH$_2$OP, 2C), 27.96 (s, C(<u>C</u>H$_3$)$_3$, 3C), 62.62 (d, $^{3}J_{CP}$ = 5.2 Hz, CH$_3$<u>C</u>H$_2$OP, 2C), 83.13 (s, <u>C</u>(CH$_3$)$_3$, 1C), 100.33 (s, <u>C</u>$_{(6)}$, 1C), 115.79 (s, <u>C</u>N, 1C), 119.31 (s, <u>C</u>H$_{(8)}$, 1C), 121.31 (d, $^{3}J_{PC}$ = 25.0 Hz, <u>C</u>$_{(3)}$, 1C), 122.41 (d, $^{1}J_{PC}$ = 180.6 Hz, <u>C</u>P, 1C), 126.35 (s, <u>C</u>H$_{(7)}$, 1C), 130.02

(s, C̲H$_{(5)}$, 1C), 130.47 (d, $^{2}J_{PC}$ = 10.0 Hz, C̲H=CP, 1C), 141.28 (s, C̲H$_{(2)}$, 1C), 146.40 (s, C$_{(9)}$, 1C), 164.51 (d, $^{2}J_{PC}$ = 10.4 Hz, C̲O$_2$*t*-Bu, 1C).

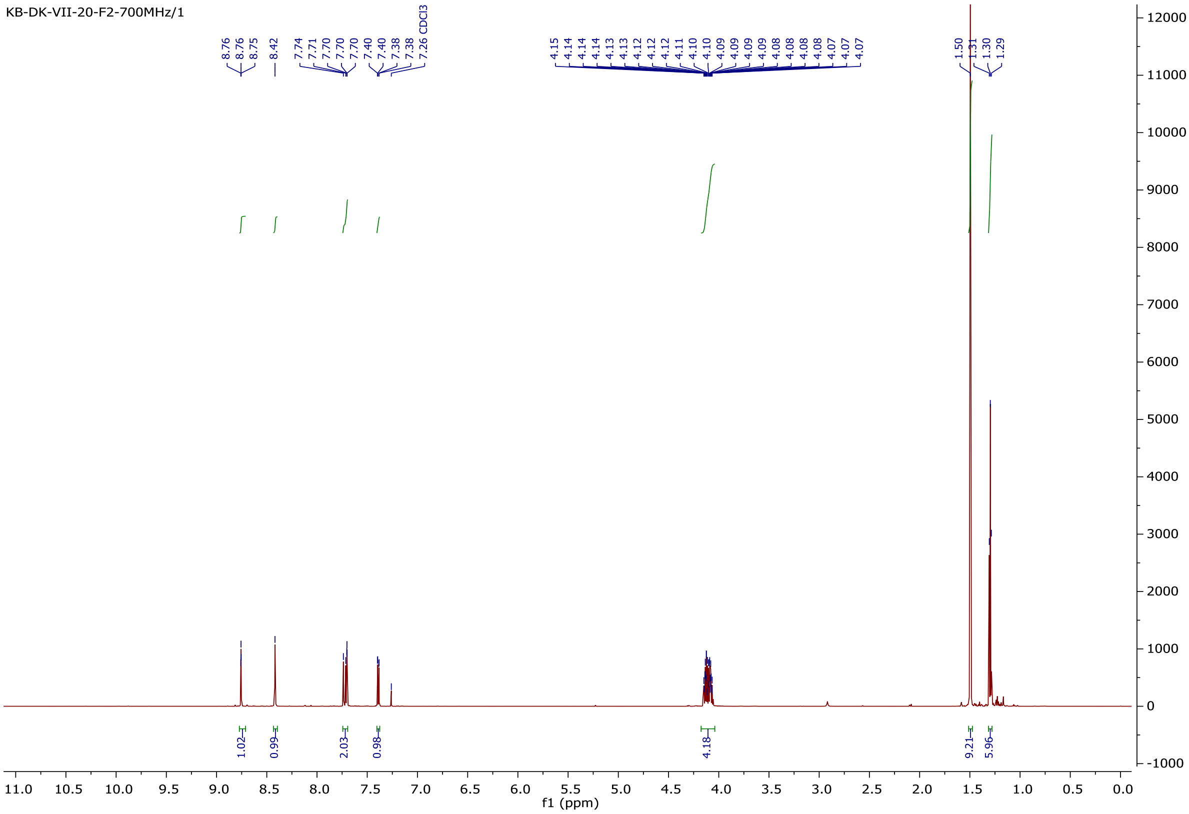

*Figure S61. ^{1}H NMR of compound **5'** (700 MHz, $CDCl_3$)*

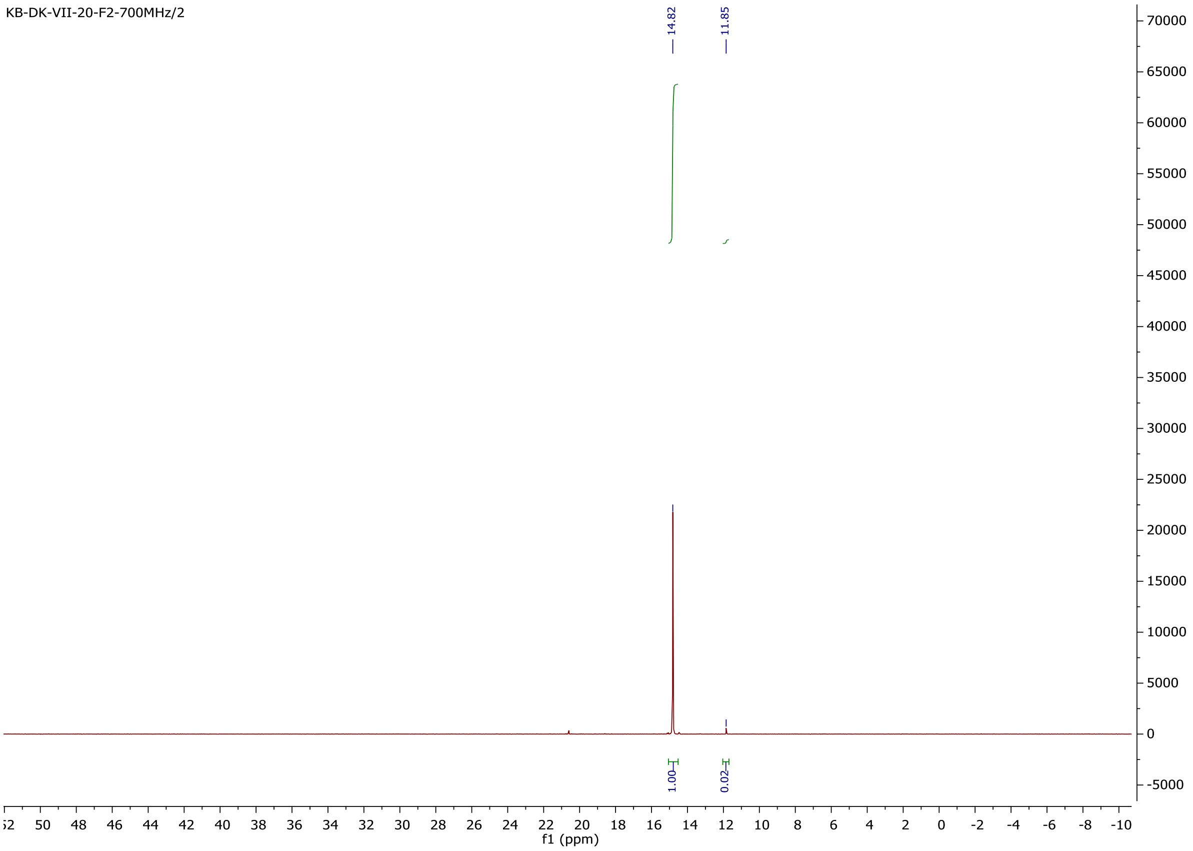

Figure S62. ^{31}P NMR of compound **5’** (283 MHz, $CDCl_3$)

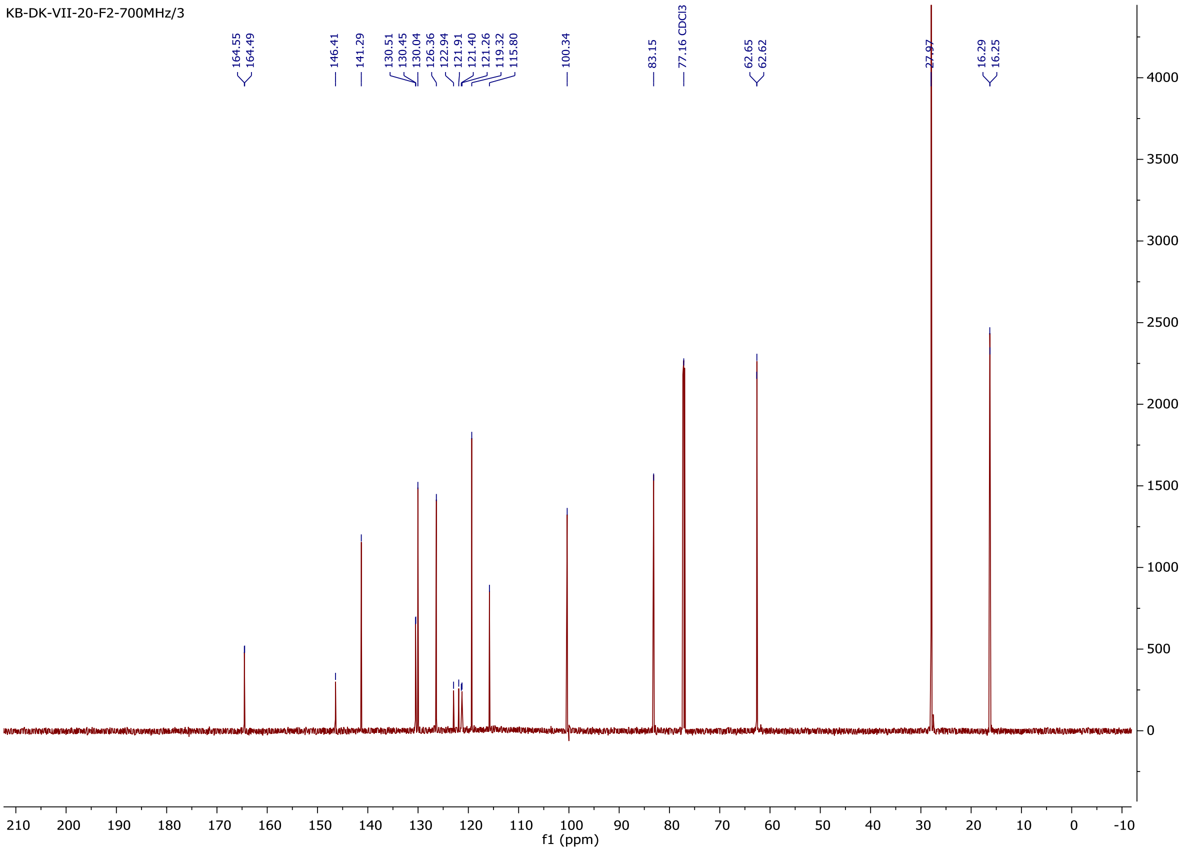

Figure S63. ^{13}C NMR of compound **5’** (176 MHz, $CDCl_3$)

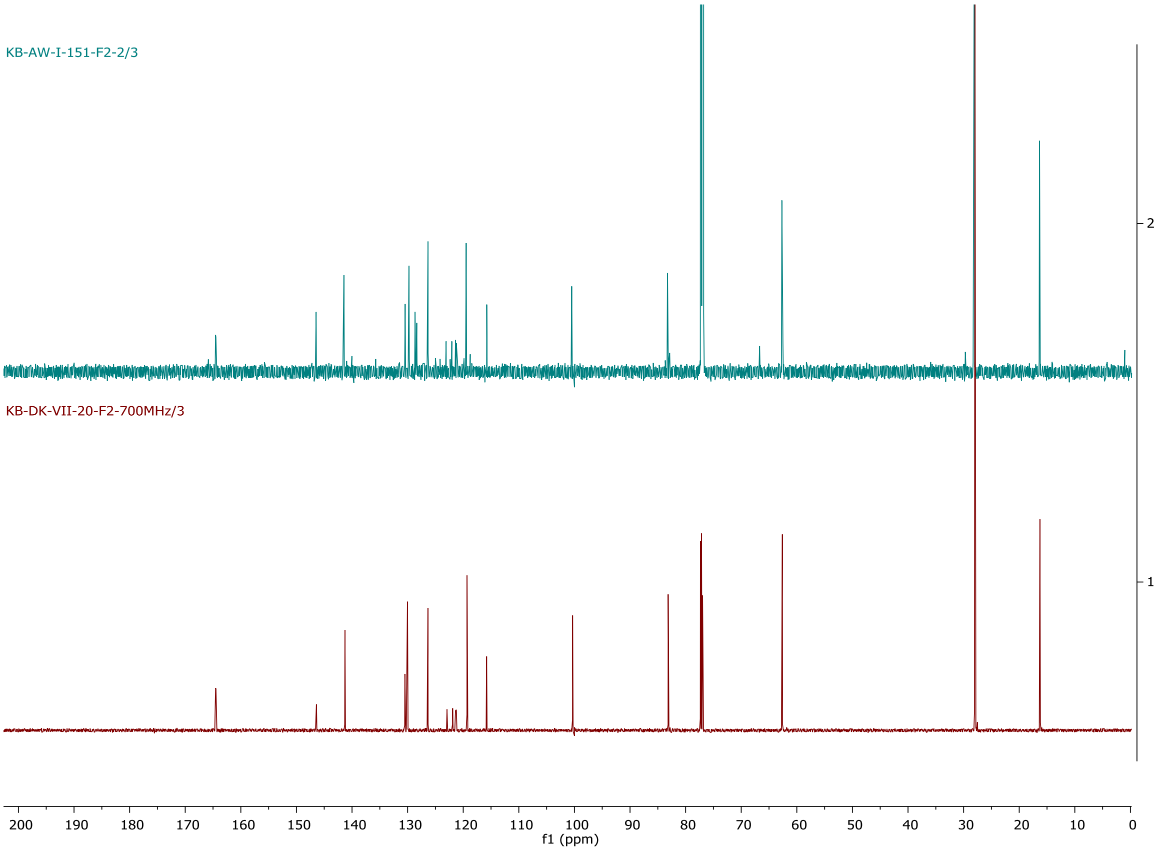

*Figure S64. Comparing the ^{13}C NMR spectra of compounds **5** (blue) and **5’** (red)*

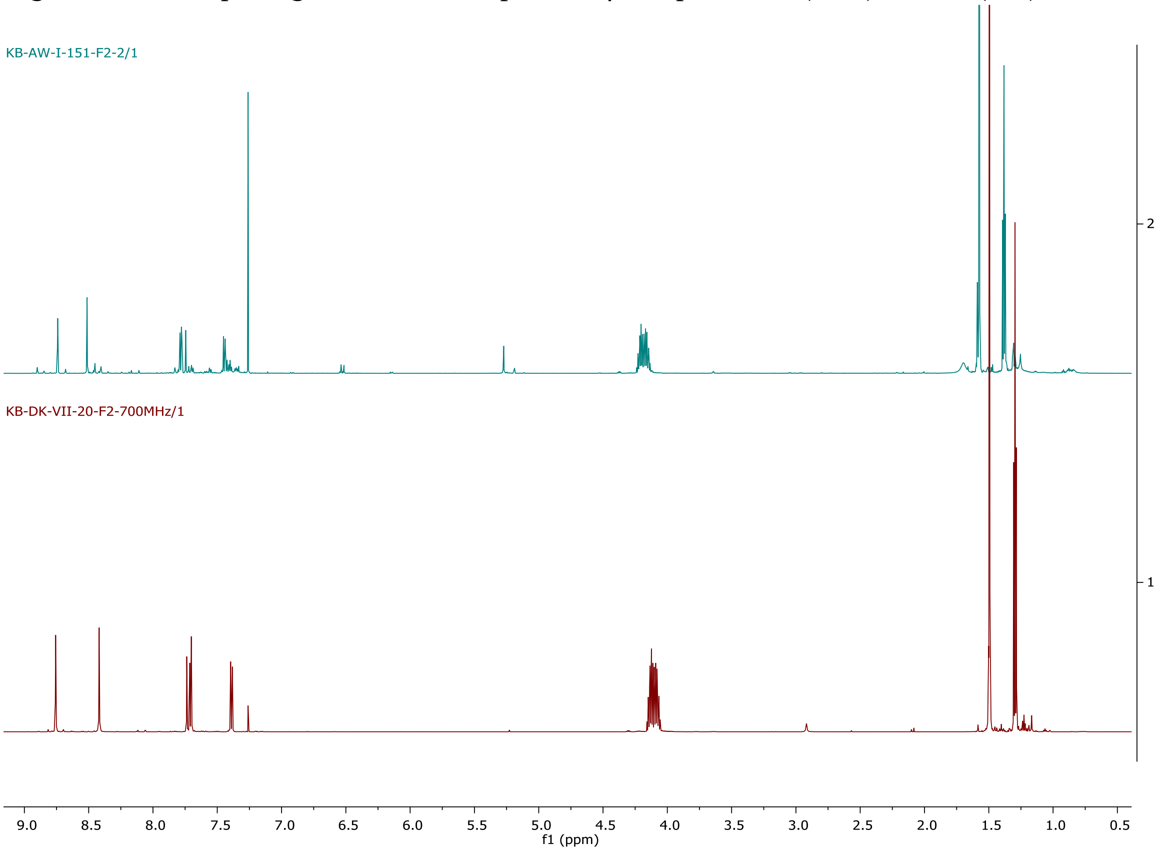

*Figure S65. Comparing the ^{1}H NMR spectra of compounds **5** (blue) and **5’** (red)*

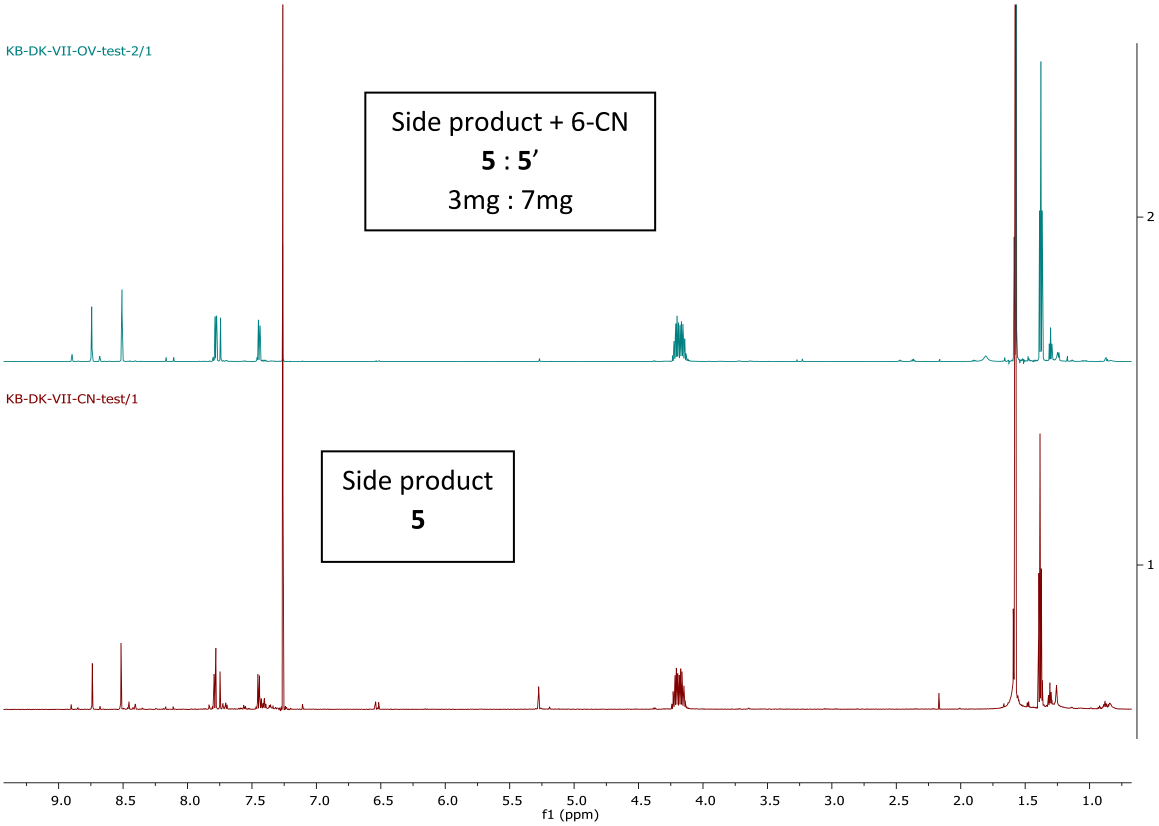

Figure S66. ^{1}H NMR spectra of mixture of side product **5** and synthesized compound **5'**

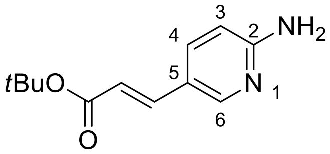

(*E*)-*tert*-Butyl 3-(6-aminopyridin-3-yl)acrylate

Yield (86%) **^{1}H NMR** (250 MHz, $CDCl_3$) δ: 1.54 (s, C(C<u>H</u>$_3$)$_3$, 9H), 6.20 (d, $^{3}J_{HH}$ = 16.0, CO-C<u>H</u>=CH 1H), 6.51 (d, $^{3}J_{HH}$ = 8.7, $CH_{(3)}$, 1H), 7.49 (d, $^{3}J_{HH}$ = 16.0, CO-CH=C<u>H</u>, 1H), 7.64 (dd, $^{3}J_{HH}$ = 8.7, $^{4}J_{HH}$ = 2.2, $CH_{(4)}$, 1H), 8.19 (d, $^{4}J_{HH}$ = 2.2, $CH_{(6)}$, 1H).

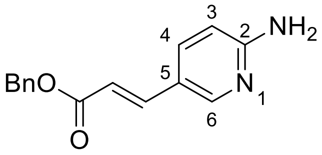

(*E*)-Benzyl 3-(6-aminopyridin-3-yl)acrylate

Yield (92%), **^{1}H NMR** (250 MHz, $CDCl_3$) δ: 5.25 (s, C<u>H</u>$_2$Ph, 2H), 6.32 (d, $^{3}J_{HH}$ = 15.9, CO-C<u>H</u>=CH 1H), 6.51 (d, $^{3}J_{HH}$ = 8.8, $CH_{(3)}$, 1H), 7.34 – 7.44 (m, Ph, 5H), 7.63 (d, $^{3}J_{HH}$ = 15.8, CO-CH=C<u>H</u>, 1H), 7.65 (dd, $^{3}J_{HH}$ = 8.8, $^{4}J_{HH}$ = 2.1, $CH_{(4)}$, 1H), 8.20 (d, $^{4}J_{HH}$ = 2.1, $CH_{(6)}$, 1H). **^{13}C NMR** (250 MHz, $CDCl_3$) δ: 66.23 (s, Ph-<u>C</u>H$_2$, 1C), 108.69 (s, $C_{(3)}$, 1C), 114.47 (s, CO-<u>C</u>H=CH, 1C), 120.78 (s, $C_{(5)}$, 1C), 120.23 (s, $C_{(Ar)}$ 2C), 120.26 (s, $C_{(Ar)}$, 1C), 128.60 (s, $C_{(Ar)}$,

2C), 135.79 (s, $C_{(3)}$, 1C), 136.20 (s, $C_{(Ar)}$-CH_2, 1C), 142.25 (s, CO-CH=CH, 1C), 159.68 (s, $C_{(3)}$, 1C), 167.01 (s, CO_2Bn, 1C).

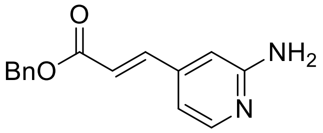

(*E*)-Benzyl 3-(2-aminopyridin-4-yl)acrylate

^{1}H NMR (250 MHz, $CDCl_3$) δ: 5.25 (s, $COCH_2Bn$, 2H), 6.54 (bs, CH(3), 1H), 6.55 (d, $^3J_{HH}$ = 16.0 Hz, CH=CH, 1H), 6.75 (dd, , $^3J_{HH}$ = 5.5, $^4J_{HH}$ = 1.3 Hz, CH(5), 1H), 7.46 – 7.33 (m, $C_6H_5CH_2CO$, 5H), 7.53 (d, $^3J_{HH}$ = 16.0 Hz, CH=CH, 1H), 8.06 (d, $^3J_{HH}$ = 5.5 Hz, CH(6), 1H).

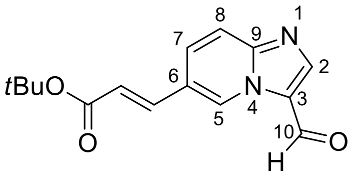

(*E*)-*tert*-Butyl 3-(3-formylimidazo[1,2-*a*]pyridin-6-yl)acrylate

Yield (86%) **^{1}H NMR** (250 MHz, $CDCl_3$) δ: 1.53 (s, CCH_3, 9H), 6.43 (d, $^3J_{HH}$ = 16.0, tBuO$_2$C-CH=CH, 1H), 7.58 (d, $^3J_{HH}$ = 16.0, tBuO$_2$C-CH=CH, 1H), 7.72 (d, $^3J_{HH}$ = 9.3, $CH_{(7)}$, 1H), 7.78 (d, $^3J_{HH}$ = 9.3, $CH_{(8)}$, 1H), 8.31 (s, $CH_{(2)}$, 1H), 9.59 (bs, $CH_{(5)}$, 1H), 9.95 (s, $C(O)H_{(10)}$, 1H).

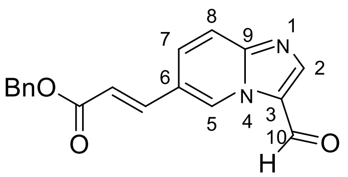

(*E*)-Benzyl 3-(3-formylimidazo[1,2-*a*]pyridin-6-yl)acrylate

Yield (88%) **^{1}H NMR** (250 MHz, $CDCl_3$) δ: 5.23 (s, CH_2-Ph, 2H), 6.52 (d, $^3J_{HH}$ = 16.0, BnO_2C-CH=CH, 1H), 7.34-7.49 (m, $PhCH_2$, 5H), 7.67 (d, $^3J_{HH}$ = 9.3, $CH_{(7)}$, 1H), 7.70 (d, $^3J_{HH}$ = 16.0, BnO_2C-CH=CH, 1H), 7.73 (d, $^3J_{HH}$ = 9.3, $CH_{(8)}$, 1H), 8.26 (s, $CH_{(2)}$, 1H), 8.56 (bs, $CH_{(5)}$, 1H), 9.90 (s, $C(O)H_{(10)}$, 1H). **^{13}C NMR** (700 MHz, $CDCl_3$) δ: 66,89 (s, Ph-CH_2, 1C), 118.27 (s, $C_{(8)}$, 1C), 120.40 (s, BnO_2C-CH=CH, 1C), 123.79 (s, $C_{(6)}$, 1C), 125.52 (s, $C_{(3)}$, 1C), 127.74 (s, $C_{(7)}$, 1C), 128.52 (s, $C_{(Ar)}$, 2C), 128.57 (s, $C_{(Ar)}$, 1C), 128.83 (s, $C_{(Ar)}$, 2C), 129.67 (s, $C_{(5)}$, 1C), 135 (s, $C_{(Ar)}$-CH_2, 1C), 140.14 (BnO_2C-CH=CH, 1C), 147.38 (s, $C_{(2)}$, 1C), 149.13 (s, $C_{(9)}$, 1C), 165,93 (s, CO_2Bn, 1C), 178.37 (s, CHO, 1C).

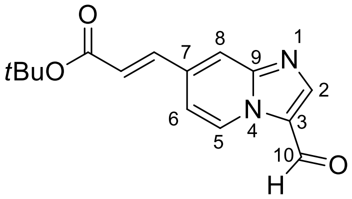

(*E*)-*tert*-Butyl 3-(3-formylimidazo[1,2-*a*]pyridin-7-yl)acrylate

Yield (30%), **^{1}H NMR** (250 MHz, $CDCl_3$) δ: 1.54 (s, CC<u>H</u>$_3$, 9H), 6.53 (d, $^{3}J_{HH}$ = 16.0, *t*BuO_2C-C<u>H</u>=CH, 1H), 7.06 (dd, $^{3}J_{HH}$ = 7.3, $^{4}J_{HH}$ = 1.4, CH$_{(6)}$, 1H), 7.59 (d, $^{3}J_{HH}$ = 16.0, *t*BuO_2C-CH=C<u>H</u>, 1H), 7.83 (bs, CH$_{(8)}$, 1H), 8.34 (s, CH$_{(2)}$, 1H), 9.42 (d, $^{3}J_{HH}$ = 7.3, CH$_{(5)}$, 1H), 9.95 (s, C(O)H$_{(10)}$, 1H).

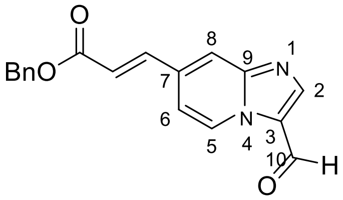

(*E*)-Benzyl 3-(3-formylimidazo[1,2-*a*]pyridin-7-yl)acrylate

Yield (70%), **^{1}H NMR** (250 MHz, $CDCl_3$) δ: 5.29 (s, C<u>H</u>$_2$-Ph, 2H), 6.64 (d, $^{3}J_{HH}$ = 16.0, BnO_2C-C<u>H</u>=CH, 1H), 7.06 (dd, $^{3}J_{HH}$ = 7.3, $^{4}J_{HH}$ = 1.5, CH$_{(6)}$, 1H), 7.35-7.46 (m, <u>Ph</u>CH_2, 5H), 7.74 (d, $^{3}J_{HH}$ = 16.0, BnO_2C-CH=C<u>H</u>, 1H), 7.86 (bs, CH$_{(8)}$, 1H), 8.35 (s, CH$_{(2)}$, 1H), 9.44 (d, $^{3}J_{HH}$ = 7.3, CH$_{(5)}$, 1H), 9.97 (s, C(O)H$_{(10)}$, 1H).

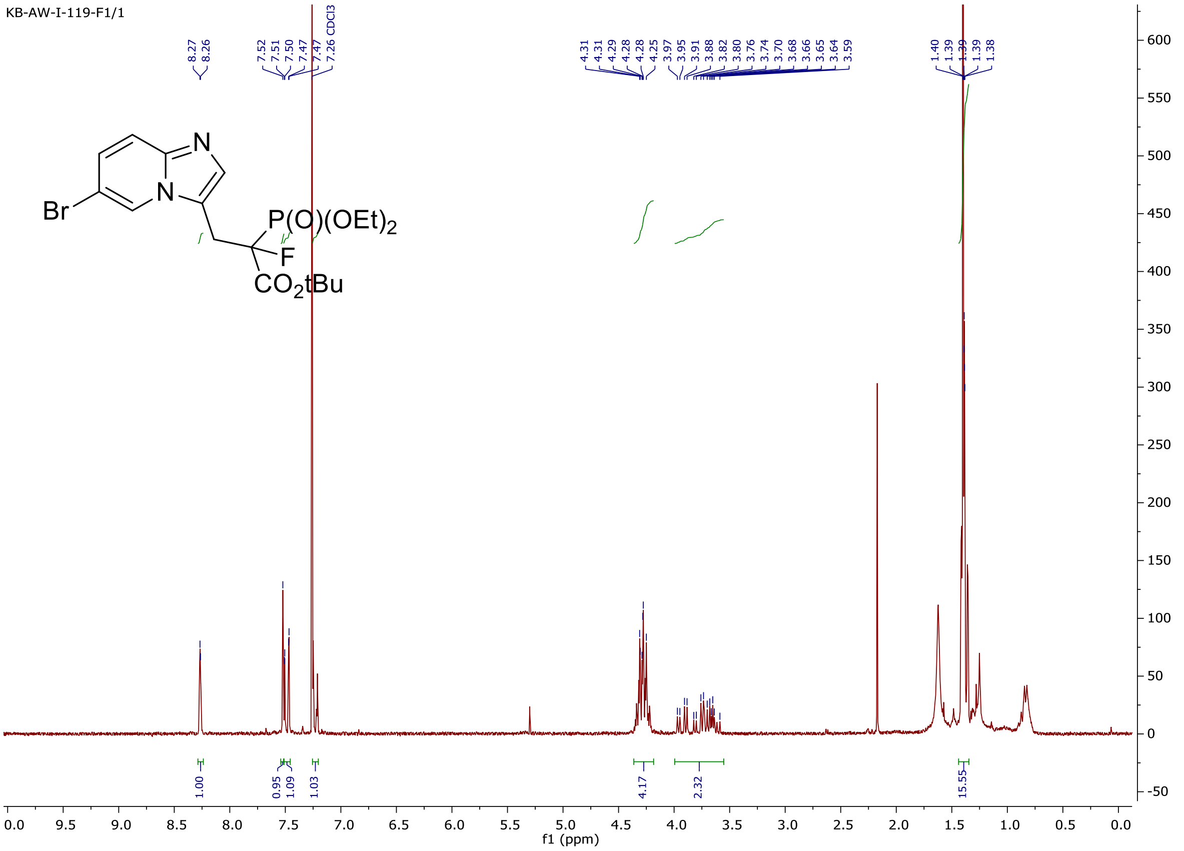

Figure S67. ^{1}H NMR of compound ***23*** *(700 MHz, $CDCl_3$)*

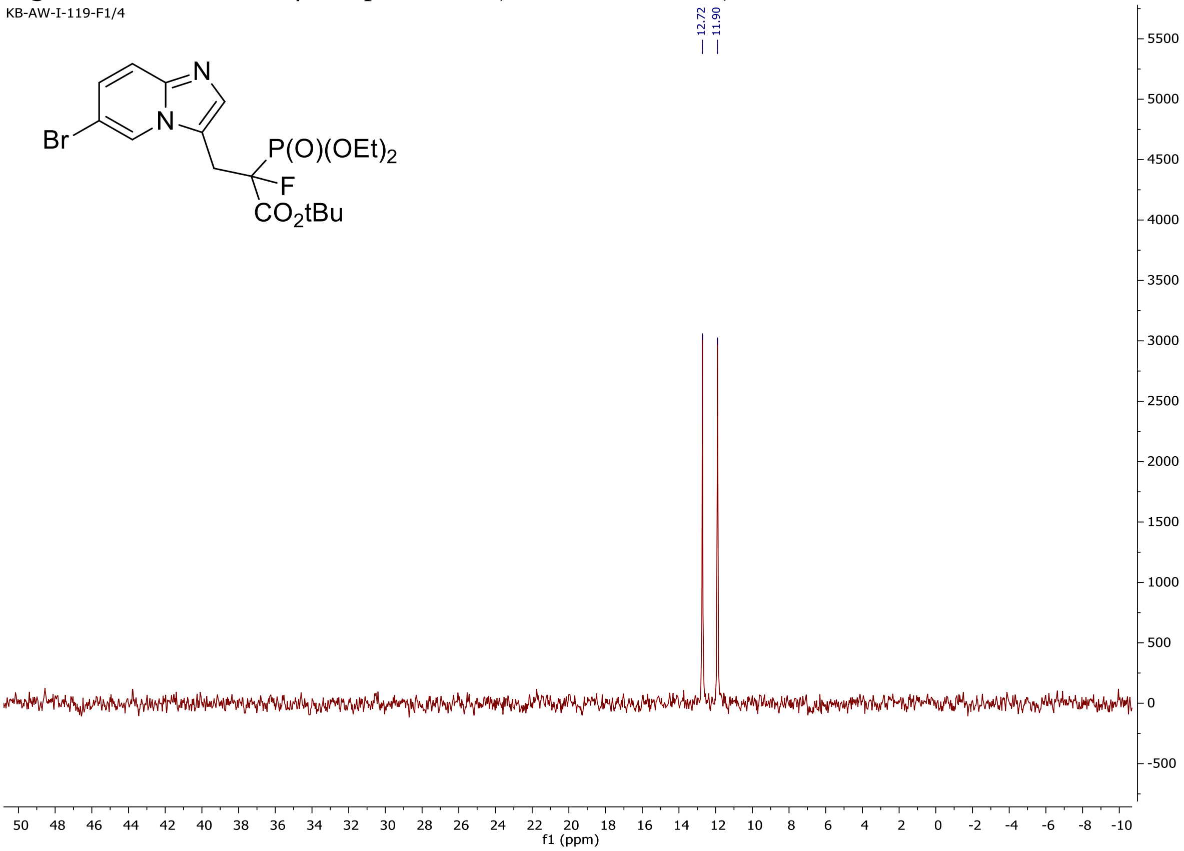

Figure S68. ^{31}P NMR of compound ***23*** *(283 MHz, $CDCl_3$*

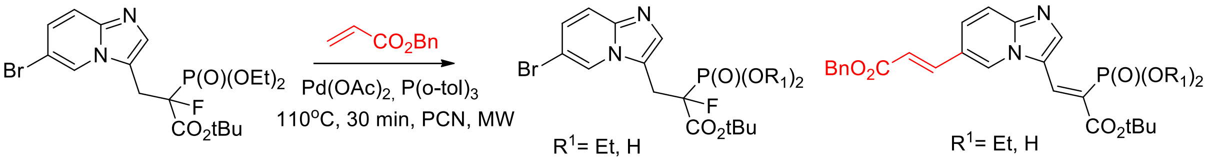

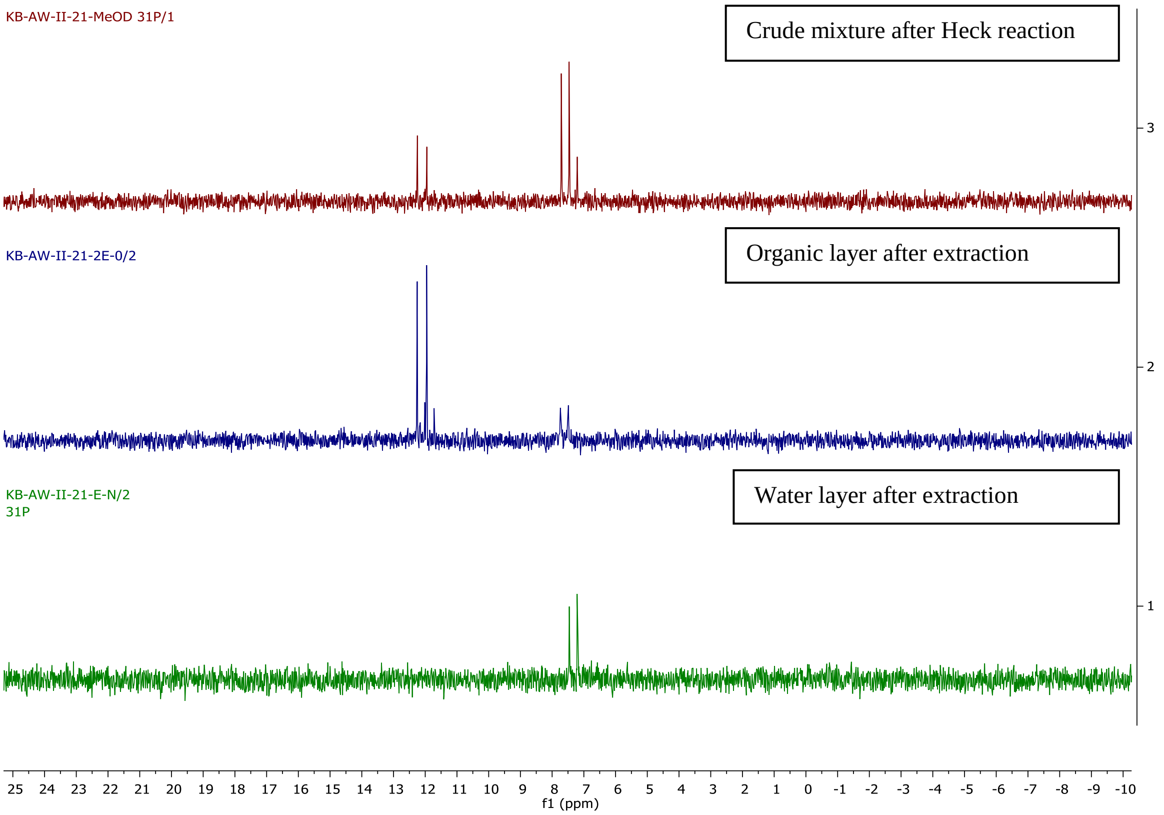

Figure 69. Comparing ^{31}P NMR spectra of reaction mixture after Heck reaction performed on compound ***23.***

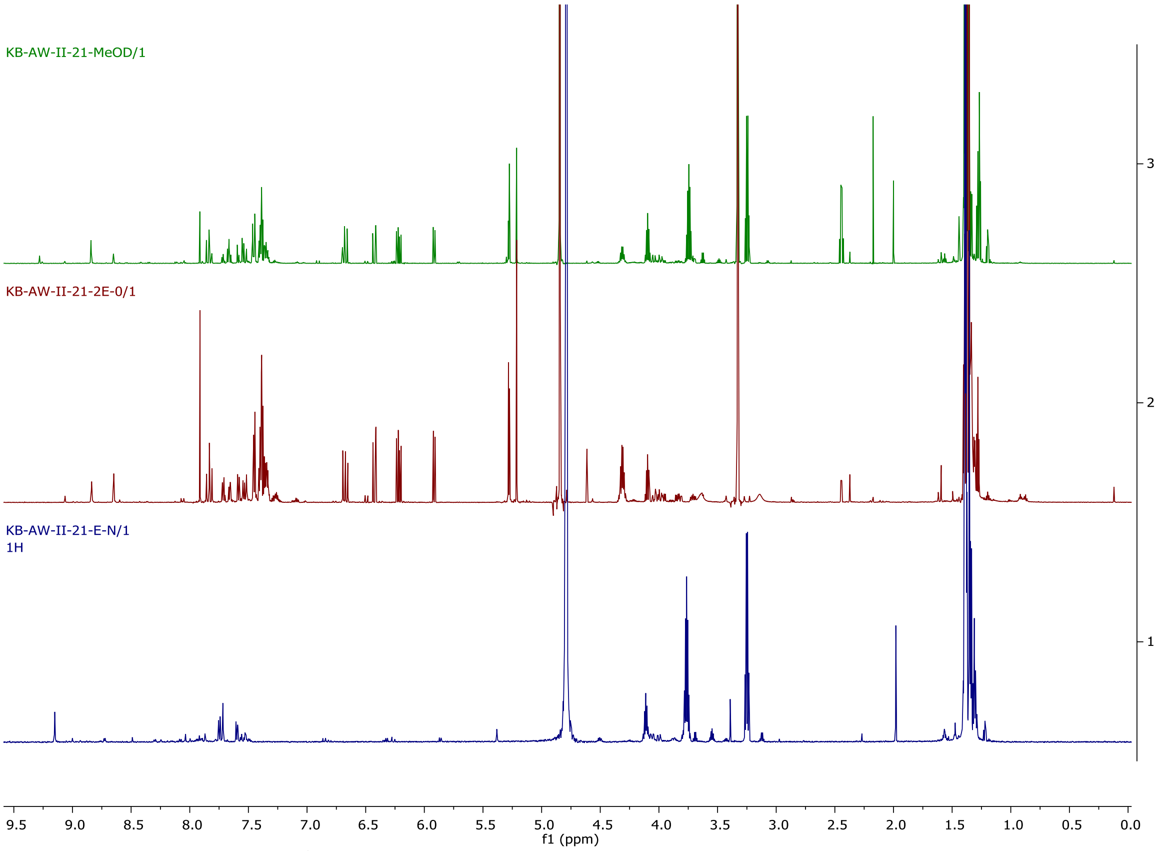

Figure 70. Comparing ^1H NMR spectra of reaction mixture after Heck reaction performed on compound ***23***

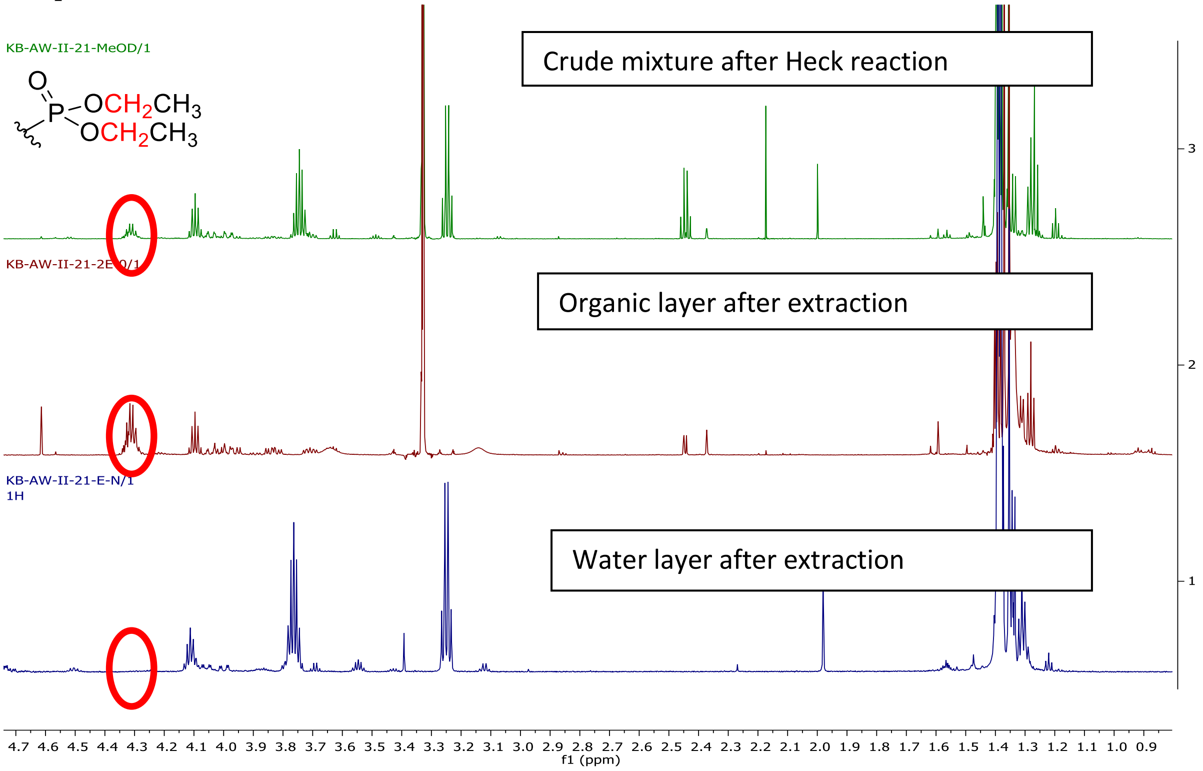

Figure 71. Comparing ^1H NMR spectra of reaction mixture after Heck reaction performed on compound ***23*** *(range 0-4.7)*

General procedure for the synthesis of starting materials (**1, 2, and 23**) [1]

*General procedure for the synthesis of compounds (**1**).*

The reaction was carried out under argon atmosphere using oven-dried glassware. A 100 mL two-necked round-bottomed flask was charged with triethyl phosphonoacetate (1.79 g, 8.0 mmol, 1.2 equiv) dissolved in DCM (20 mL). The solution was cooled below −30 °C (internal temperature control), and $TiCl_4$ (0.877 mL, 8 mmol, 1.2 equiv) was slowly added via a syringe, followed by Et_3N (2.63 mL, 0.0187 mol, 2.8 equiv). The reaction mixture was stirred for 15 min at −30 °C. Then, a solution of 6-bromoimidazo[1,2-*a*]pyridine-3-carbaldehyde (6.67 mmol, 1 equiv) in DCM (20 mL) was dropwisely added over 15 min at a the temperature kept below −30 °C. The resulting mixture was stirred at room temperature for 16 h, then quenched with 25 mL of water and was made basic with saturated Na_2CO_3 aqueous solution to pH > 9. The aqueous layer was extracted with diethyl ether (5 × 25 mL). The organic layer was dried over anhydrous Mg_2SO_4, the solvent evaporated and the residue subjected to column chromatography using DCM/acetone 85:15 as eluent to give the product as (*Z/E*) mixture in the form of an orange oil.

***tert*-Butyl 3-(6-bromoimidazo[1,2-*a*]pyridin-3-yl)-2-(diethoxyphosphoryl)acrylate** (**1a**). **^{1}H NMR** (700 MHz, $CDCl_3$) δ 1.31 (t, $^3J_{HH}$ = 7.5, C<u>H</u>$_3$CH$_2$OP, 6H), 1.51 (s, C(C<u>H</u>$_3$)$_3$, 9H), 4.06 – 4.17 (m, C<u>H</u>$_2$OP , 4H), 7.35 (dd, $^3J_{HH}$ = 9.4, $^4J_{HH}$ = 1.5, C<u>H</u>$_{Ar(7)}$, 1H), 7.52 (d, $^3J_{HH}$ = 9.4, C<u>H</u>$_{Ar(8)}$, 1H), 7.71 (d, $^3J_{PH}$ = 23.9, C=C<u>H</u>, 1H), 8.36 (s, C<u>H</u>$_{Ar(2)}$, 1H), 8.42 (bs, C<u>H</u>$_{Ar(5)}$, 1H). **^{13}C NMR** (176 MHz, $CDCl_3$) δ 16.32 (d, $^3J_{PC}$ = 6.5, <u>C</u>H$_3$CH$_2$OP, 2C), 28.04 (s, C(<u>C</u>H$_3$)$_3$, 3C), 62.50 (d, $^2J_{PC}$ = 5.1, <u>C</u>H$_2$OP, 2C), 82.83 (s, <u>C</u>(CH$_3$)$_3$, 1C), 109.03 (s, <u>C</u>$_{Ar(6)}$, 1C), 118.96 (s, <u>C</u>H$_{Ar(8)}$, 1C), 119.78 (d, $^1J_{PC}$ = 181.4, P<u>C</u>, 1C), 120.58 (d, $^3J_{PC}$ = 25.2, <u>C</u>$_{Ar(3)}$, 1C), 124.09 (s, <u>C</u>H$_{Ar(5)}$, 1C), 130.25 (s, <u>C</u>H$_{Ar(7)}$, 1C), 131.54 (d, $^2J_{PC}$ = 10.1, PC=<u>C</u>H, 1C), 140.59 (s, <u>C</u>H$_{Ar(2)}$, 1C), 145.93 (s, <u>C</u>$_{Ar(9)}$, 1C), 164.85 (d, $^2J_{PC}$ = 10.3, <u>C</u>O$_2$, 1C). **^{31}P NMR** (238 MHz, $CDCl_3$) δ 15.93.

Ethyl 3-(6-bromoimidazo[1,2-*a*]pyridin-3-yl)-2-(diethoxyphosphoryl)acrylate (**1b**). Yield: 52%, scale: 0.34 g, (*E*)/(*Z*) 50/1, $R_{f(E)}$ 0.36, (CH_2Cl_2:acetone 85:15), **(*E*)-1b:** **^{31}P NMR** (101 MHz, $CDCl_3$) δ: 16.52 (*E*) and 13.27 (*Z*), **^{1}H NMR** (700 MHz, $CDCl_3$) δ: 1.35 (t, $^3J_{HH}$ = 7.10, C<u>H</u>$_3$CH$_2$OC, 3H), 1.36 (t, $^3J_{HH}$ = 7.1, C<u>H</u>$_3$CH$_2$OP, 6H), 4.12 - 4.25 (m, C<u>H</u>$_2$OP, 4H), 4.36 (q, $^3J_{HH}$ = 7.1, C<u>H</u>$_2$OC, 2H), 7.43 (dd, $^3J_{HH}$ = 9.4, $^4J_{HH}$ = 1.7, CH$_{Ar(7)}$, 1H), 7.59 (d, $^3J_{HH}$ = 9.4, CH$_{Ar(8)}$, 1H), 7.90 (d, $^3J_{PH}$ (*Z*) = 23.8, C=C<u>H</u>, 1H), 8.45 (s, CH$_{Ar(2)}$, 1H), 8.52 (d, $^4J_{HH}$ = 1.7, CH$_{Ar(5)}$, 1H), **^{13}C NMR** (176 MHz, $CDCl_3$) δ: 14.2 (s, <u>C</u>H$_3$CH$_2$OC), 16.42 (s, <u>C</u>H$_3$CH$_2$OP, 1C), 16.44 (s, <u>C</u>H$_3$CH$_2$OP, 1C), 61.8 (s, <u>C</u>H$_2$OC, 1C), 62.87 (s, <u>C</u>H$_2$OP, 1C), 62.90 (s, <u>C</u>H$_2$OP, 1C), 109.4 (s, CH$_{Ar(6)}$, 1C), 117.5 (d, $^1J_{PC}$ = 184.3, <u>C</u>=CH, 1C), 119.1 (s, CH$_{Ar(8)}$, 1H), 120.8 (d, $^3J_{PC}$ = 24.2, C$_{Ar(3)}$, 1C), 124.3 (s, CH$_{Ar(5)}$, 1C), 130.8 (s, CH$_{Ar(7)}$, 1C), 133.3 (d,

$^{2}J_{PC}$ = 10.1, C=C̲H, 1C), 141.5 (s, $CH_{Ar(2)}$, 1C), 146.3 (s, $C_{Ar(9)}$, 1C), 165.7 (d, $^{2}J_{PC}$ = 11.7, C̲O_2, 1C).

***tert*-Butyl 3-(7-bromoimidazo[1,2-*a*]pyridin-3-yl)-2-(diethoxyphosphoryl)acrylate (1c),** ^{1}**H NMR** (700 Hz, $CDCl_3$) δ: 1.35 (t, $^{3}J_{HH}$ = 7.0 Hz, $C\underline{H}_3CH_2OP$, 6H), 1.56 (s, $C(C\underline{H}_3)_3$, 9H), 4.04 – 4.25 (m, $CH_3C\underline{H}_2OP$, 4H), 7.09 (dd, $^{3}J_{HH}$ = 7.2 Hz, $^{4}J_{HH}$ = 1.9 Hz, $CH_{(6)}$, 1H), 7.77 (d, $^{3}J_{HH}$ = 23,9 Hz, *(E)* CH̲=CP, 1H), 7.87 (dd, $^{4}J_{HH}$ = 1.9 Hz, $^{5}J_{HH}$ = 0.8 Hz, $C\underline{H}_{(8)}$, 1H), 8.21 (d, $^{3}J_{HH}$ = 7.1 Hz, $C\underline{H}_{(5)}$, 1H), 8.42 (s, $C\underline{H}_{(2)}$, 1H), ^{31}**P NMR** (700 Hz, $CDCl_3$) δ: 16.60 – *(E)* CH=CP.

***tert*-Butyl 3-(8-bromoimidazo[1,2-*a*]pyridin-3-yl)-2-(diethoxyphosphoryl)acrylate (1d),** ^{1}**H NMR** (700 Hz, $CDCl_3$) δ: 1.37 (t, $^{3}J_{HH}$ = 7.1 Hz, $C\underline{H}_3CH_2OP$, 6H), 1.57 (s, $C(C\underline{H}_3)_3$, 9H), 4.06 – 4.28 (m, $CH_3C\underline{H}_2OP$, 4H), 6.89 (t, $^{3}J_{HH}$ = 7.2 Hz, $CH_{Ar(6)}$, 1H), 7.62 (dd, $^{3}J_{HH}$ = 7.2 Hz, $^{4}J_{HH}$ = 1,0 Hz, $C\underline{H}_{(7)}$, 1H), 7.74 (d, $^{3}J_{HH}$ = 23,8 Hz, *(E)* CH̲=CP, 1H), 8.32 (m, $C\underline{H}_{(5)}$, 1H), 8.42 (s, $C\underline{H}_{(2)}$, 1H), ^{31}**P NMR** (700 Hz, $CDCl_3$) δ: 15.90 – *(E)* CH=CP.

***tert*-Butyl 3-(6-chloroimidazo[1,2-*a*]pyridin-3-yl)-2-(diethoxyphosphoryl)acrylate (1e),** ^{1}**H NMR** (700 Hz, $CDCl_3$) δ: 1.37 (t, $^{3}J_{HH}$ = 7.1 Hz, $C\underline{H}_3CH_2OP$, 6H), 1.57 (s, $C(C\underline{H}_3)3$, 9H), 4.06 – 4.28 (m, $CH_3C\underline{H}_2OP$, 4H), 7.32 (dd, $^{3}J_{HH}$ = 9.5 Hz, $^{4}J_{HH}$ = 1.8 Hz, $CH_{(7)}$, 1H), 7.64 (dd, $^{3}J_{HH}$ = 9.5 Hz, $^{4}J_{HH}$ = 0.8 Hz, $CH_{(8)}$, 1H), 7.76 (d, $^{3}J_{HH}$ = 23,9 Hz, *(E)* CH̲=CP, 1H), 8.37 (d, $^{4}J_{HH}$ = 1.8 Hz, $C\underline{H}_{(5)}$, 1H), 8.52 (s, $C\underline{H}_{(2)}$, 1H), ^{31}**P NMR** (700 Hz, $CDCl_3$) δ: 16.34 – *(E)* CH=CP.

*General procedure for the synthesis of compounds (**2**).*

Ethyl 3-(6-bromoimidazo[1,2-*a*]pyridin-3-yl)-2-(diethoxyphosphoryl)propanoate (**2b**). A 50 mL two-necked round-bottomed flask was charged with **1b** (1.83 mmol, 1 equiv), $NiCl_2{\cdot}6H_2O$ (0.52 g, 2.2 mmol, 1.2 equiv) which were dissolved in methanol (10 mL). The flask was submerged in a dry ice/acetone cooling bath (−40 °C) and then $NaBH_4$ (0.083 g, 2.2 mmol, 1.2 equiv) was added. The mixture was stirred at (−40 °C, internal control) for 8 min then quenched by the addition of 4 mL NH_4Cl. The solution was made basic with saturated Na_2CO_3 solution to pH > 9. The aqueous layer was extracted with dichloromethane (4 × 10 mL). The organic layer was dried over anhydrous $MgSO_4$, the solvent evaporated and the residue subjected to column chromatography using DCM/acetone 100:20 as eluent to give the product as orange oil.

***tert*-Butyl 3-(6-bromoimidazo[1,2-*a*]pyridin-3-yl)-2-(diethoxyphosphoryl)propanoate** (**2a**). **^{1}H NMR** (700 MHz, $CDCl_3$) δ 8.18 (dd, $^{4}J_{HH}$ = 1.8, $^{5}J_{HH}$ = 0.8, $C\underline{H}_{Ar(5)}$, 1H), 7.49 (dd, $^{3}J_{HH}$ = 9.5, $^{5}J_{HH}$ = 0.8, $C\underline{H}_{Ar(8)}$, 1H), 7.45 (s, $C\underline{H}_{Ar(2)}$, 1H), 7.22 (dd, $^{3}J_{HH}$ = 9.5, $^{4}J_{HH}$ = 1.8, $C\underline{H}_{Ar(7)}$, 1H), 4.18-4.25 (m, $C\underline{H}_2OP$, 4H), 3.53 (ddd, $^{2}J_{HH}$ =15.8, $^{3}J_{HH}$ =11.5, $^{3}J_{PH}$ = 6.8 $C\underline{H}_2CHP$, 1H), 3.30 (ddd, $^{2}J_{HH}$ = 15.8, $^{3}J_{PH}$ = 10.0, $^{3}J_{HH}$ = 3.1 $C\underline{H}_2CHP$, 1H), 3.23 (ddd, $^{2}J_{PH}$ = 22.8, $^{3}J_{HH}$ = 11.5, $^{3}J_{HH}$ = 3.1 $CH_2C\underline{H}P$, 1H), 1.39 (s, $C(C\underline{H}_3)_3$, 9H), 1.35-1.38 (m, $C\underline{H}_3CH_2OP$, 6H). **^{13}C NMR** (176 MHz, $CDCl_3$) δ 167.34 (d, $^{2}J_{PC}$ = 5.1, $\underline{C}O_2$, 1C), 144.12 (s, $\underline{C}_{Ar(9)}$, 1C), 132.60 (d, $^{4}J_{PC}$ = 9.3, $\underline{C}H_{Ar(2)}$, 1C), 127.32 (s, $\underline{C}H_{Ar(7)}$, 1C), 123.57 (s, $\underline{C}H_{Ar(5)}$, 1C), 122.12 (d, $^{3}J_{PC}$ = 19.1, $\underline{C}_{Ar(3)}$, 1C), 118.72 (s, $\underline{C}H_{Ar(8)}$, 1C), 107.36 (s, $\underline{C}_{Ar(6)}$, 1C), 82.90 (s, $\underline{C}(CH_3)_3$, 1C), 63.24 (d, $^{2}J_{PC}$ = 6.8, $\underline{C}H_2OP$, 1C), 63.10 (d, $^{2}J_{PC}$ = 6.8, $\underline{C}H_2OP$, 1C), 45.35 (d, $^{1}J_{PC}$ = 128.5, $\underline{C}HP$, 1C), 27.94 (d, $^{5}J_{PC}$ = 3.5, $C(\underline{C}H_3)_3$, 3C), 16.53 (d, $^{3}J_{PC}$ = 3.1, $\underline{C}H_3CH_2OP$, 1C), 21.70 (s, $\underline{C}H_2CHP$, 1C), 16.56 (d, $^{2}J_{PC}$ = 3.1, $\underline{C}H_3CH_2OP$, 1C). **^{31}P NMR** (283 MHz, $CDCl_3$) δ 21.32.

Ethyl 3-(6-bromoimidazo[1,2-*a*]pyridin-3-yl)-2-(diethoxyphosphoryl)propanoate (**2b**). Yield: 75 %, scale (0.32 g), R_f 0.08, (CH_2Cl_2:acetone 85:15), **^{1}H NMR** (700 MHz, $CDCl_3$) δ: 1.18 (t, $^{3}J_{HH}$ = 7.10, $C\underline{H_3}CH_2OC$, 3H), 1.34 (t, $^{3}J_{HH}$ = 7.10, $C\underline{H_3}CH_2OP$, 6H), 3.21 - 3.41 (m, $C\underline{H}_2C\underline{H}P$, 2H), 3.45-3.64 (m, $C\underline{H}_2CHP$, 1H), 4.03-4.28 (m, $C\underline{H_2}OP$, $C\underline{H_2}OC$, 6H), 7.20 (dd, $^{3}J_{HH}$ = 9.5, $^{4}J_{HH}$ = 1.8, $CH_{Ar(7)}$, 1H), 7.40 (s, $CH_{Ar(2)}$, 1H), 7.46 (d, $^{3}J_{HH}$ = 9.5, $^{5}J_{HH}$ = 0.8, $CH_{Ar(8)}$, 1H), 8.16 (d, $^{4}J_{HH}$ = 1.8, $^{5}J_{HH}$ = 0.8, $CH_{Ar(5)}$,1H), **^{31}P NMR** (283 MHz, $CDCl_3$): 21.40**.**

*General procedure for the synthesis of compounds (**23**).*

Ethyl 3-(6-bromoimidazo[1,2-*a*]pyridin-3-yl)-2-(diethoxyphosphoryl)-2-fluoropropan-oate (**23**). The reaction was carried out under an argon atmosphere using oven-dried glassware. A solution of phosphonocarboxylate triester **2b** (1 equiv, 1.01 mmol) in anhydrous THF (8 mL) was cooled to −78 °C, and *n*-BuLi (1.6 M in hexanes, 1.5 equiv) was added via a syringe. The solution was stirred for 10 min at −78 °C, allowed to warm to 0 °C, and stirred at this temperature for 1 h. Then it was cooled back to −78 °C and a solution of *N*-fluoro-*N*-(phenylsulfonyl)benzenesulfonamide (1.2 equiv) in anhydrous THF (8 mL) was added dropwise via syringe. The reaction mixture was stirred at − 78 ° C for 10 min, warmed to rt and stirred for additional 4.5 h. The reaction mixture was quenched with saturated aqueous NH_4Cl, and concentrated under vacuum. It was made basic with Na_2CO_3 solution (to pH 9)

and extracted with $CHCl_3$ (3 ×). The organic layer was dried over anhydrous $MgSO_4$, concentrated to dryness under vacuum, and purified by column chromatography.

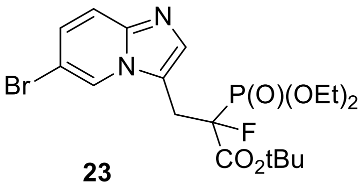

23

***tert*-Butyl 3-(6-bromoimidazo[1,2-*a*]pyridin-3-yl)-2-(diethoxyphosphoryl)-2-fluoro-propanoate** (**23**). Yield: 86%, scale: 0.37 g, (CH_2Cl_2/acetone 75:25), **^{1}H NMR** (250 MHz, $CDCl_3$) δ: 1.30 - 1.48 (m, C(C$\underline{H}_3$)$_3$, C$\underline{H}_3$CH$_2$O(O)P, 15H), 3.48 - 4.07 (m, C$\underline{H}_2$CFP, 2H), 4.11 - 4.40 (m, CH$_3$C$\underline{H}_2$O(O)P, 4H), 7.24 (dd, $^3J_{HH}$ = 9.5, $^4J_{HH}$ = 1.8 $CH_{Ar(7)}$, 1H), 7.49 (dd, $^3J_{HH}$ = 9.5, $^5J_{HH}$ = 0.8 $CH_{Ar(8)}$, 1H), 7.52 (s, $CH_{Ar2,}$, 1H), 8.27 (bs, $CH_{Ar(5)}$, 1H), **^{31}P NMR** (101 MHz, $CDCl_3$) δ: 12.31 (d, $^2J_{PF}$ = 83.3)**.**

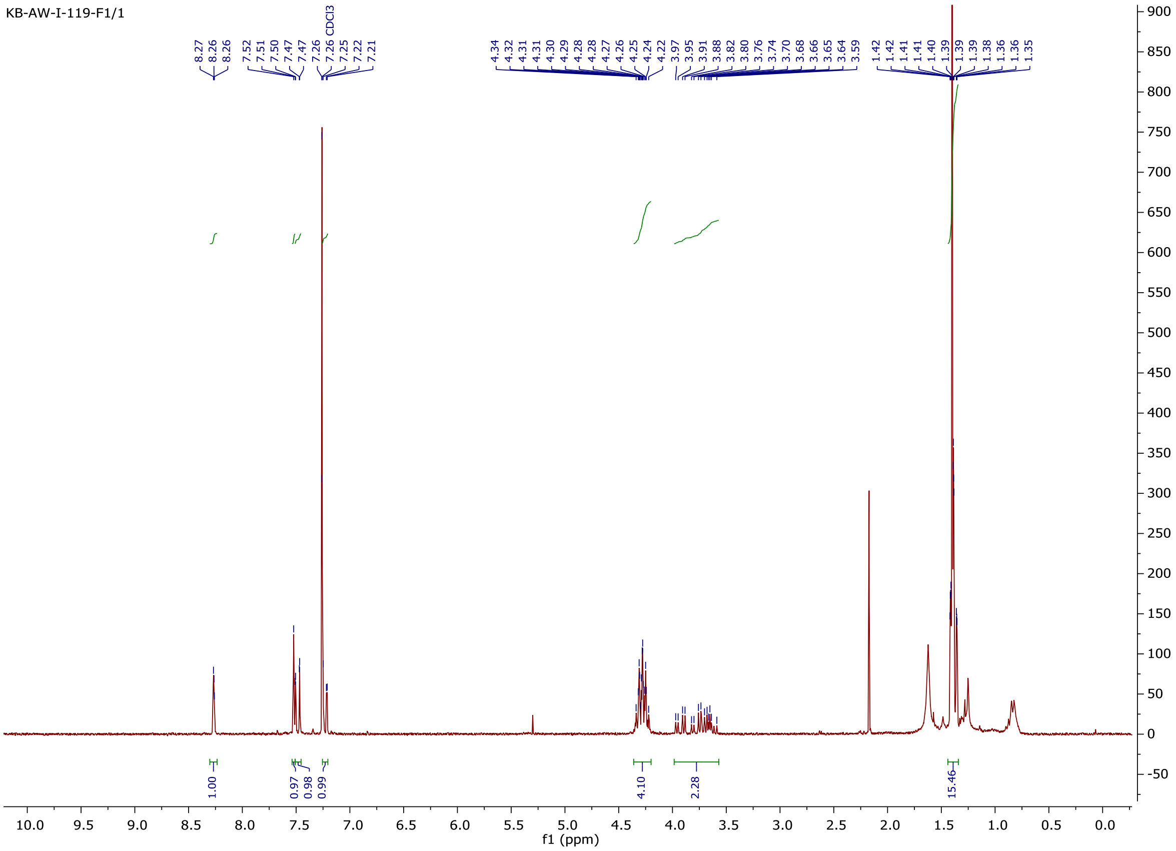

*Figure S72. ^{1}H NMR of compound **23** (250 MHz, $CDCl_3$)*

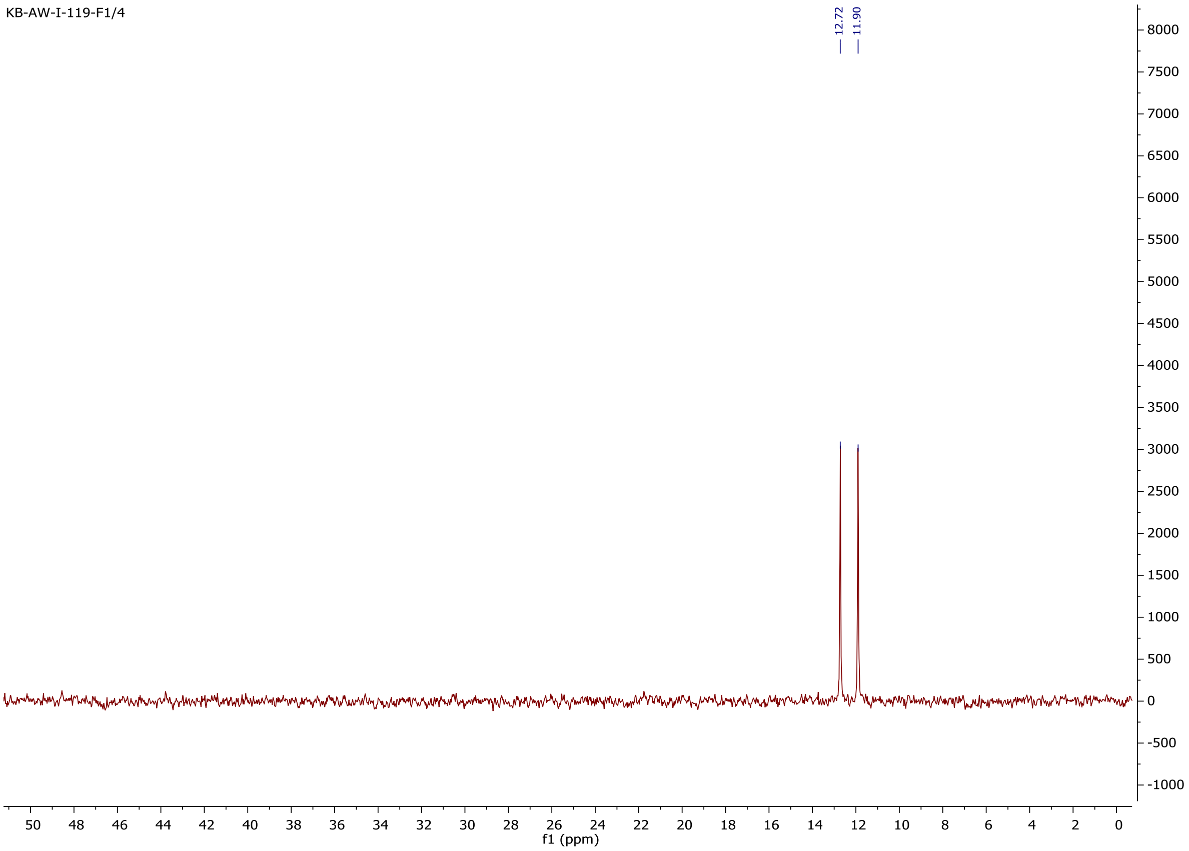

Figure S73. ^{31}P NMR of compound ***23*** *(101 MHz, CDCl$_3$)*

References

[1] Kaźmierczak, A.; Kusy, D.; Niinivehmas, S. P.; Gmach, J.; Joachimiak, Ł.; Pentikäinen, O. T.; Gendaszewska-Darmach, E.; Błażewska, K. M. Identification of the Privileged Position in the Imidazo[1,2-a]pyridine Ring of Phosphonocarboxylates for Development of Rab Geranylgeranyl Transferase (RGGT) Inhibitors. *J. Med. Chem.* **2017**, *60*, 8781-8800.
